# Can Natural Proteins Designed with ‘Inverted’ Peptide Sequences Adopt Native-Like Protein Folds?

**DOI:** 10.1371/journal.pone.0107647

**Published:** 2014-09-11

**Authors:** Settu Sridhar, Kunchur Guruprasad

**Affiliations:** Bioinformatics, Centre for Cellular and Molecular Biology, Hyderabad, Telangana, India; University of South Florida College of Medicine, United States of America

## Abstract

We have carried out a systematic computational analysis on a representative dataset of proteins of known three-dimensional structure, in order to evaluate whether it would possible to ‘swap’ certain short peptide sequences in naturally occurring proteins with their corresponding ‘inverted’ peptides and generate ‘artificial’ proteins that are predicted to retain native-like protein fold. The analysis of 3,967 representative proteins from the Protein Data Bank revealed 102,677 unique identical inverted peptide sequence pairs that vary in sequence length between 5–12 and 18 amino acid residues. Our analysis illustrates with examples that such ‘artificial’ proteins may be generated by identifying peptides with ‘similar structural environment’ and by using comparative protein modeling and validation studies. Our analysis suggests that natural proteins may be tolerant to accommodating such peptides.

## Introduction

The relationship between amino acid sequences to their corresponding three-dimensional structures continues to be of interest to biologists and bio-informaticians. In this context, we have earlier developed a relational database – PSSARD, that can be used to query the conformations associated with any given peptide sequence in proteins of known three-dimensional structure [1]. Later, we analyzed the conformations associated with hexa-peptide and longer sequences that occur as continuous amino acid repeats in proteins (CARPs) [2]. We also examined whether hepta-peptide and large sequences that entirely correspond to a helix, strand or coil conformation in one protein may be associated with a different conformation in another protein, thereby representing some of the ‘chameleon’ sequences in proteins [3]. In the present work, we analyze the conformations and other structural properties associated with ‘inverted’ peptide sequences in proteins. Particularly, we were interested in evaluating whether it would be possible to design ‘artificial’ proteins by incorporating ‘inverted’ peptide sequences in natural proteins and whether the artificial protein would retain the native-like protein fold? This would suggest that natural proteins may accommodate such peptides sequences. Proteins designed in this manner may be useful to compare, for instance, the physiological properties of the native protein *vis-à-vis* the artificial protein.

The occurrence of inverted repeats in proteins has been predicted long ago and was based on extensive search of internal regularities in the amino acid sequences using the genetic code and the relative frequencies of amino acid alternatives in homologous proteins [4]. Further, the question of whether an ‘inverted’ protein sequence (or a retro-protein) would fold to a well-defined native-like structure as natural proteins do and if so, whether the folded structure would be similar to the native conformation of the original protein has earlier been investigated [5]. These authors took the retro-protein sequence corresponding to the B domain of *Staphylococcal* protein A (retro-protein A) and using different secondary structure prediction methods and a lattice Monte Carlo simulation model conjectured that it would form a three-helix bundle structure. The topology of the retro-protein A was predicted to be similar to the native protein and this needed validation by experimental verification. This work was followed by the observation of a large number of examples of Inverse Sequence Similarity (ISS) up to 34% identity and self-inverse protein sequences in sequence and structure databases [6]. In order to evaluate the structural meaning of ISS in proteins, these authors' extracted more than 4000 sequences from the Protein Data Bank (PDB), inverted the sequences and searched for similarity of the inverted sequences in the PDB and SwissProt sequence databases. Their study demonstrated that a degree of ISS which normally would be highly significant for structurally related proteins was not sufficient to indicate structural similarity. Subsequently, many proteins in the PDB were shown to contain conspicuous ISS to each other [7]. These authors analyzed whether this ISS is related to structural similarity by carrying out a large-scale three-dimensional structural superposition of corresponding C-alpha atoms for the forwardly and inversely aligned proteins. While comparing proteins of less than 50% pair-wise sequence identity, only 0.5% of inversely aligned proteins had similar folds, whereas 9% of forwardly aligned proteins in the same range showed similar 3-D structures, supporting the view that the inversion of protein sequences in almost all cases lead to a different folding property of the protein. It was therefore suggested, that the inverted sequences were suitable as *protein-like* sequences for control purposes without relation to existing proteins. It was also shown that the short helices keep their conformation when the sequence is inverted suggesting that the folding of helices is only weakly dependent on the sequence order [7]. However, the relative abundance of inverse proteins was attributed to the occurrence of same repeat patterns and amino acid propensity of existing proteins sequences and it was pointed out that ISS cannot be considered as random and therefore the use of inverse sequences as negative set in experiments was cautioned [8]. Inverted repeats have also been observed in α-helical membrane protein structures [9].

Till date, there is not a single natural protein sequence in the database of known protein three-dimensional structure that corresponds entirely to an inverted sequence. Therefore, it is not clear yet whether completely inverted protein sequences would fold like natural proteins. In the present work, we have carried out an exhaustive analysis in order to identify all the ‘inverted’ peptide sequences in representative proteins of known three-dimensional structure. Further, we have evaluated the secondary structure conformations, solvent accessibility and number of residue neighborhood contacts corresponding to the inverted peptides. We exclude peptide sequences that correspond to palindromes or to continuous single amino acid repeats, as these peptides read the same regardless of the protein chain direction. We examined whether inverted peptides present along the same protein chain or on different chains of the same protein interact in tertiary structure. More importantly, we evaluated whether it would be possible to ‘swap’ peptide sequences in natural proteins with their corresponding ‘inverted’ peptide sequences and whether such ‘artificial’ proteins retain native-like overall protein fold? The present study demonstrates with few examples via protein structure analysis, comparative protein modeling and validation studies that certain peptide sequences in natural proteins may be inverted and the protein is likely to fold as in the native protein. Further, we provide a list of natural proteins containing potentially ‘swappable’ peptide sequences with ‘inverted’ peptide sequences.

## Materials and Methods

A representative dataset of proteins of known three-dimensional structure available in the Protein Data Bank (PDB) [10] was identified according to the PDB_SELECT program [11]. These correspond to protein crystal structures determined at ≤2.5 Å resolution and with pair-wise protein chain sequence identities ≤25%. A computer program was developed in order to identify penta-peptide and large sequences associated as ‘inverted’ peptide pairs (or INVPEPs) in the representative protein dataset. Starting with the N-terminal residue of the first sequence in the dataset, a ‘probe’ sequence corresponding to the first five amino acid residues (or 5-mer) was considered and a ‘target’ sequence was generated by inverting the probe peptide sequence. This ‘target’ sequence was then searched starting from the N-terminal residue of the first sequence in the dataset, in order to identify exact sequence ‘hits’ by sliding one-residue at a time along the protein sequence until the end of the sequence was reached. Next, the ‘target’ sequence was searched for identifying identical ‘hits’ in the next protein sequence and likewise in all the protein sequences in the dataset. For each ‘hit’ that was identified, the protein PDB code, protein chain, peptide sequence, start and end residue positions for the ‘probe’ and ‘target’ sequences in the proteins were recorded. The ‘probe’ and ‘target’ sequences identified in this manner constituted the ‘inverted peptide pair’ of sequences (or INVPEPs) in the corresponding proteins. Then a new 5-mer ‘probe’ sequence was defined, once again for the first protein sequence in the dataset, by sliding along the sequence with respect to its previous position by one residue. Accordingly, the corresponding new ‘target’ sequence was generated and with the new ‘target’ sequence, once again INVPEPs and their corresponding protein pairs were identified from all proteins in the dataset as described above. This process was repeated until all 5-mer ‘probe’ sequences in all protein sequences in the dataset were examined in order to identify the INVPEPs. In the same manner, the INVPEPs of varying sequence length, i.e., 6-mer, 7-mer, and so on, up to the largest sequence (1045 amino acid residues) in the dataset was searched. The redundant INVPEPs corresponding to a same protein PDB pairs were excluded and only the largest INVPEP pair was retained to represent the INVPEPs. The additional redundant INVPEPs that were identified when the ‘target’ was defined as ‘probe’ were excluded from the analysis. Also, sequences corresponding to palindromes and continuous single amino acid repeats identified in the process were excluded from further analysis. Likewise, INVPEPs present within other chains of the same protein were also analyzed in order to infer if they were within interacting distance (≤3.2 Å). The INVPEPs present in altogether different proteins were examined for the possibility of conducting ‘swap’ experiments via comparative protein modeling.

The secondary structure conformations were obtained from the PDB website (http://www.rcsb.org) and were calculated according to the DSSP program [12] based on the hydrogen-bonding patterns. Accordingly, the secondary structure conformations for the individual amino acid residues corresponding to the INVPEPs were assigned as; H (alpha helix), E (beta-strand), B (beta-bridge), T (turn), G (3/10-helix) and S (bend). The secondary structure conformation for amino acid residue(s) corresponding to a ‘coil’ conformation was assigned ‘C’ and those with missing ATOM records in the PDB was assigned ‘-‘ as described in PSSARD. The solvent accessibility values were derived according to the method described in [13] and obtained by executing the AREAIMOL program available in the CCP4 software program suite (version 6.4.0) [14]. The total number of residue neighborhood contacts for amino acid residues constituting the INVPEPs was evaluated using the NCONT program available in the CCP4 software suite [14] by defining a distance cut-off value ≤3.2 Å between interacting atoms. The average solvent accessibility values and the total number of residue neighborhood contact for the INVPEPs were evaluated by writing our own computer programs. In calculating the average solvent accessibility and the total number of residue neighborhood contacts, the environment of the INVPEP in the context of the entire protein complex including multiple chains was considered.

The selection of potential INVPEPs as ‘candidates’ for designing the ‘swap’ experiments were identified based on a ‘similar structural environment’ criterion corresponding to the INVPEPs in the proteins. For this purpose, we prepared a list of exclusive INVPEPs where the difference in solvent accessibility values in the corresponding protein pairs was ≤1 Å^2^ and where the difference in total number of residue neighborhood contacts was ≤2. In order to evaluate whether it would be possible to introduce ‘inverted’ peptides in proteins, we constructed three-dimensional models of the ‘artificial’ proteins using the comparative protein modeling program – MODELER [15]. MODELER is a sophisticated computer program that constructs three-dimensional models of proteins by the satisfaction of spatial restraints. It uses the knowledge of the template structure and the alignment of the model sequence to the sequence of the template. We selected certain examples for modeling artificial proteins that represented INVPEPs in helix, strand and coil conformation in proteins of known three-dimensional structure. The models were constructed using the native protein structure as template. The sequence of the ‘artificial’ protein differs from the native protein only in the region corresponding to the inverted peptide in the sequence alignment. The quality of the models was evaluated using the PROCHECK program [16]. The method of 3-D profiles [17] was used to evaluate the compatibility of the ‘fold’ to sequence, particularly, in the region corresponding to the INVPEPs. Inter-atomic clashes defined by a distance cut-off value ≤1 Å between the INVPEP atoms and between the INVPEP and rest of the protein atoms were examined by developing a computer program. All figures were drawn using PyMol [18].

## Results and Discussion

The analysis dataset comprised 3,967 representative protein chains corresponding to protein crystal structure data available in the Protein Data Bank. The list of the protein PDB codes is attached in supplementary data (Appendix S1). The number of inverted peptide pairs (or INVPEPs) that were observed varying in sequence length starting from penta-peptide onwards is shown in Table 1. The second column in the table contains the number of INVPEPs obtained after excluding the redundant INVPEPs, palindromes and continuous single amino acid repeats. The third column contains the number of INVPEPs for which the secondary structure conformation was calculated. Certain protein PDB files contain missing ‘ATOM’ records corresponding to the inverted peptides and therefore the secondary structure could not be calculated for all the INVPEPs. Accordingly, the fourth column contains the number of INVPEPs for which the solvent accessibility was calculated. The fifth column contains the number of INVPEPs for which the residue neighborhood contacts were calculated and these correspond to the INVPEPs where the difference in solvent accessibility values for the ‘probe’ and ‘target’ sequences was ≤1 Å^2^. The sixth column contains the number of unique non-redundant INVPEP sequences and the last column contains the number of unique protein PDB entries representing inverted peptide sequences in proteins.

**Table 1:** INVPEPs in 3967 representative protein chains from the Protein Data Bank (PDB).

INVPEP sequence Length (number of amino acid residues)	Number of non-redundant INVPEPs observed in representative PDB dataset	Number of INVPEPs of known secondary structure conformation	Number of INVPEPs for which solvent accessibility calculated	Number of INVPEPs with difference in solvent accessibility values ≤1 Å^2^	Number of unique INVPEPS in non-redundant PDB dataset	Number of unique PDB_IDs representing the INVPEPS
Five	148313	125397	124921	3520	93493	3760
Six	9005	5331	5297	168	8478	3495
Seven	10460	460	454	15	577	1378
Eight	995	40	40	1	91	493
Nine	1279	2	2	1	24	245
Ten	607	1	1	0	9	171
Eleven	107	0	0	0	3	109
Twelve	1	1	1	0	1	1
Eighteen	1	0	0	0	1	2

In order to evaluate whether it is possible to ‘swap’ a peptide sequence in a protein with its corresponding ‘inverted’ peptide sequence and whether the ‘artificial’ protein containing the swapped peptide would have a native-like fold, we selected certain natural proteins that contain inverted peptide sequences relative to each other in order to test our hypothesis. These proteins are shown in Table 2. It contains details of the protein PDB code/chain, ‘swappable’ peptide amino acid sequence, start and end residue positions of the peptide sequence in corresponding protein structure, solvent accessibility, number of residue neighborhood contacts and the secondary structure conformation in the individual proteins. The last column contains the secondary structure conformation corresponding to the inverted peptide that was modeled on the template protein shown in column 1. In all these protein pairs, the difference in solvent accessibility corresponding to the inverted peptide pair was ≤1 Å^2^ and the difference in number of residue neighborhood contacts was ≤2. The inverted peptides in these protein pairs are characterized by a ‘similar’ environment in the individual proteins and therefore qualify as ‘candidate’ peptides for conducting the ‘swap’ experiments. The ‘structurally similar environment’ is independent of the secondary structure conformation of the peptide and is defined only by the difference in solvent accessibility and the difference in number of residue neighborhood contact values.

**Table 2:** Examples of protein pairs containing inverted peptides with ‘similar structural environment’.

S.No.	PDB_ID (protein 1)	Location in protein 1 (peptide 1)	Sequence (peptide 1)	Solvent accessibility (peptide 1)	Number of neighbourhood residues (peptide 1)	Secondary structure (peptide 1)	PDB_ID (protein 2)	Location in protein (peptide 2)	Sequence (peptide 2)	Solvent accessi-bility (peptide 2)	Number of neighbourhood residues (peptide 2)	Secondary structure (peptide 2)
1	3BGY:A	146–150	STEEI	31.76	8	EEEEE	3FWK:A	121–125	IEETS	31.58	10	HHHHH
2	2PKH:H	125–129	ERALA	72.3	5	HHHHH	3E1I:A	168–172	ALARE	71.6	4	CCCCC
3	2OC5:A	180–185	LEANRE	64.4	8	HHHHHH	3HE4:B	15–20	ERNAEL	63.82	10	HHHHHH
4	1OUW:D	42–46	PIALT	6.72	10	EEEEE	1OUW:D	761–765	TLAIP	5.76	10	EEEEE

**Figure 1:**
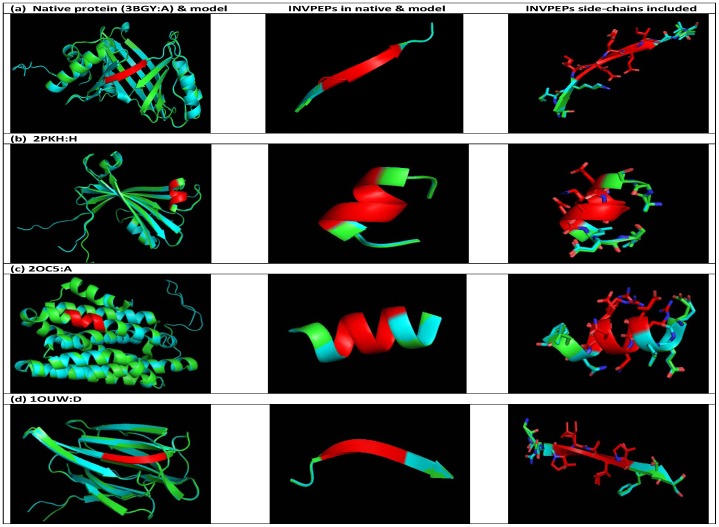
Schematic representation showing structural overlay of the ‘artificial’ proteins (cyan) modeled on the PDB templates (green); (a) 3BGY_A, (b) 2PKH_H, (c) 2OC5_A and (d) 1OUW_D. The secondary structure conformations corresponding to the inverted peptide, its corresponding peptide in the native protein, and the peptide amino acid residue side-chains are shown in the panels alongside.

In order to test our hypothesis, we selected native protein ‘good’ quality structures according to the Ramachandran plot for conducting the ‘swap’ experiment. A ‘good’ quality protein structure is expected to have more than 90% amino acid residues in the ‘allowed’ region of the Ramachandran plot. All the four proteins (labeled protein 1) shown in Table 2 satisfy the above criterion. We then constructed three-dimensional models of the ‘artificial’ proteins by ‘swapping’ the sequence of peptide 1 with its ‘inverted’ peptide sequence using the MODELER program. The quality of the models was verified using the PROCHECK program. Further, we also analyzed the ‘compatibility’ of the inverted peptide sequence to structure using the Verify 3-D program. The Verify 3-D program evaluates not only the correctness of the overall protein fold but also detects ‘incorrectly’ folded regions in an otherwise correctly folded protein. The S-score for the ‘correct’ fold is expected to be ≥ S-score for the ‘incorrect’ fold. Accordingly, the 3-D profile scores in the model corresponding to the inverted peptide sequence must be either greater than or equal to the equivalent scores for the corresponding peptide in the native protein. Among eighteen ‘artificial’ proteins that were modeled only models that satisfy the compatibility of the sequence to the fold according to 3-D profile criterion are shown in Table 2. In all four ‘artificial’ proteins containing inverted peptides that were modeled on the corresponding native protein structure template (labeled as protein 1 in Table 2), all the amino acid residues are in ‘most favoured’ or ‘additional allowed’ regions and none in the ‘disallowed’ regions of the Ramachandran plot suggesting these models also are of ‘good’ quality. The Ramachandran plots for the native and modeled structures are shown in Appendix S2. Further, the combined values of the 3-D profile scores corresponding to the inverted peptide in the ‘artificial’ protein is larger than the value for the equivalent peptide in the native protein suggesting that the artificial protein containing the inverted peptide sequence is also compatible to the protein fold. The combined values of the 3-D profile scores for the native and modeled proteins are shown in supplementary data (Appendix S3). Taken together these criteria suggest that it may be possible to introduce ‘inverted’ peptides in natural proteins and the ‘artificial’ protein containing the inverted peptide sequence is likely to adopt the native-like protein fold as shown for the examples in Figure 1. This figure illustrates the overall structural similarity of some of the native and artificial proteins. The secondary structure conformation corresponding to the native and inverted peptide is highlighted. The side-chains of the native and inverted peptides are also shown.

Based on the above analysis, we suggest the following protocol in order to assess whether a peptide sequence in a protein of known three-dimensional structure may be swapped with its inverted peptide sequence and the protein retain native-like fold. Select a short peptide sequence (five to seven amino acid residues) in a protein that represents a ‘good’ quality structure. Invert the sequence and model the artificial protein containing the inverted peptide sequence on the template of the native protein structure using MODELER. Evaluate the solvent accessibility and the number of residue neighborhood contacts for the peptides in the native and modeled structures. If the two proteins share ‘similar structural environment’ as described earlier, then assess the overall quality of the model and the ‘compatibility’ of the inverted peptide sequence to the fold. Inverted peptides that do not result in ‘good’ quality models according to PROCHECK criterion or those that are incompatible to the protein fold according to the 3-D profile scores criterion must be avoided. Also, to ensure there are no steric clashes involving the inverted peptide atoms. Further, inverted peptides that are directly involved in the protein molecular function must be avoided. The inverted peptides that satisfy these conditions may be selected as ‘potential’ candidate for the ‘swap’ and the ‘artificial’ proteins designed in this manner are likely to retain native-like protein folds.

In our analysis, we observed that the INVPEPs in proteins are associated with the helix, strand, coil conformation or combination of these conformations. Among these, the number of INVPEPs entirely associated with a helix, strand or coil conformation is shown in Table 3. The INVPEPs in the helix conformation are predominant suggesting they are potential ‘swappable’ candidates. This is confirmed in the examples that we modeled (shown in Table 2) and is in agreement with previous observations that helices are much more stable to inversion than other secondary structural elements [7]. However, in the present study, we observed INVPEPs also in the ‘strand’ conformation suggesting that peptides in strand conformation may also be stable to inversion. Whereas, INVPEPs in ‘coil’ conformation are rare suggesting that swapping a peptide in ‘coil’ conformation in a protein with its equivalent inverted peptide may be avoided. We further observed by examining on the graphics that the individual native peptide sequences related as ‘inverted’ peptides and occurring in natural proteins were generally distantly located in the protein three-dimensional structure. Further, a number of ‘overlapping’ INVPEPs were observed, for instance, INVPEPs in the triple-helical structures of the collagen peptide (PDB code: 3A0M, C-chain, between positions 16–27 and 15–26). We excluded such INVPEPs from our analysis as they did not represent ‘distinct’ peptide sequences.

**Table 3:** Total number of INVPEPs observed in helix (H), strand (E) and coil (C) conformation among 1625 ‘swappable’ INVPEPS.

	H	E	C
H	223	36	3
E	41	40	0
C	1	0	0

We are providing a list of 1625 natural protein pairs selected from representative protein crystal structures in the Protein Data Bank that contain potentially ‘swappable’ inverted peptide candidate sequences (Appendix S4). These inverted peptide pair of sequences were not observed in any of the other proteins analyzed, where the difference in solvent accessibility and the number of residue neighborhood contact values exceeded the corresponding limits we have considered for identifying the ‘swappable’ peptides. These peptide sequences may serve as probable ‘leads’ for the design of ‘artificial’ proteins. The largest INVPEPs of known structure comprised nine amino acid residues. The protein crystal structure of *E.coli* dihydroneopterin aldolase in complex with neopterin (PDB code:2O90A) contains the sequence ‘VERVAEEVA’ between positions 72 to 80. Another protein that corresponds to the crystal structure of a histidine triad (hit) protein (mfla_2506) from methylobacillus flagellates kt (PDB code:2OIKD) contains the equivalent inverted peptide sequence ‘AVEEAVREV’ between positions 70 to 78. The peptides in both these proteins are in a helix conformation and are related by a ‘structurally similar environment’. However, the first two residues ‘VE’ of the sequence in *E.coli* dihydroneopterin aldolase are involved in binding the ligand L-neopterin and therefore swapping the peptide in this protein with its inverted peptide may not be desirable.

In summary, in the present work, we have selected a high-quality representative dataset of proteins of known three-dimensional crystal structures available in the Protein Data Bank and catalogued all penta-peptide and large sequences that are observed as ‘inverted’ peptides in proteins. Further, we have evaluated their secondary structure, solvent accessibility and number of residue neighborhood contacts in the proteins. In particular, we have analyzed whether it would be possible to ‘swap’ peptides in proteins with their corresponding ‘inverted’ peptide sequences and evaluate whether the ‘artificial’ protein generated would retain the native-like protein fold. In order to test our hypothesis, we selected few protein pairs that are known to contain inverted peptides sequences relative to each other and characterized with a ‘similar structural environment’ defined by the difference in solvent accessibility and number of residue neighborhood contacts. These protein pairs provide the test cases for evaluating whether ‘artificial’ proteins generated by swapping the relevant peptide with its inverted peptide sequence would fold as in the native proteins. The proteins incorporating the inverted peptides were modeled using the MODELER software and validated for the satisfaction of model quality as well as the compatibility of the inverted sequence to the protein fold. The modeled proteins that satisfy these criteria suggested that it may be possible to design ‘artificial’ proteins by incorporating inverted peptide sequences that are expected to retain the native-like protein fold. This enabled us to suggest a protocol for designing inverted peptides in natural proteins. However, caution needs to be exercised in selecting inverted peptides where the amino acid residues are known to be directly involved in the protein function. The structural similarity observed for some of the artificial proteins to their corresponding native proteins provides the basis for further validation by wet-lab experiments. Designing proteins with ‘inverted’ peptide sequences could be a way of generating ‘artificial’ proteins that mimic natural protein folds that may find suitable applications in modern biology.

## Conclusions

Natural proteins may be tolerant to accommodating certain short ‘inverted’ peptide sequences that are likely to adopt native-like protein folds. Swapping peptide sequences in proteins of known three-dimensional structure with inverted peptides could be one way of generating ‘artificial’ proteins without altering the protein amino acid composition.

## Supporting Information

Appendix S1 **List of proteins used in the analysis of inverted peptides.**
(TXT)

Appendix S2 **Ramachandran plots for native and modelled structures using the PROCHECK software.**
(DOC)

Appendix S3 **Verify 3-D plots and scores for native and modelled structures corresponding to the inverted peptide.**
(DOC)

Appendix S4 **Potentially ‘swappable’ inverted peptides list.**
(PDF)

## References

[1] Guruprasad K, Srikanth K, Babu AVN (2005) PSSARD: Protein Sequence-Structure Analysis Relational Database. Int J Biol Macromol 36: 259–262. 16054209 10.1016/j.ijbiomac.2005.06.005

[2] Gayatri M, Guruprasad K (2010) Analysis of the conformations corresponding to hexapeptide and large sequences characterized by continuous single amino acid repeats in proteins. Protein Pept Lett 17: 1459–1465. 20937037 10.2174/0929866511009011459

[3] Krishna N, Guruprasad K (2011) Certain heptapeptide and large sequences representing an entire helix, strand or coil conformation in proteins are associated as chameleon sequences. Int J Biol Macromol 49: 218–222. 21569793 10.1016/j.ijbiomac.2011.04.017 PMC7124434

[4] Wuilmart C, Wijns L, Urbain J (1975) Linear and inverted repetitions in protein sequences. J Mol Evol 5: 259–278. 1202227 10.1007/BF01732214

[5] Olszewski KA, Kolinski A, Skolnick J (1996) Does a backwardly read protein sequence have a unique native state? Protein Eng 9: 5–14. 9053902 10.1093/protein/9.1.5

[6] Preissner R, Goede A, Michalski E, Frömmel C (1997) Inverse sequence similarity in proteins and its relation to the three-dimensional fold. FEBS Lett 414: 425–429. 9315733 10.1016/s0014-5793(97)00907-1

[7] Lorenzen S, Gille C, Preissner R, Frömmel C (2003) Inverse sequence similarity of proteins does not imply structural similarity. FEBS Lett 545: 105–109. 12804758 10.1016/s0014-5793(03)00450-2

[8] Nebel JC, Godfrey C, Walawage C (2010) Why inverse proteins are relatively abundant. Protein Pept Lett 17: 854–860. 20205652 10.2174/092986610791306698

[9] Pornillos O, Chang C (2006) Inverted repeat domains in membrane proteins. FEBS Lett 580: 358–362. 16406365 10.1016/j.febslet.2005.12.054

[10] Bernstein FC, Koetzle TF, Williams GJ, Meyer EE Jr, Brice MD, et al (1977) The Protein Data Bank: A Computer-based Archival File For Macromolecular Structures. J Mol Biol 112: 535–542. 875032 10.1016/s0022-2836(77)80200-3

[11] Griep S, Hobohm U (2010) PDBselect 1992–2009 and PDBfilter-select Nucleic Acids Res. 38: D318–D319. 10.1093/nar/gkp786 PMC2808879 19783827

[12] Kabsch W, Sander C (1983) Dictionary of protein secondary structure: pattern recognition of hydrogen-bonded and geometrical features. Biopolymers 22: 2577–2637. 6667333 10.1002/bip.360221211

[13] Lee B, Richards FM (1971) The interpretation of protein structures: estimation of static accessibility. J Mol Biol 55: 379–400. 5551392 10.1016/0022-2836(71)90324-x

[14] Winn MD, Ballard CC, Cowtan KD, Dodson EJ, Emsley P, et al (2011) Overview of the CCP4 suite and current developments. Acta Cryst D67: 235–242. 10.1107/S0907444910045749 PMC3069738 21460441

[15] Sali A, Blundell TL (1993) Comparative protein modelling by satisfaction of spatial restraints. J Mol Biol 234: 779–815. 8254673 10.1006/jmbi.1993.1626

[16] Laskowski RA, MacArthur MW, Moss DS, Thornton JM (1993) PROCHECK: a program to check the stereochemical quality of protein structures. J Appl Cryst 26: 283–291.

[17] Lüthy R, BowieJU, Eisenberg D (1992) Assessment of protein models with three-dimensional profiles. Nature 356: 83–85. 1538787 10.1038/356083a0

[18] The PyMOL Molecular Graphics System, Version 1.5.0.4 Schrödinger, LLC.

