# Supplementary figures and images for: Can Natural Proteins Designed with ‘Inverted’ Peptide Sequences Adopt Native-Like Protein Folds?
Source: PLoS One. 2014 Sep 11;9(9):e107647. doi: 10.1371/journal.pone.0107647 (PMC4161436; doi:10.1371/journal.pone.0107647)

1.

| **3BGY:A native** | **3BGY:A model** |
| --- | --- |
|  |  |

**2.**

| **2PKH:H native** | **2PKH:H model** |
| --- | --- |
| **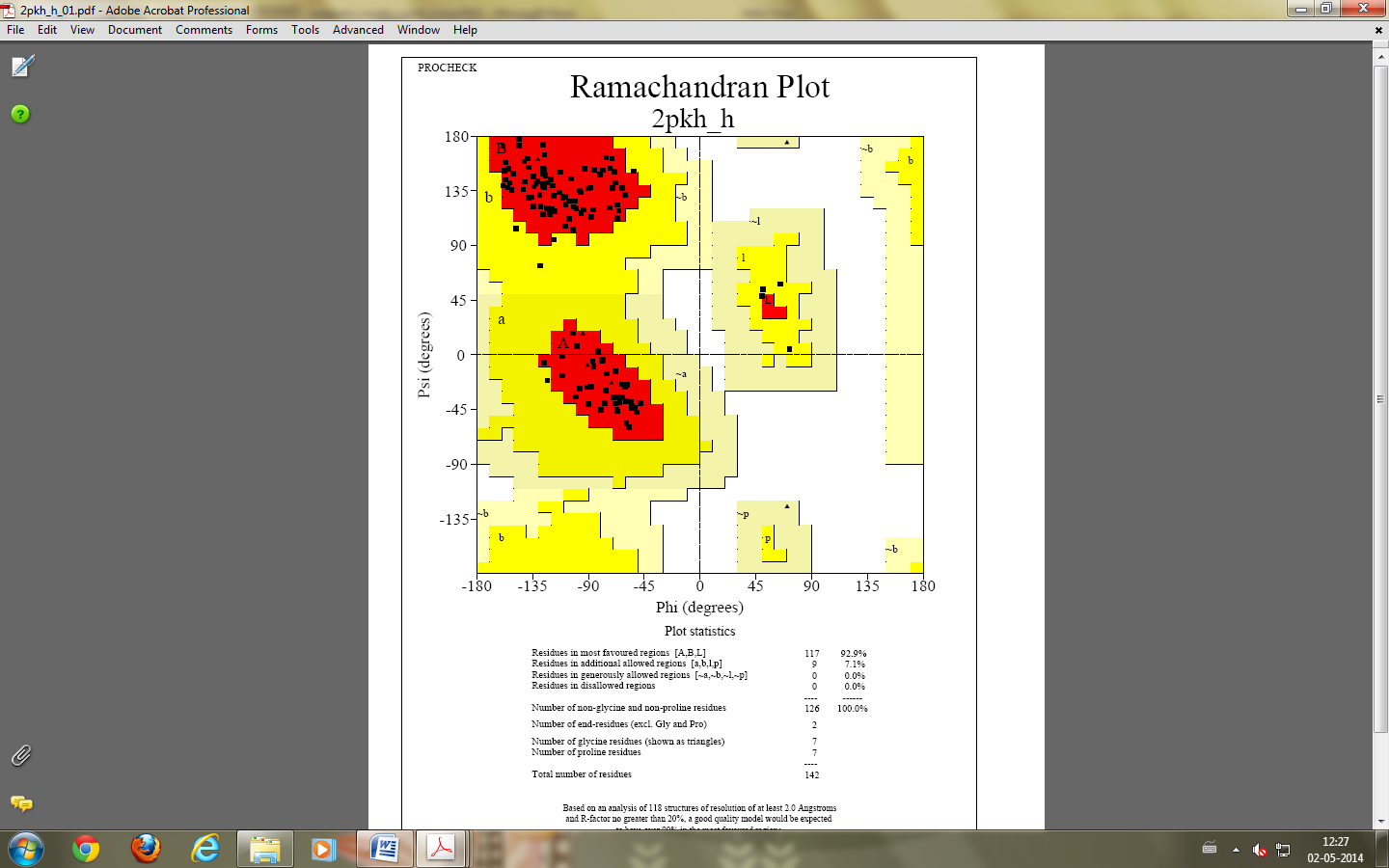** | **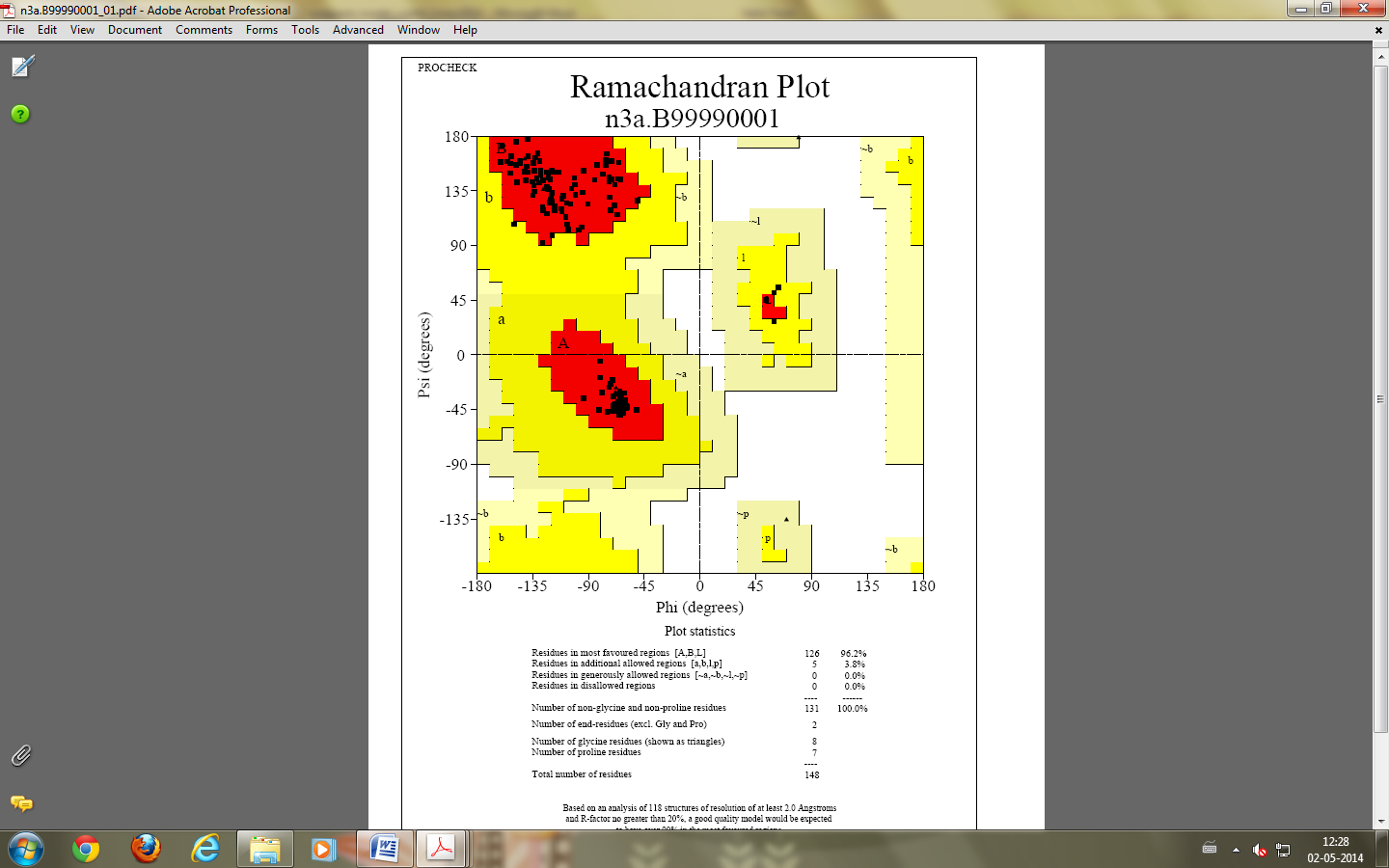** |

**3.**

| **2OC5:A native** | **2OC5:A model** |
| --- | --- |
|  | 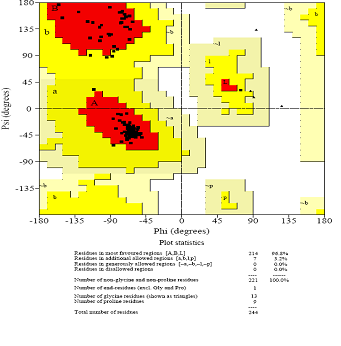 |

4.

| **1OUW:D native** | **1OUW:D model** |
| --- | --- |
| 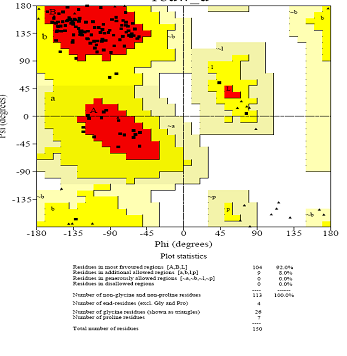 | 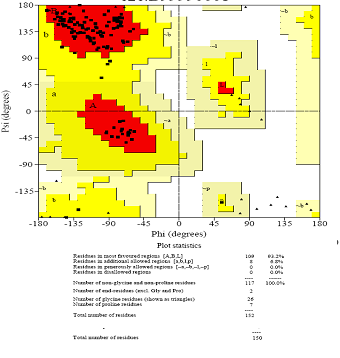 |

Supplement: Appendix S2 — Ramachandran plots for native and modelled structures using the PROCHECK software. (DOC) [file pone.0107647.s002.doc]
