# Supplementary material for: Can Natural Proteins Designed with ‘Inverted’ Peptide Sequences Adopt Native-Like Protein Folds?
Source: PLoS One. 2014 Sep 11;9(9):e107647. doi: 10.1371/journal.pone.0107647 (PMC4161436; doi:10.1371/journal.pone.0107647)
Supplement: Appendix S3 — Verify 3-D plots and scores for native and modelled structures corresponding to the inverted peptide. (DOC) [file pone.0107647.s003.doc]

1.

| **3BGY:A 146-150 STEEI Verify3D score: 0.74** | **3BGY:A_mdl IEETS Verify3D score: 1.78** |
| --- | --- |
| **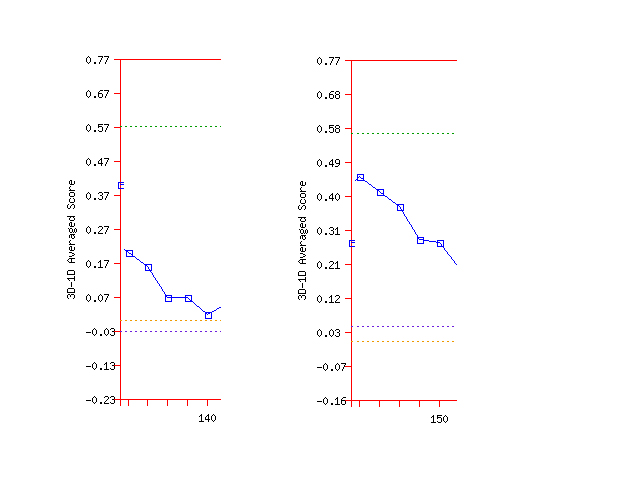** | |

2.

| **2PKH:H 125 – 129 ERALA Verify3D score: 1.25** | **2PKH:H_model ALARE Verify3D score: 2.04** |
| --- | --- |
| **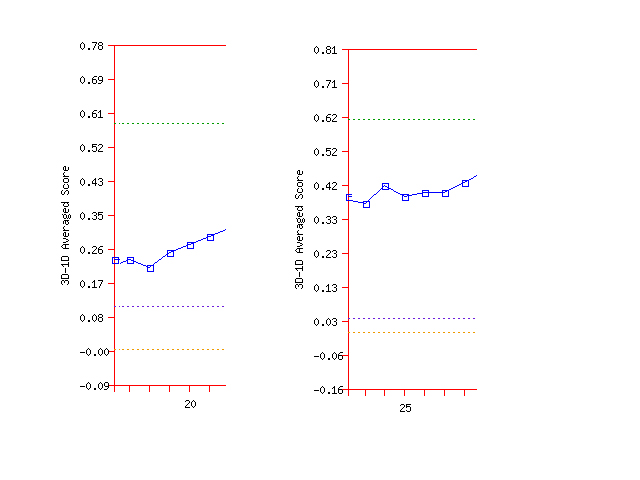** | |

3.

| **2OC5:A 180-185 LEANRE Verify3D score: 0.99** | **2OC5:A_mdl ERNAEL Verify3D score: 1.34** |
| --- | --- |
| **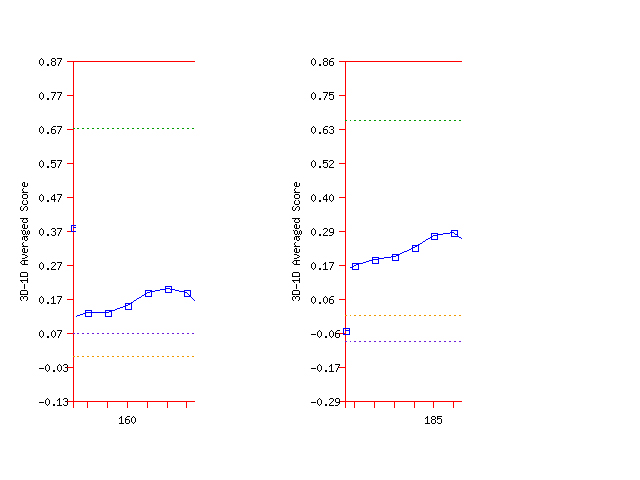** | |

4.

| **1OUW:D 42-46 PIALT Verify3D score: 3.09** | **1OUW:D_model TLAIP Verify3D score: 3.54** |
| --- | --- |
| **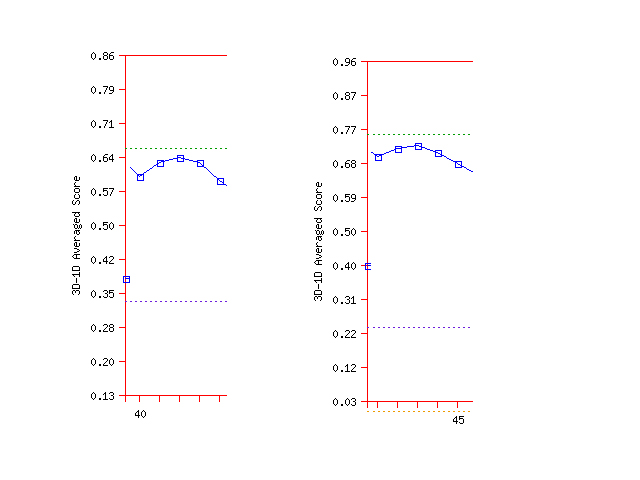** | |
