# Supplementary material for: Can Natural Proteins Designed with ‘Inverted’ Peptide Sequences Adopt Native-Like Protein Folds?
Source: PLoS One. 2014 Sep 11;9(9):e107647. doi: 10.1371/journal.pone.0107647 (PMC4161436; doi:10.1371/journal.pone.0107647)
Supplement: Appendix S4 — Potentially ‘swappable’ inverted peptides list. (PDF) [file pone.0107647.s004.pdf]

PDB_ID	Sequence	Start	End	Solvent accessibility	Neighbourhood residue contacts	Secondary structure	PDB_ID	Sequence	Start	End	Solvent accessibility value	Neighbourhood residue contacts	Secondary structure
1A1X:A	NNSRL	81	85	72.58	5	TTSCE	2Q00:B	LRSNN	72	76	73.38	7	GGGTC
1ALU:A	LPKMA	64	68	50.36	5	CCCCC	3K1E:A	AMKPL	18	22	49.36	7	HHHHH
1ARB:A	SSGSP	193	197	3.76	9	CTTCE	3SGG:A	PSGSS	389	393	3.48	10	TTSSS
1ARB:A	ASGAN	87	91	46.96	8	GGGSC	3S83:A	NAGSA	479	483	46.3	6	CHHHH
1ARB:A	PASGA	86	90	47.22	7	GGGGS	1O9G:A	AGSAP	165	169	47.18	5	TTCCC
1ARB:A	CPEGD	12	16	50.44	7	SGGGT	1JW9:B	DGEPC	168	172	50.74	5	TTCCC
1AY7:B	ESVLQ	68	72	35.54	7	HHHHH	3D3S:D	QLVSE	17	21	36.06	8	HHHHT
1B0B:A	AVAGA	129	133	25.28	7	HHHHH	3TKT:A	AGAVA	215	219	25.64	8	HHHHH
1B0B:A	ALFDA	31	35	43.72	6	HHHHH	2PD1:D	ADFLA	21	25	43.6	6	HHHHH
1B0B:A	NAGAL	81	85	42.62	6	CHHHH	1PA2:A	LAGAN	131	135	42.14	4	HHHHH
1B0U:A	QLAEE	198	202	61.8	7	HHHHT	2CF5:A	EEALQ	218	222	62	6	HHHHT
1B5E:A	QVASS	84	88	58.48	7	HHSCT	1URS:A	SSAVQ	334	338	58.36	6	SHHHH
1BD3:B	EKLPA	149	153	53.3	5	EECCT	2H00:C	APLKE	219	223	53.48	5	HHHHH
1BD3:B	RMVTA	213	217	12.72	11	EEEEE	3CTP:A	ATVMR	244	248	11.84	9	HHHHH
1BGC:A	APLSS	60	64	42.26	7	CCCGG	2CXY:A	SSLPA	35	39	41.32	7	CBCCE
1BGF:A	CLREE	108	112	41.74	10	HHHHH	1A79:C	EERLC	82	86	41.56	11	CTTHH
1BI5:A	AAVKA	109	113	32.6	10	HHHHH	2IXD:B	AKVAA	57	61	32.32	11	HHHHH
1BI5:A	VVLRS	382	386	21.22	9	EEEEE	3MQ2:A	SRLVV	48	52	22.16	11	TEEEE
1BI5:A	TKALV	280	284	52.9	5	HHHHH	2HCJ:A	VLAKT	34	38	52.7	7	HHHHH
1BYI:A	SLQLD	63	67	74.86	5	SSCCC	3NO3:A	DLQLS	93	97	74.5	7	TCBCT
1C44:A	GKAKL	119	123	80.2	4	CCCCC	1VJQ:B	LKAKG	57	61	81.14	4	HHHHT
1CCW:A	RFKDM	107	111	62.82	8	HHHHT	1EJD:A	MDKFR	1	5	62.12	8	CEEEE
1CMC:B	VLKIL	32	36	35.1	7	HHHHH	3FA2:A	LIKLV	691	695	34.4	8	HHHHH
1CV8:A	NKLEN	7	11	67.38	6	EECTT	3IU0:A	NELKN	138	142	68.2	5	HHHHH
1D4O:A	GDAKK	165	169	51.76	6	SCHHH	1TZD:B	KKADG	334	338	52.6	5	ECSSS
1D8D:A	EILAK	342	346	54.24	8	HHHHH	3V2U:D	KALIE	483	487	54.28	8	HHHHH
1DCS:A	TFSVP	272	276	31.6	8	EEEHH	1KWI:A	PVSFT	75	79	32.46	9	EEEEE
1DCS:A	RQYTA	125	129	35.34	12	HHHHH	3H9M:A	ATYQR	200	204	36.04	10	HHHHH
1DYO:A	FEGSV	15	19	52.4	5	CSSCC	3LQB:A	VSGEF	26	30	53.06	4	ECTTS
1DZK:B	IGENA	29	33	40.02	6	GSTTS	3CZQ:A	ANEGI	184	188	39.88	7	HHHTC

1E7S:A	RIAGI	140	144	12.36	9	HHHHH	1W5Q:A	IGAIR	182	186	12.96	9	HHHHH
1EB6:A	AAESG	31	35	45.6	7	HHHHS	3H9M:A	GSEAA	53	57	45.94	5	CCSEE
1ECA:A	NIEAD	75	79	57.92	9	CCHHH	2DTJ:B	DAEIN	39	43	57.44	8	HTTCC
1EF1:B	TAVLL	121	125	4.76	7	HHHHH	3B7F:A	LLVAT	24	28	5.5	9	EEEEE
1EJD:A	ATVTI	165	169	2.1	9	HHHHH	3K5I:B	ITVTA	357	361	1.3	10	EEEEC
1EJD:A	LPILF	26	30	2.26	8	HHHHH	1SBQ:B	FLIPL	102	106	2.58	8	EEECC
1EJD:A	RLKGA	150	154	55.88	8	SCBCC	2V6V:B	AGKLR	336	340	56.84	7	TTCCC
1EK6:A	FNPTG	186	190	20.86	7	CEEEC	3AKH:A	GTPNF	307	311	20.22	7	SCEEC
1EK6:A	SLAQE	312	316	62.38	7	HHHHH	2GPI:A	EQALS	52	56	63.04	9	HHHHH
1ES9:A	VNELV	155	159	24.68	9	HHHHH	1B34:B	VLENV	66	70	23.7	10	EEEEE
1ES9:A	AEQVT	110	114	35.8	6	HHHHH	1YD7:A	TVQEA	151	155	35.94	5	SHHHH
1ES9:A	VNELV	155	159	24.68	9	HHHHH	1B0U:A	VLENV	110	114	24.16	7	HHHHH
1EXR:A	DIDGDG	129	134	62.22	5	CSSSSS	2NR7:A	GDGDID	47	52	62.63	5	TSTTCC
1F00:I	NQEVT	693	697	48	9	TCEEE	3P6B:B	TVEQN	150	154	47.24	11	EEEEE
1F0L:B	GANYAA	275	280	20.93	8	HHHHHH	1QSA:A	AAYNAG	550	555	21.07	8	HHHHHC
1F0L:B	VAQAI	340	344	11.34	8	HHHHT	1QCA:A	IAQAV	63	67	11.24	7	HHHHH
1F0L:B	ELSEL	259	263	39.64	9	GGHHH	2GNP:A	LESLE	74	78	40.34	8	CSEEE
1F1E:A	KDTIY	28	32	35.36	8	HHHHH	2NW2:A	YITDK	159	163	34.56	9	EECCC
1F5V:B	GALAQ	187	191	51.28	6	HHHHH	1BGC:A	QALAG	91	95	51.38	8	HHTTT
1F61:A	VELQE	374	378	36.8	9	HHHHH	2ZSI:B	EQLEV	51	55	37.26	9	HHHHH
1FAS:A	RKSRR	24	28	51.82	14	EEEES	2FTB:A	RRSKR	120	124	52.72	12	EEEEE
1FC3:C	PTNSE	239	243	51.6	7	CCHHH	1MKF:B	ESNTP	112	116	51.2	6	ECCCC
1FJ2:A	IDQEV	95	99	40.8	9	HHHHH	1EDY:A	VEQDI	98	102	41.52	7	EEESS
1FVK:A	GDLGK	66	70	64.56	5	HHHHH	2UUR:A	KGLDG	135	139	65.56	4	EBTTS
1FXK:C	IKKNF	86	90	40.06	10	EEEEH	2VRN:A	FNKKI	172	176	40.44	9	HHHHH
1G2R:A	KKKKVF	60	65	65.53	8	HHHTHH	2IU1:A	FVKKKK	246	251	66.15	7	HHHHHH
1G2Y:D	SKLSQ	3	7	68.34	5	CHHHH	2R4G:A	QSLKS	338	342	68.2	6	HHHHH
1G5H:C	SIREI	126	130	61.86	6	HHHHH	3IJD:B	IERIS	38	42	61.96	6	HHHHH
1G5H:C	GKGPT	362	366	62.62	7	CSSCH	1OI7:A	TPGKG	39	43	62.76	5	CTTCT
1G66:A	VGYSQG	87	92	2.55	34	EEETHH	3LO8:A	GQSYGV	69	74	2.42	32	TCEEEE
1G6X:A	FVYGG	33	37	26.88	8	EEECS	1URS:A	GGYVF	189	193	26.72	7	TCEEE
1GA6:A	FDAAQ	264	268	17.42	10	EECCG	3F0H:A	QAADF	267	271	17.66	9	HHHHH
1GA6:A	QTIQT	60	64	56.9	7	EEEEC	3P8K:A	TQITQ	20	24	56.3	9	HHHHH

1GA6:A	VIAVG	198	202	0.16	11	SEEEE	3GQH:A	GVAIV	847	851	1.02	9	EEEEE
1GKM:A	SVACV	38	42	17.74	10	HHHHH	1TH8:B	VCAVS	80	84	18.3	11	EESCC
1GKM:A	SLPTS	406	410	65.46	6	CBTTT	3KYZ:A	STPLS	93	97	65.4	7	SSCCC
1GKM:A	GVLAI	436	440	1.64	8	HHHHH	3QA9:A	IALVG	22	26	2.6	8	EEEET
1GKM:A	NGGVN	374	378	78.6	3	TTCCT	2Y3C:A	NVGGN	161	165	79.36	3	EETTE
1GKM:A	AIAEI	344	348	20.7	8	HHHHH	2ZKM:X	IEAIA	388	392	19.9	7	HHHHH
1GKM:A	QLDAS	26	30	67.08	6	EECGG	1XL3:A	SADLQ	240	244	66.44	8	HHHHH
1GKP:E	ALAQG	303	307	16.1	7	HHHTT	1T61:A	GQALA	155	159	15.12	8	ECCTT
1GKP:E	LLIKNG	3	8	26.42	8	EEEEEE	3OJ0:A	GNKILL	18	23	25.7	9	CCEEEE
1GKP:E	VVTVR	428	432	5.82	12	EEEET	1G8M:A	RVTVV	142	146	5.82	11	TCEEE
1GKP:E	VTVRG	429	433	8.52	10	EEETT	1FM0:E	GRVTV	77	81	8.52	9	EEEEE
1GL2:C	SIERS	145	149	42.58	9	HHHHH	3FFY:A	SREIS	184	188	43.42	9	EEESS
1GMX:A	VADAH	9	13	30.34	7	HHHHH	3GKM:A	HADAV	146	150	30.58	8	HHHHH
1GQ1:B	VDATL	456	460	1.84	10	ECCTT	3TQE:A	LTADV	68	72	1.9	10	HHHHH
1GQ1:B	LKTTE	333	337	34.06	10	EEEEE	3ALJ:A	ETTKL	271	275	33.9	10	EEEEE
1GU2:A	KVEDE	87	91	73.6	8	HHHHH	3LFJ:B	EDEVK	125	129	72.7	6	HHHHH
1GVE:B	LSNYV	138	142	4.3	11	EESCC	2BKY:Y	VYNSL	49	53	5.06	11	HHHHH
1GVP:A	KVEIK	3	7	48.46	10	CEEEC	2ZXE:B	KIEVK	300	304	48.38	8	EEEEC
1GWE:A	STVAG	99	103	4.22	8	ECSSS	2VFR:A	GAVTS	158	162	3.4	8	GTSSC
1GXM:B	AATNPI	580	585	39.8	5	TTTCCE	1L2U:A	IPNTAA	77	82	38.88	6	CHHHHH
1GXM:B	SVGNG	375	379	70.9	5	TCCCC	3M7P:A	GNGVS	337	341	71.4	4	TTEEE
1GXR:B	TISNP	491	495	44.64	7	EECSS	3GRE:A	PNSIT	1080	1084	44.06	9	TSCEE
1GXU:A	LARID	68	72	72.8	5	TCEEE	2VDJ:A	DIRAL	32	36	73.12	5	TSCCE
1GZ8:A	IVKLL	63	67	31.84	6	BCCEE	3OII:A	LLKVI	159	163	32.72	8	EEEEE
1H16:A	AVDLV	577	581	13.02	9	HHHHH	3GXB:B	VLDVA	1496	1500	13.84	9	CEEEE
1H16:A	MLYAI	442	446	1.06	10	HHHHH	1ZZW:A	IAYLM	420	424	1.92	8	HHHHH
1H1N:A	EFAGG	240	244	2.32	9	EEECC	1EF8:A	GGAFE	109	113	2.1	9	THHHH
1H41:B	GSLPN	180	184	19.64	6	TTTTT	1F0L:B	NPLSG	71	75	20.3	7	STTTS
1H41:B	VQERE	651	655	29.74	12	HHHHH	2WKJ:C	EREQV	58	62	29.4	13	HHHHH
1H41:B	VVIVA	113	117	1.24	8	EEEEE	3K5I:B	AVIVV	191	195	1.24	7	EEEEE
1H41:B	LQHRV	152	156	22.9	12	CSEEE	3AG3:L	VRHQL	40	44	23.24	11	HHHHH
1H4X:B	DATEE	101	105	73.28	9	CCCHH	3U7Z:B	EETAD	88	92	72.3	10	TEECC
1H4X:B	LLNPS	79	83	9.3	10	EESCC	3V4H:A	SPNLL	66	70	8.76	8	HHHHH

1H72:C	DKVYG	251	255	42	8	TTEEE	1Y42:X	GYVKD	75	79	42.48	7	TCCSC
1H7E:A	EAAEP	133	137	58.74	6	HHTCT	2OX0:B	PEAAE	348	352	59.5	4	GGGGG
1H97:A	MLKEIS	77	82	37.92	7	HHTTTT	3K1W:B	SIEKLM	238	243	37.4	8	HHHHHH
1H97:A	EQDIL	6	10	30.36	9	HHHHH	1FJ2:A	LIDQE	94	98	31.36	9	HHHHH
1HBN:A	VLDVA	370	374	13.96	10	HHHHH	1H16:A	AVDLV	577	581	13.02	9	HHHHH
1HBN:F	GDELD	192	196	59.04	6	SCEEE	3FLE:B	DLEDG	227	231	59.46	7	ECCSS
1HBN:F	RSRAY	101	105	2.86	14	HHHHH	1G8M:A	YARSR	304	308	3.84	14	HHHHH
1HFO:F	GGIEP	65	69	39.08	8	SSCCH	2C5S:A	PEIGG	126	130	39.42	8	HHHHH
1HFO:F	LNTKL	83	87	46.3	7	HHHHH	3CU2:B	LKTNL	224	228	46.46	7	HHHHH
1HNJ:A	SVADQ	120	124	29.12	10	HHHHH	2B7U:A	QDAVS	139	143	29.02	8	HHHHH
1HX0:A	SGGIE	145	149	53.54	5	TSBCC	1OI7:A	EIGGS	208	212	53.34	7	CSSSS
1I1J:B	PKLAD	4	8	60	5	CCSCS	3ENG:A	DALKP	161	165	60.06	6	GGGHH
1I1K:C	VGDVG	1102	1106	35.86	5	CCSSC	2VA1:F	GVDGV	160	164	35.78	4	SCCSS
1I24:A	ASLVTK	304	309	40.03	7	HHHHHH	3U7I:D	KTVLSA	56	61	40.78	6	HHHHHH
1I2T:A	PRVQA	1019	1023	58.04	8	HHHHH	3CNH:A	AQVRP	153	157	58.88	8	HTCCG
1I7E:A	EHETL	405	409	66.82	7	GGGSH	2YVT:A	LTEHE	143	147	66.18	8	EESSC
1I7E:A	VIVPG	386	390	1.88	9	EEEEC	3KW8:A	GPVIV	175	179	1.1	7	EEEEE
1I7N:B	RQLIT	404	408	35.32	8	HHHHH	3CG7:A	TILQR	190	194	36.2	10	HHHHH
1I9S:A	QVAEE	38	42	76.96	7	TSCGG	1NRG:A	EEAVQ	67	71	77.9	5	HHHHH
1ICF:I	IPAVH	204	208	45.28	7	SCSBC	3GZE:D	HVAPI	245	249	46.2	7	ESSCC
1IFR:A	DPLLT	476	480	76.02	6	SCCEE	2OC5:A	TLLPD	57	61	76.8	4	HHCGG
1IT2:B	KKSNL	258	262	93.72	5	CCSCG	3IMA:D	LNSKK	81	85	94.36	6	GTEEE
1ITX:A	RFSDV	169	173	20.18	7	THHHH	3EQA:A	VDSFR	306	310	20.68	8	HHTTT
1ITX:A	KKYLL	244	248	47.58	9	SCCEE	1YN9:A	LLYKK	78	82	47.44	9	CEEEE
1ITX:A	GDRNK	437	441	47.58	12	GCTTC	3MQD:A	KNRDG	222	226	48.5	11	TTCCS
1ITX:A	VAAGA	314	318	11.54	7	HHHHH	1ZR3:A	AGAAV	255	259	11.88	9	TCEEE
1ITX:A	IAAIV	267	271	24	6	HHHHS	2HY7:A	VIAAI	342	346	23.12	8	HHHHH
1ITX:A	AANTE	259	263	31.3	9	HHTSC	1MZ9:A	ETNAA	39	43	32.02	11	HHHHH
1ITX:A	ASAAG	301	305	46.92	7	HHHTT	1HT6:A	GAASA	238	242	47.32	5	GGGSS
1J9B:A	PSRDE	39	43	75.54	7	CCHHH	1MZG:B	EDRSP	40	44	75.46	7	TTCCS
1JBE:A	FLVVD	8	12	2.46	10	EEEEC	1TOJ:A	DVVLF	184	188	2.34	9	CEEEE
1JBE:A	EKLNK	118	122	77.02	9	HHHHH	3N3M:A	KNLKE	46	50	77.24	8	HHHTC
1JF8:A	AIEAM	48	52	28.5	8	HHHHH	1MXR:B	MAEIA	261	265	27.68	7	HHHHH

1JF8:A	KEILG	25	29	58.18	4	HHHST	3EPZ:B	GLIEK	425	429	58.24	3	STTTT
1JIX:A	VDVND	54	58	54.28	5	SCGGG	1SLU:A	DNVDV	121	125	55.02	5	TTCEE
1JIX:A	AELID	304	308	47.64	8	HHHHH	3DRA:A	DILEA	131	135	47.82	6	HHHHH
1JKG:A	LYMGT	38	42	53.42	8	GEEEE	3NYC:A	TGMYL	1281	1285	52.76	8	HHHHH
1JMV:A	VDLLV	103	107	11.02	9	CCEEE	3TKT:A	VLLDV	391	395	10.14	10	HHHHH
1JNR:D	EPTEE	120	124	88.46	4	CCCHH	2OCZ:A	EETPE	121	125	88.78	5	SCCCT
1JO0:B	LAEIE	35	39	45.52	9	HHHHH	1HXA:A	EIEAL	45	49	44.8	9	HHHHH
1JO8:A	AEDNE	12	16	94.44	4	CSTTB	2B06:A	ENDEA	46	50	93.78	5	CTTSC
1JS3:B	EKLVA	181	185	30.12	9	HHEEE	3MMH:A	AVLKE	41	45	29.82	8	HHHHH
1JT2:A	NERLF	204	208	57.96	5	HHHHC	1EXT:B	FLREN	144	148	57.5	7	EEETT
1JW9:B	ASGKI	219	223	27.04	8	CBSEE	3F9F:B	IKGSA	136	140	28.02	8	EECCT
1JX6:A	QVSDY	77	81	33.18	10	SSCCH	1NZI:A	YDSVQ	52	56	33.92	11	SSEEE
1JY2:P	RTLEG	41	45	60.74	7	HHHHH	3KL2:L	GELTR	104	108	61.5	6	TTSCS
1JZT:B	AVLVS	193	197	5.62	9	SEEEE	1QOP:A	SVLVA	125	129	5.06	8	EEEET
1K3I:A	PSVAS	629	633	26.28	7	BCCCE	3GRE:A	SAVSP	1086	1090	25.34	6	EEEEC
1K3I:A	WGSPV	96	100	45.3	4	CCSCS	3SO0:E	VPSGW	45	49	45.54	3	CCTTC
1K3I:A	IRVTQ	635	639	47.64	7	EEEEC	1LST:A	QTVRI	4	8	47.1	9	SEEEE
1K4I:A	FVVVL	27	31	5.14	9	CEEEE	2WM3:A	LVVVF	6	10	5	10	EEEEE
1K4I:A	MLRGD	193	197	63.6	7	ECCHH	2QG1:A	DGRLM	1763	1767	64.3	7	HTCCC
1K55:A	FSAEA	32	36	68.52	3	HHTTT	3I38:J	AEASF	292	296	67.8	4	HTTTC
1K55:A	IGLET	76	80	20	9	HHHHT	3DRA:A	TELGI	56	60	21	8	HHHHH
1K55:A	FVLCK	41	45	24.2	11	EEEEE	1HL6:B	KCLVF	131	135	24.84	9	HHHHH
1K5N:A	VGYVD	25	29	13.66	9	EEEET	3V8H:A	DVYGV	104	108	13.1	8	SCTHH
1K66:B	IVKPL	119	123	57.84	7	EECCS	3CNE:D	LPKVI	167	171	58.12	5	HHHHH
1K7C:A	VVAEA	203	207	13	9	HHHHH	2W91:A	AEAVV	726	730	12.1	9	EEEEE
1K7C:A	KSVLT	220	224	69.48	7	GGGBS	3CH0:A	TLVSK	221	225	68.98	7	GGCCH
1KD8:B	QLKAK	4	8	72.42	7	HHHHH	3OHE:A	KAKLQ	125	129	73.36	8	HHHHH
1KD8:B	EELKS	10	14	72.8	8	HHHHH	1H6H:A	SKLEE	67	71	71.9	7	HHHHH
1KJQ:B	ALRRV	66	70	53.78	8	HHHHH	2E6F:A	VRRLA	110	114	53.9	8	HHHHH
1KJQ:B	AVILP	324	328	17.76	6	EEECC	3S81:A	PLIVA	197	201	18.28	5	HHHHT
1KJQ:B	PEIDG	356	360	48.14	5	SCEEE	2VRN:A	GDIEP	54	58	48.56	4	TTTEE
1KJQ:B	FGVEL	264	268	3.9	12	EEEEE	3H87:A	LEVGF	42	46	4.12	10	HHHHH
1KMV:A	INLVL	71	75	5.8	9	EEEEE	1EKQ:A	LVLNI	62	66	5.32	8	EEEEC

1KTH:A	LPKDE	7	11	76.2	7	SCCCC	2IIZ:A	EDKPL	217	221	75.54	6	GGSCT
1KWF:A	PASGQ	267	271	73.38	2	CCTTS	3K0B:A	QGSAP	169	173	73.38	4	SCSCS
1KZQ:A	ASSDK	211	215	70.22	5	SSSSS	3S9D:A	KDSSA	70	74	69.84	4	HHHHH
1KZQ:A	PEAED	74	78	72.12	6	TTCCG	2NOG:A	DEAEP	805	809	71.72	5	HTCCC
1KZQ:A	KDGVKVP	169	175	57.686	5	TTEEEES	1IXL:A	PVKVGDK	80	86	58.09	7	CCBTTCE
1L2H:A	TLQLE	79	83	31.2	8	EEEEE	2NNU:A	ELQLT	74	78	32.2	10	HHHHH
1L6P:A	YGKSE	71	75	49.58	8	TEEEE	1TQH:A	ESKGY	40	44	50.06	8	HHTTC
1LBU:A	TWSGTL	5	10	62.35	5	CCCSCB	3AKH:A	LTGSWT	115	120	63.02	5	TTSCCE
1LNI:A	SVLPT	42	46	30.38	6	CCSCC	1A99:D	TPLVS	322	326	29.84	7	GGGSC
1LQV:B	EGSRA	106	110	87.4	4	TSCCC	3GX8:A	ARSGE	124	128	87.26	6	HHHTH
1LSH:B	TSNGL	1385	1389	63.1	6	HHHHC	2QV3:A	LGNST	792	796	62.74	7	CCCCC
1LTZ:A	INPVF	148	152	34.98	7	GSHHH	1TQJ:C	FVPNI	43	47	34.18	5	SSSCB
1LU4:A	IATRA	1061	1065	36.76	8	EECSS	3KL2:L	ARTAI	41	45	35.8	8	GGEEE
1LUC:A	YVNAT	254	258	49.92	6	HHHHH	3QC2:B	TANVY	301	305	50.18	6	CTTCE
1LUC:A	RLVNLG	23	28	21.53	9	HHHHHH	3ING:A	GLNVLR	15	20	20.58	8	HHHHHH
1LYV:A	LAVLA	282	286	0.04	9	EEECS	1NOX:A	ALVAL	179	183	0.72	8	EEEEE
1M0D:A	LYKGS	101	105	66.5	5	SSTTC	3HA4:F	SGKYL	124	128	65.62	4	CHHHH
1M15:A	QLIDD	180	184	53.88	8	HHHHT	2G7S:A	DDILQ	9	13	54.76	9	HHHHH
1M1Q:A	LSDFH	5	9	56.8	9	HHHHH	3LE0:A	HFDSL	140	144	57.64	7	EESCC
1M2D:A	SREVF	27	31	50.92	8	HHHHH	2X9Z:A	FVERS	403	407	50.76	6	EEECC
1M2X:A	NKSFK	177	181	82	7	CEEEE	2X61:A	KFSKN	253	257	82.74	6	HHGGG
1M5Q:2	SNGEV	29	33	46.42	6	TTSCE	2HNF:A	VEGNS	145	149	45.44	8	CCSST
1M8A:B	INAII	34	38	30.12	8	SCEEE	1JYO:D	IIANI	7	11	29.22	8	HHHHH
1MDO:A	GIGTG	323	327	17.42	8	TBCCB	2Y4S:A	GTGIG	533	537	17.26	8	TTTCE
1MDO:A	GGIVV	195	199	0.26	7	CEEEE	3JVD:A	VVIGG	261	265	1.16	9	CEEEE
1MF7:A	THTATG	209	214	8.6	11	BCHHHH	1K3I:A	GTATHT	577	582	9.25	9	CEEETT
1MFM:A	CGVIG	146	150	28.66	9	EEECE	1KVE:A	GIVGC	49	53	28.7	8	HHHHH
1MGT:A	SVEKF	3	7	45.42	8	EEEEE	1NU4:A	FKEVS	59	63	44.98	7	ESSHH
1MJ4:A	EVSSH	11	15	49.44	7	CCTTC	1JS3:B	HSSVE	192	196	49.4	5	CHHHH
1MJU:H	SVYPL	120	124	20.6	5	EEEEE	3KYZ:A	LPYVS	122	126	20.66	7	CCEEE
1MKF:B	LKDVL	51	55	39.16	7	HHHHT	2CLB:A	LVDKL	26	30	39.24	8	HHHHH
1MKY:A	PSSAI	361	365	9.1	7	CHHHH	1JB0:C	IASSP	38	42	9.74	9	EEECT
1MML:A	RLLDQ	80	84	70.48	6	HHHHT	1ZOQ:C	QDLLR	2068	2072	70.52	4	HHHHH

1MUN:A	YFERF	51	55	51.58	9	HHHHH	1MIJ:A	FREFY	1295	1299	50.6	10	HHHHH
1MUN:A	ARNLH	86	90	37.9	9	HHHHH	1V8D:C	HLNRA	114	118	37.1	11	GGTTC
1MZW:B	ASLRA	109	113	61.24	6	HHHHH	3BNY:D	ARLSA	279	283	60.38	8	HHHHH
1MZW:B	AERRE	126	130	79.3	8	HHHHH	1K0D:B	ERREA	265	269	78.38	10	HHHHH
1N62:E	TLAQR	405	409	39.46	10	HHHHH	3JRN:A	RQALT	137	141	40.14	9	HHHHH
1N62:E	LAMKK	445	449	54.64	7	HHHHH	3B5N:H	KKMAL	609	613	53.68	9	HHHHH
1N62:E	SGGVAY	381	386	5.02	10	CEESCS	1ZGK:A	YAVGGS	426	431	4.67	12	EEECCE
1N62:F	CLGAA	131	135	12.84	8	HHTCE	3P04:A	AAGLC	112	116	12.9	8	HHHHH
1N62:F	TAAAA	195	199	0.94	8	SEEEE	3DY0:A	AAAAT	343	347	0.06	10	EEEEE
1N62:F	TGPEG	139	143	53.64	4	EETTE	1K3I:A	GEPGT	420	424	53.08	6	CSTTS
1N62:F	ALIGS	80	84	28.66	8	HHHHC	1IO0:A	SGILA	267	271	27.76	6	HHHHH
1N7H:B	TADVV	129	133	8.16	9	HHHHH	3ACZ:A	VVDAT	178	182	7.22	9	EEECT
1N7O:A	PLLVN	448	452	10.26	7	GGEET	2ATM:A	NVLLP	216	220	10.14	8	SEEBC
1N7O:A	VLIDG	411	415	11.42	9	HHHHH	1NYK:A	GDILV	55	59	11.34	10	TCEEE
1N7O:A	VIEKF	324	328	21.56	7	HHHHH	2OY9:A	FKEIV	48	52	22.06	9	HHHHC
1N7S:A	KVLER	52	56	43.46	12	HHHHH	3G7P:A	RELVK	19	23	43.96	11	HHHHH
1N7S:A	DALQA	68	72	41.3	7	HHHHH	3R2R:A	AQLAD	121	125	41.42	9	HHHHH
1NF8:A	FLVADA	171	176	4.2	10	EEEEEE	3VMK:B	ADAVLF	66	71	3.92	10	SSEEEE
1NFP:A	HYQAA	109	113	37.44	9	HHHHH	1MDO:A	AAQYH	270	274	37.62	7	HHHHH
1NFP:A	ENKKS	205	209	57.24	9	HHHHH	2HZ5:A	SKKNE	73	77	57.78	7	ESSCE
1NKI:A	LTGLN	2	6	36.46	7	EEEEE	1IMJ:A	NLGTL	50	54	36.4	9	HHTHH
1NKI:A	DLLGF	24	28	37.44	5	HTTCC	3RSN:A	FGLLD	160	164	37.26	7	EEESC
1NLQ:D	LLGAE	38	42	35.68	5	EECTT	2A0J:A	EAGLL	28	32	34.74	7	HHHHH
1NLS:A	PQGSSV	165	170	34.83	6	BCSSCE	3Q39:A	VSSGQP	386	391	35.57	7	CCTTSE
1NLS:A	AVVSY	63	67	21.2	10	EEEEC	3IT7:B	YSVVA	49	53	21.7	8	CEEEC
1NOF:A	AVNTN	351	355	15.18	10	EEECS	3PMC:B	NTNVA	87	91	16	10	HHHHH
1NOF:A	SLING	124	128	46.22	7	SSBSC	1HFO:F	GNILS	27	31	46.36	6	HHHHT
1NOF:A	IQLPS	93	97	34.92	8	GGHHH	3KC2:B	SPLQI	75	79	35	6	CGGGE
1NP6:B	SKLDL	100	104	48.76	5	GGCSE	3O5Y:A	LDLKS	96	100	47.96	7	TTCCE
1NQU:D	ALVDR	25	29	43.66	8	HHHHH	1EJ0:A	RDVLA	152	156	43.96	9	HHHEE
1NRG:A	KKNEE	186	190	90.1	8	HHHHH	1ZVD:A	EENKK	575	579	90.6	9	TTTHH
1NUY:A	EKRGK	1108	1112	71.86	7	GGEEE	3OOP:A	KGRKE	97	101	72.28	7	HHHHH
1NYK:A	LLVAR	103	107	9.64	12	EEEEE	1NF8:A	RAVLL	31	35	10.08	11	GEEEE

1NYK:A	PVRVED	179	184	64.1	7	CEEEET	1G60:B	DEVRVP	133	138	64.07	6	GGSCBC
1NZI:A	LCGQR	67	71	51.6	7	ECSEE	3CG6:B	RQGCL	35	39	51.78	7	HHTCE
1O04:G	LDMVL	122	126	20.44	8	HHHHH	3JZ0:A	LVMDL	237	241	19.76	9	HHHHH
1O04:G	GLQAY	481	485	21.48	8	GGGGG	3BB0:A	YAQLG	134	138	20.94	9	HHHHH
1O04:G	DRTYL	98	102	29.42	11	THHHH	2QE9:B	LYTRD	111	115	29.22	12	EEEHH
1O04:G	ALYVA	201	205	7.32	7	HHHHH	3A9S:C	AVYLA	123	127	7.12	7	HHHHH
1O58:B	IVRLD	11	15	33.96	9	EEECT	2I9X:A	DLRVI	33	37	33.74	8	EEEEE
1O6U:C	ENVQD	21	25	81.16	6	HHTTT	1ORJ:D	DQVNE	4041	4045	81.98	6	GGTTS
1O6V:B	LTKLT	294	298	49.46	6	CTTCS	1Z4V:A	TLKTL	212	216	49.32	6	EEEEE
1O6V:B	ISDIT	241	245	46.54	6	CCCCG	3KIK:E	TIDSI	7	11	46.44	5	CHHHH
1O7Z:A	LEIIP	27	31	26.24	6	EEEEC	3ZRX:A	PIIEL	283	287	26.5	8	HHHHH
1O8X:A	KLRRG	13	17	69.56	7	EEEET	3A0S:A	GRRLK	456	460	68.84	8	TSBGG
1O8X:A	EKLRR	12	16	87	9	CEEEE	3A7O:A	RRLKE	83	87	86.5	9	HHHHH
1O8X:A	EKLRR	12	16	87	9	CEEEE	2CWZ:D	RRLKE	128	132	87.78	9	HHHHH
1O9G:A	KIPVA	218	222	53.88	4	CCCCT	3B1F:A	AVPIK	70	74	52.88	5	CSCHH
1O9G:A	RERLTA	135	140	70.53	9	HHHHHH	1JC4:D	ATLRER	104	109	70.73	8	HHHHHT
1OD6:A	PATLR	148	152	53.86	7	HHHHH	1W1O:A	RLTAP	268	272	54.22	6	HHHCB
1OD6:A	EERLA	49	53	56.9	10	HHHHH	1F5V:B	ALREE	55	59	56.88	9	HHHHH
1ODM:A	WPDET	129	133	66.72	5	CCCTT	3FCX:A	TEDPW	110	114	67.12	5	CSTTH
1ODM:A	GKEEN	165	169	68.96	6	TSCTT	3C2U:B	NEEKG	428	432	69.38	5	ETTTE
1OF8:B	RLKKL	90	94	55.6	8	HHHHH	3NRV:D	LKKLR	135	139	54.64	9	HHHHH
1OF8:B	DYSHG	279	283	14.08	10	EEEEE	3CDX:D	GHSYD	151	155	14.88	12	TTTEE
1OF8:B	SFPVG	195	199	7.72	9	SSCEE	1QOP:A	GVPFS	51	55	7.46	7	ECCCS
1OFW:B	DSGAP	7	11	72.64	3	SSSSC	3DXL:A	PAGSD	163	167	71.9	3	CTTST
1OFW:B	RSPLS	231	235	62.66	4	SSCCC	3S9C:A	SLPSR	122	127	61.66	6	BCCSS
1OI0:A	YDENS	101	105	60.5	6	CCTTC	3EGG:D	SNEDY	463	467	60.24	6	CTTTC
1OI7:A	RAFAE	263	267	65.72	7	HHHHH	2AUW:A	EAFAR	51	55	66.72	5	HHHTT
1OIH:D	SLADS	223	227	50.06	7	CHHHH	2G38:D	SDALS	158	162	49.54	6	HHHHH
1OKO:A	CGALV	62	66	0.74	9	TTBEE	1EJD:A	VLAGC	377	381	0.5	7	HHHHH
1OUW:D	PIALT	42	46	6.72	10	EEEEE	3F95:A	TLAIP	761	765	5.76	10	EEEEE
1OUW:D	VNKIN	25	29	44.24	9	SSCCC	3U12:B	NIKNV	56	60	43.46	8	TEEEE
1OUW:D	TITVG	58	62	13.82	10	EEEEE	3AWG:C	GVTIT	443	447	14.38	8	EEEEE
1OZ2:A	ASVTD	365	369	17.6	11	EEEEE	3IVE:A	DTVSA	174	178	17.16	10	HHSCG

1P1J:B	FLRLL	513	517	4.3	10	HHHHH	2F5J:A	LLRLF	263	267	3.44	11	HHHHH
1P1J:B	VLEYD	163	167	62.82	7	CSCHH	3TXS:A	DYELV	49	53	61.92	8	HHHHH
1P1X:A	DIDIA	82	86	44.76	6	CHHHH	1UV7:B	AIDID	142	146	44.66	7	EEEEE
1P1X:A	RIATV	69	73	17.36	10	EEEEE	1GML:A	VTAIR	309	313	17.88	10	CEEEC
1P1X:A	RIATV	69	73	17.36	10	EEEEE	1WKQ:B	VTAIR	56	60	17.56	9	HHHHH
1P1X:A	NDDID	80	84	71.2	8	CSCHH	2H5N:D	DIDDN	100	104	71.48	7	BCCHH
1P28:A	ATEDD	22	26	45.56	10	CCHHH	2P0W:A	DDETA	62	66	44.6	9	TTTEE
1P28:A	PLESRT	35	40	52.93	7	CCCSHH	3H09:B	TRSELP	617	622	53.9	6	TTSCCC
1P5Z:B	ETEWT	149	153	71.94	6	HHHHH	3MSW:A	TWETE	89	93	72.46	7	EEEEE
1P5Z:B	YLEKL	204	208	38.44	7	HHHHH	2NN4:A	LKELY	34	38	37.64	9	HHHHH
1P6O:B	HEVVV	327	331	39.7	8	CEEEE	1CNU:A	VVVEH	36	40	38.92	8	EEEEE
1PMH:X	FNPNNG	54	59	60.4	6	CCTTCS	2PTH:A	GNNPNF	124	129	61.15	8	TSCCCS
1PQH:B	YAIAA	58	62	22.34	9	EEEEE	3D9R:A	AAIAY	15	19	23.22	9	HHHHH
1PV5:A	KLNVRK	24	29	74.72	7	EEEEEE	2IIZ:A	KRVNLK	226	231	73.78	8	HHSCEE
1PWG:A	LPAPD	3	7	54.1	6	CCCCC	1O9G:A	DPAPL	86	90	55.1	6	CHHHH
1Q08:A	IAELQ	97	101	46.64	8	HHHHH	1B1U:A	QLEAI	47	51	46.82	10	HHHHS
1Q33:A	LHKLF	253	257	57.94	6	HHHHT	1L8R:A	FLKHL	218	222	58.14	5	HTTTT
1Q6O:B	LSRMC	72	76	19	10	HHHHH	2W2G:B	CMRSL	623	627	18.1	10	HHTTC
1Q6O:B	GEADI	149	153	53.18	6	CHHHH	1P1J:B	IDAEG	119	123	53.9	4	ECTTS
1Q7L:C	DAFTV	182	186	24.7	8	SSEEE	2VN5:A	VTFAD	32	36	24.4	9	EEEES
1Q7L:C	TARQL	41	45	65.44	7	HHHHH	4DEM:F	LQRAT	289	293	65.74	8	HHHCC
1Q8C:A	AFSEN	63	67	30.7	8	HHHHH	1TH8:A	NESFA	15	19	30.98	10	HHHHH
1QBZ:B	ELLRLT	52	57	42.9	9	HHHHHH	1R8G:A	TLRLLE	311	316	43.38	7	HHHHHH
1QFT:B	ANGAH	10	14	53.16	8	HHGGG	2PTZ:A	HAGNA	156	160	54.02	7	TSSSS
1QFT:B	FTDVIA	108	113	7.68	10	EEEEEE	3BS4:A	AIVDTF	84	89	6.68	12	EEECHH
1QGQ:A	VTWNA	149	153	38.68	7	CBSCC	3EE4:A	ANWTV	222	226	39.2	7	HHHHH
1QOP:A	QGFGI	210	214	12.22	9	EESSC	3EQA:A	IGFGQ	160	164	11.26	7	HHHHH
1QOP:B	FADLL	45	49	31.42	10	HHHHH	3Q64:A	LLDAF	145	149	32.28	8	HHHHH
1QQ5:B	LKRAI	108	112	28.2	12	SEEEE	3PT5:A	IARKL	99	103	28.36	12	HHHHH
1QQF:A	LIKKG	1048	1052	56.46	9	HHHHH	1I7N:B	GKKIL	133	137	56.98	7	TCEET
1QSA:A	SIPGY	510	514	68.82	4	TCCSC	2XXQ:A	YGPIS	236	240	68.16	5	CSCTT
1QSA:A	AQIKQ	11	15	63.54	7	HHHHH	4DNU:A	QKIQA	164	168	63.3	7	EECGG
1QSA:A	MAAVA	217	221	10.44	8	HHHHH	2RI0:B	AVAAM	197	201	9.96	7	HHHHH

1QSA:A	LARYA	419	423	21.38	11	HHHHH	2R78:A	AYRAL	15	19	20.9	10	HHHHH
1QTN:B	RKKLVF	471	476	48.4	7	CSBCCC	3KH0:B	FVLKKR	878	883	48.82	8	EEEEEC
1QTN:B	EVNYE	445	449	55.5	10	HHHHH	1N7S:B	EYNVE	234	238	55.88	10	HHHHH
1QV1:A	ISGIS	141	145	17.32	7	HHSSC	1O9W:A	SIGSI	144	148	17.68	7	EEEEE
1QV1:A	KAYGKI	136	141	39.43	10	HHHHHH	2BJD:B	IKGYAK	42	47	38.43	10	CEEEEE
1QV1:A	SKASD	44	48	47.52	9	HHHHT	3DRF:A	DSAKS	509	513	47.74	8	TSGGG
1QVE:A	TLTLTL	112	117	19.07	10	EEEEEE	3P2U:B	LTLTLT	3	8	18.7	9	EEEEEE
1QVZ:A	TDKKT	148	152	87.96	5	BCTTT	3OOP:A	TKKDT	58	62	87.08	7	HTCCH
1QW2:A	NVIDK	109	113	54.28	6	HHGGG	2GJ4:A	KDIVN	759	763	55.04	6	HHHHH
1R2Q:A	AIAKKL	176	181	49.28	6	HHHHTS	3QWW:A	LKKAIA	405	410	49.82	7	HHHHHH
1R4P:A	SLVSS	134	138	20.92	8	HHHHH	1GXR:B	SSVLS	740	744	20.2	8	SCEEE
1R6D:A	DAGVG	116	120	51.32	6	HTTCC	1RC9:A	GVGAD	103	107	52.14	5	TTEES
1R6D:A	FAAES	83	87	29.4	5	CCSCC	3K6G:E	SEAAF	301	305	29.32	7	HHHHH
1R6D:A	HVSTN	124	128	7.06	10	EEEEG	3IJW:B	NTSVH	179	183	7.54	8	CTTHH
1R6J:A	KDSTG	203	207	83.38	6	CCTTS	2CHH:A	GTSDK	44	48	83.66	6	EEEEE
1R77:A	DKVKL	266	270	44.02	7	GGTBC	3ISX:A	LKVKD	244	248	43.98	7	EECBT
1R7A:B	ALAGK	382	386	30.38	6	HTTCC	3S8G:C	KGALA	6	10	30.48	7	HHHHH
1R7A:B	ALAGK	382	386	30.38	6	HTTCC	1R7L:A	KGALA	90	94	30.94	7	HHHHH
1R7A:B	RSDDL	492	496	49.68	7	EESCT	3C1D:B	LDDSR	69	73	50.26	7	CCHHH
1R8S:A	VGLDAA	23	28	31.17	8	ECSTTS	1L2U:A	AADLGV	88	93	31.08	6	HHHTTC
1R9L:A	ISRYKE	174	179	75	7	HHHHHT	2HPJ:A	EKYRSI	49	54	74.02	6	GGGGEE
1RA0:A	AQTTV	410	414	84.8	4	CCEEE	3O0L:A	VTTQA	48	52	83.98	5	EEEEC
1RA0:A	GGKVI	400	404	55.36	5	TTEEE	2BH4:X	IVKGG	29	33	54.46	5	EECCC
1RA0:A	ALAQK	202	206	55.7	10	HHHHH	2Y27:A	KQALA	391	395	55.18	9	HHHHH
1REW:C	IEEDD	63	67	64.78	8	EEECT	1OXK:E	DDEEI	120	124	64.56	7	TCCCC
1RG8:A	LKKNG	111	115	44.34	7	BCTTS	3A9S:C	GNKKL	281	285	43.82	8	CCHHH
1RHS:A	VDSRA	179	183	12.46	13	EECSC	3ORK:A	ARSDV	195	199	12.8	13	HHHHH
1RHS:A	FLTED	215	219	55.36	8	GBCTT	1PI1:A	DETLF	107	111	56.1	6	CTTTS
1RTT:A	IELAD	39	43	57.96	5	EEECC	2P6W:A	DALEI	128	132	57.36	6	GGHHH
1RTT:A	ELIQQ	169	173	61.6	8	HHHHH	1HW7:A	QQILE	31	35	61.6	6	HHHHT
1RYL:B	EKLIS	145	149	51	9	HHHHH	3GMI:A	SILKE	252	256	51.72	9	HHHHH
1S0P:A	SKFFN	133	137	48.76	9	CTTHH	1X46:A	NFFKS	32	36	48.18	7	HHHHH
1S5P:A	GAGIS	48	52	14.66	9	CTHHH	3OKF:B	SIGAG	21	25	13.92	9	EEETT

1S5P:A	KPRVL	40	44	61.14	6	CCCEE	3US4:A	LVRPK	207	211	61.96	8	CCSBC
1SBY:B	FVILD	33	37	5.56	11	EEEEE	2R4G:A	DLIVF	458	462	5.54	10	HHHHH
1SBY:B	FVILD	33	37	5.56	11	EEEEE	1ZJR:A	DLIVF	25	29	6.42	9	EEEEE
1SC6:A	VVSLH	206	210	10.46	7	EEEEC	2FP8:B	HLSVV	124	128	10.14	7	EEEEE
1SDI:A	LALAG	10	14	1.14	8	HHHHH	3QFH:B	GALAL	401	405	1.56	8	HHHHH
1SFX:B	RLKVL	53	57	38.74	10	HHHHH	2II3:A	LVKLR	226	230	37.88	8	HHHHH
1SG4:C	IGHRA	155	159	25.76	10	HCHHH	1P1M:A	ARHGI	104	108	24.94	10	HTTTE
1SG4:C	TIGHR	154	158	24.06	11	HHCHH	3A35:B	RHGIT	23	27	23.64	9	EEEEE
1SJW:A	RQTEI	3	7	76.48	7	HHHHH	3LLU:A	IETQR	189	193	77.26	7	HHHHH
1SMO:B	KTLAC	65	69	47.22	5	EEEEE	2Y8N:B	CALTK	19	23	47.02	4	ETTTC
1SO7:A	QESQL	256	260	88.26	5	CCCEE	3P8A:B	LQSEQ	124	128	87.4	5	HHTTT
1SPB:P	QTMST	16	20	85.1	5	TTTTC	3F5O:H	TSMTQ	2	6	84.98	6	CCHHH
1SQ9:A	PFADE	157	161	61.54	7	SSSSH	1NML:A	EDAFP	137	141	61.82	5	HHHST
1SU7:A	TGINV	286	290	25.96	8	SCEEE	1X9D:A	VNIGT	377	381	25.56	9	EETTT
1SU7:A	AAVEN	370	374	47.18	8	GHHHH	1T1J:B	NEVAA	26	30	47.42	9	HHHHH
1SU7:A	VKDITG	600	605	43.45	7	THHHHS	2PPL:A	GTIDKV	411	416	43.07	8	CSEEEE
1SU7:A	LAAIN	622	626	15.46	9	HHHHH	3JVD:A	NIAAL	176	180	15.56	9	EEEEE
1SU7:A	ETAAD	615	619	52.16	6	HHHHH	2DVT:C	DAATE	107	111	51.98	6	HHHHH
1SU7:A	THIGV	578	582	6.32	10	EEECS	1FX4:A	VGIHT	1011	1015	6.72	9	EEEEE
1SU7:A	EMENE	275	279	79.6	6	HTHHH	1TU7:A	ENEME	79	83	79.46	8	SSHHH
1SU7:A	VNVAV	254	258	0.4	11	EEEEE	1ST9:A	VAVNV	98	102	1.3	9	EEEEE
1T0B:H	EVVER	82	86	42.44	9	HHHHH	3VMK:B	REVVE	204	208	42.88	10	HHHHH
1T0H:B	VVLDA	316	320	1.7	9	EEEEE	1GKP:E	ADLVV	390	394	2.12	11	CCEEE
1T1V:A	QLVDI	36	40	55.48	8	EEEET	2QE9:B	IDVLQ	87	91	56.14	8	HHHHH
1T6U:A	HVSVL	54	58	14.84	10	HHHHH	1WMZ:D	LVSVH	45	49	15.14	8	ECCCC
1T8K:A	EAEKI	58	62	72.26	5	HHTTC	3DQG:A	IKEAE	541	545	71.68	7	HHHHH
1T92:A	SAPSA	69	73	55.84	6	SCCSS	1QB5:D	ASPAS	82	86	56	6	ECSSS
1TC1:B	LTSSG	86	90	72.1	2	SCSSC	3K1W:B	GSSTL	211	215	72.84	4	TTEEE
1TCA:A	SVSKP	29	33	61.44	5	SCSSE	2I45:I	PKSVS	80	84	61.14	4	CSSCC
1TP6:A	DKTAL	51	55	54.06	7	EHHHH	3LUM:D	LATKD	125	129	53.76	8	ECSBC
1TP6:A	LAGDS	20	24	45.84	8	HHTCC	2RGQ:C	SDGAL	36	40	46.2	6	EEEEE
1TQ5:A	ALRVI	39	43	34.44	6	TEEEE	3EYP:B	IVRLA	446	450	34.42	8	EEEEE
1TQ5:A	AGQGF	50	54	47.06	6	TTCEE	3S6L:D	FGQGA	91	95	46.6	8	ESTTC

1TQH:A	TGPDD	64	68	41.04	6	CCHHH	3H96:B	DDPGT	56	60	41.76	5	SCTTE
1TQJ:C	ANAIV	195	199	2.94	8	CCEEE	1T0B:H	VIANA	213	217	2.04	10	HHHHH
1TU7:A	HCLDK	167	171	79.92	3	TTTTT	3Q7C:A	KDLCH	503	507	80.66	3	GGGCC
1TUK:A	NLRAQ	27	31	64.76	8	HHHHH	2V6X:B	QARLN	222	226	64.28	8	HHHHH
1U02:A	DTYIV	40	44	19.26	8	EEEEE	2X46:A	VIYTD	101	105	19.56	9	EEEEC
1U69:D	TIEKA	152	156	70.96	6	HHHHH	2J6B:A	AKEIT	22	26	70.66	7	EEEEC
1U7G:A	GTANE	221	225	59.02	8	SSSSH	3BKH:A	ENATG	98	102	58.56	6	HHHHC
1U7I:A	YLSLF	23	27	32.16	6	HHHHC	2WOJ:C	FLSLY	246	250	32.08	7	HHHHH
1UAS:A	GRSVM	136	140	58.08	7	TCCHH	1Y63:A	MVSRG	80	84	58.7	7	HTSSS
1UFO:D	GLSVL	12	16	35.08	6	TEEEE	2O1M:B	LVSLG	69	73	35.04	7	HHHHG
1UG6:A	FYDRL	98	102	38.92	12	HHHHH	1SAU:A	LRDYF	57	61	38.4	13	HHHHH
1UG6:A	PFFAT	157	161	11.06	8	CEEEE	2W39:A	TAFFP	228	232	11.72	7	SEEEE
1UNQ:A	APLNN	50	54	63.28	5	SCSEE	1AQ0:B	NNLPA	10	14	63.28	5	SSCCC
1URQ:C	NLKDLG	77	82	48.18	6	HHHHTT	3K0L:B	GLDKLN	107	112	49.13	8	HHHHHH
1US0:A	VDEGL	148	152	50.5	8	HHTTS	2OKG:B	LGEDV	178	182	50.96	9	SCCCG
1US0:A	ASRILL	1	6	67.25	7	CSEEEC	3K0X:A	LLIRSA	78	83	66.83	7	EEEEEC
1US0:A	IDVGY	35	39	34.54	7	HHHTC	2XXQ:A	YGVDI	303	307	33.58	6	HTCCC
1US0:A	VVPSD	130	134	55.24	7	BCBCS	3JXO:A	DSPVV	156	160	55.64	6	TCTTT
1UTG:A	TEKIV	60	64	75.04	6	HHHHH	3I7M:A	VIKET	63	67	74.22	4	HHHTT
1UUQ:A	DMTVV	124	128	28.1	10	TCEEE	3L41:A	VVTMD	721	725	28.08	10	EECHH
1UZ3:A	KDLLG	48	52	40.28	7	HHHHH	3DDC:B	GLLDK	299	303	40.06	8	HHHHH
1UZ3:A	PNSSS	88	92	70	6	SCCSH	2JEN:A	SSSNP	31	35	71	6	EECST
1UZ3:A	GELSKV	52	57	52.75	8	HHHHHH	3U62:A	VKSLEG	99	104	52.03	6	HHHTTT
1UZK:A	YICDC	1509	1513	56.88	7	EEEEC	1RKI:B	CDCIY	22	26	57.44	8	HHHHH
1VCL:A	GDVGT	264	268	22.88	12	SBEEE	1W9H:A	TGVDG	106	110	22.68	10	HCCSE
1VHT:C	DLGIN	24	28	52.34	8	TTTCE	1OTK:B	NIGLD	41	45	52.76	10	HHHHH
1VHU:A	AEVAL	188	192	29.86	8	HHHHH	3K7I:B	LAVEA	169	173	30.38	6	HHHHT
1VK1:A	YGLKE	179	183	54.26	9	ESCHH	3F0D:D	EKLGY	137	141	54.22	8	TTCHH
1VL7:A	FDCTA	89	93	27.2	10	EEEEE	2Y7L:A	ATCDF	77	81	27.32	11	EEEEE
1VPM:C	KPKPV	122	126	60.7	6	CBCCC	1NRG:A	VPKPK	200	204	60.56	5	CCCCT
1VQ3:D	AYEIV	57	61	26.52	9	HHHHH	1NOW:A	VIEYA	274	278	26.82	9	HHHHH
1VQ3:D	GETIE	21	25	45.16	9	HHHHH	3SOY:A	EITEG	46	50	44.38	8	HHHHH
1W0H:A	SKFLN	236	240	45.14	7	HHHHH	1NQU:D	NLFKS	148	152	45.46	8	HHHHH

1W1D:A	EGPHLY	480	485	28.35	9	TTTEEE	1XS1:B	YLHPGE	82	87	28.38	8	EECTTC
1W1O:A	GPMSY	282	286	16.42	9	CSCSE	2W0I:A	YSMPG	257	261	16.1	9	EECCG
1W1O:A	AVLGG	224	228	3.02	8	HHTTC	3GFF:B	GGLVA	146	150	3.46	6	HHHHH
1W23:A	VIVKK	206	210	26.02	11	EEEEH	1Y5M:B	KKVIV	35	39	26.72	9	CEEEE
1W2L:A	EREVA	85	89	65.8	7	HHHHH	3GZR:B	AVERE	141	145	66.78	9	EEEET
1W9A:A	APDDD	94	98	76.92	6	STTSH	2VQP:A	DDDPA	26	30	77.06	4	SSSCC
1W9H:A	GETKI	263	267	42.84	9	HHHHH	3G43:F	IKTEG	1620	1624	42.06	10	HHHHH
1W9H:A	GNGAS	16	20	53.46	7	GGGCE	3S6L:D	SAGNG	20	24	54.14	6	EESTT
1W9H:A	LNVDP	171	175	61.76	8	ECCCG	2YY3:C	PDVNL	17	21	61.5	7	SSSCS
1W9M:A	DIGGI	446	450	45.7	3	EETTE	2FM8:A	IGGID	24	28	45.62	4	BCCCC
1W9M:A	SLAVI	464	468	14.3	8	HHHHH	3GNZ:P	IVALS	120	124	14.04	8	EEEEE
1W9M:A	KLKEV	471	475	69.88	7	HHHHH	2I3D:A	VEKLK	169	173	69.56	6	HHHHT
1W9M:A	FLSPN	521	525	42.08	8	TCCHH	2X61:A	NPSLF	51	55	41.34	6	CGGGH
1WHZ:A	VVVPF	39	43	21.12	7	EEEEC	1I7N:B	FPVVV	265	269	21.48	8	SSEEE
1WL8:A	SGGPSL	50	55	36.33	6	CCCSCT	3KTA:B	LSPGGS	1031	1036	36.48	7	HSTTCE
1WMA:A	EEGAE	244	248	52.5	5	HHHTH	3HRY:A	EAGEE	21	25	53.36	6	TTTCC
1WMA:A	TRGQAA	40	45	69.03	6	HHHHHH	3LR2:B	AAQGRT	56	61	69.53	7	HHTTCC
1WOU:A	SVSGF	8	12	39.16	6	EEESH	1XL3:A	FGSVS	268	272	39.36	5	CCCCH
1WPA:A	KMVGD	512	516	44.08	8	HHHHH	3TKT:A	DGVMK	60	64	43.9	7	HHHHH
1WRI:A	DITFD	12	16	66.24	6	EEEEE	1BKP:B	DFTID	256	260	65.52	6	GCCGG
1WUI:L	YKDWT	284	288	32.82	7	TGGGG	3HTK:C	TWDKY	108	112	32.84	9	HHHHH
1WUI:S	AVIAY	107	111	6.64	8	EEEEE	4A34:T	YAIVA	119	123	5.9	9	SEEEE
1WZ3:B	DKFAN	36	40	48.9	11	SBTHH	3F0H:A	NAFKD	346	350	48.66	9	HHHHH
1WZD:A	RAEVL	79	83	45	10	CHHHH	2E1F:A	LVEAR	1160	1164	45.14	10	HHHHH
1X2I:A	TAPYI	64	68	56.26	7	HSCCC	2RG9:B	IYPAT	248	252	56.3	7	EECCC
1X3K:A	NVGND	80	84	46.5	8	TTTCH	1I7E:A	DNGVN	344	348	45.86	8	ESSBC
1X91:A	VSAAL	101	105	24.84	8	HHHHH	3LLP:A	LAASV	363	367	24.3	8	EEEEE
1X9D:A	LQSFS	624	628	29.5	8	HHHHH	3F95:A	SFSQL	749	753	28.54	9	GTHHH
1X9D:A	LFLLFS	668	673	4.33	8	HHHHHC	3B0D:C	SFLLFL	34	39	4.9	10	HHHHHH
1X9D:A	LGLRK	301	305	67.74	8	TTCHH	3IG2:B	KRLGL	649	653	67.14	6	HHTTC
1XG0:C	AVSIM	130	134	16.94	8	HHHHH	2CPG:A	MISVA	31	35	17.36	8	HHHHH
1XG5:C	DDGHI	142	146	22.5	11	CSCEE	4DGQ:C	IHGDD	222	226	23.14	11	EEETT
1XG5:C	AHIQI	244	248	26.06	8	TTEEE	3Q7H:N	IQIHA	137	141	26.76	10	HHHHH

1XKP:C	IPQLT	122	126	55.82	6	GGGGC	3NVS:A	TLQPI	5	9	55.8	7	EECCC
1XKP:C	LALLE	115	119	25.62	7	HHHHH	1IQ4:B	ELLAL	168	172	26.4	9	HHHHH
1XM8:B	GAKVI	71	75	33.34	8	CCEEE	1Y5M:B	IVKAG	103	107	34.06	7	HHHHH
1XMK:A	EIKEK	298	302	73.86	8	HHHHH	2SAK:A	KEKIE	57	61	74.66	6	HHHHH
1XQO:A	ARREQ	135	139	71.14	8	SCTTS	2WZV:B	QERRA	102	106	71.16	9	HHHHH
1XQO:A	DAVAR	204	208	43.76	8	HHHHH	3MNL:A	RAVAD	40	44	44.56	6	HHHHH
1XQO:A	EARKK	103	107	70.82	12	HHHHH	3RQ5:A	KKRAE	166	170	70.28	10	TSHHH
1XVX:A	LGNQL	43	47	35.68	8	HHHHH	3B93:A	LQNGL	90	94	35.58	8	CSCEE
1XVX:A	QDILR	258	262	37.9	10	HHHHH	1TIF:A	RLIDQ	17	21	38.58	12	EEECT
1Y7R:A	KNVSV	93	97	62.56	8	HHHCC	2ABK:A	VSVNK	45	49	63.06	7	HHHHH
1Y7T:B	AGLEA	65	69	50.98	6	EEEEE	2Y2Z:A	AELGA	55	59	51.2	5	HHHTC
1YBK:B	SLKNL	25	29	44.8	9	HHHHH	2Y7L:A	LNKLS	176	180	44.58	8	GTEEE
1YFQ:A	IEVID	124	128	4.7	11	EEEEC	3I10:A	DIVEI	70	74	4.1	10	SEEEE
1YG9:A	VVVAS	38	42	2.14	9	EEEEC	2NT0:D	SAVVV	455	459	1.78	9	CEEEE
1YMT:A	AQELV	352	356	36.16	8	HHHHH	3FNC:B	VLEQA	61	65	35.18	7	EEEET
1YN9:A	RQNYV	159	163	52.04	7	CHHHH	3H3L:A	VYNQR	191	195	51.12	8	EETTE
1YQH:A	ELDVL	50	54	42.3	8	CHHHH	2GJ4:A	LVDLE	353	357	41.6	8	HHHTS
1YQH:A	AIEVV	27	31	18.1	6	HHHHH	2PRX:B	VVEIA	128	132	18.08	8	EEEEE
1YRB:A	NKILR	86	90	60.98	7	HHHHH	3EWM:B	RLIKN	222	226	61.24	8	EEEET
1YRB:A	DYLTA	186	190	51.7	7	HHHHH	3C8G:D	ATLYD	126	130	51.2	7	HHHHH
1YRB:A	VVFVGT	3	8	3.9	12	EEEECS	1QVZ:A	TGVFVV	24	29	4.87	12	CCBCHH
1YU0:A	NVTIA	209	213	39.66	8	TCBBC	2HFN:F	AITVN	98	102	39.9	7	EEECS
1YU0:A	TSLSG	348	352	60.72	6	GGGCS	3LR2:B	GSLST	88	92	60.24	5	SCHHH
1YU0:A	NEFQA	256	260	44.64	8	HHHHH	1DM1:A	AQFEN	103	107	43.68	6	HHHHH
1YUZ:B	LNLIS	106	110	16.6	8	HHHHH	2VU9:A	SILNL	877	881	16.96	7	EEEEE
1Z2U:A	FKPPK	62	66	76.28	4	SSCCE	2APO:B	KPPKF	432	436	75.9	5	CCCCC
1Z4V:A	SAAPP	253	257	41.5	5	SSSCC	3FZ4:A	PPAAS	36	40	42.5	5	CCCHH
1Z6M:A	ESLQRG	71	76	48.93	8	TTTHHH	2GRC:A	GRQLSE	1478	1483	49.03	7	CCBGGG
1Z9F:A	TTFTI	29	33	16.06	10	EEEEE	1OUW:D	ITFTT	102	106	15.12	11	EEEEE
1ZB1:B	LGSKG	81	85	53.84	5	HGGGG	2V1Q:A	GKSGL	47	51	54.28	7	CCEEE
1ZD8:A	HLSSG	34	38	27.64	9	EEEHH	1YA5:T	GSSLH	37	41	27.58	10	GSEEE
1ZGK:A	IYVLG	566	570	8.62	10	EEEEC	2R2Y:A	GLVYI	45	49	8.38	10	EEEEE
1ZGK:A	LADLQ	355	359	48.06	6	CCCCS	2WY8:Q	QLDAL	65	69	47.62	8	HHHHH

1ZGZ:A	SVDLI	46	50	33.84	7	CCSEE	1JXO:B	ILDVS	627	631	32.86	8	BCCCC
1ZGZ:A	INLPD	54	58	52.22	8	SCCSS	2QEB:A	DPLNI	59	63	51.6	7	HHHHH
1ZHV:A	SNDLE	109	113	73.94	7	GGGHH	2QGS:A	ELDNS	165	169	73.86	7	GSCCC
1ZHV:A	ADLLA	116	120	42.92	9	HHHHH	2A7K:A	ALLDA	183	187	42.9	7	HHHHH
1ZJZ:A	VYLAS	227	231	15.46	9	HHHHS	2J3T:C	SALYV	102	106	15.72	10	HTTHH
1ZJZ:A	LGAYN	152	156	20.14	9	CHHHH	3RH3:B	NYAGL	113	117	19.98	9	HHHHH
1ZL0:A	SILVL	212	216	0.18	10	CEEEE	1TC1:B	LVLIS	45	49	0.06	12	EEEEE
1ZL0:A	VLLSA	119	123	0.24	9	HHHHH	3MD7:A	ASLLV	43	47	0.4	7	CEEEE
1ZR3:A	KGGKE	233	237	74.08	8	HHHHH	1SPB:P	EKGGK	32	36	73.84	8	HTTCE
1ZR3:A	CLALA	294	298	9.24	9	HHHHH	2VA1:F	ALALC	198	202	8.94	8	HHHHH
1ZW0:H	AGDLK	62	66	59.2	7	HHHTC	2X27:X	KLDGA	29	33	59.96	7	EETTE
1ZZK:A	VTIPKD	18	23	55.43	7	EEEETT	1LBU:A	DKPITV	128	133	54.85	8	SCCCCE
2A0M:A	SLKSS	239	243	34.24	8	GBBTT	1PZX:A	SSKLS	88	92	33.54	8	CTTTC
2A2K:A	DKFVI	419	423	45.64	8	EEEEE	1MAI:A	IVFKD	99	103	46.04	7	EEESS
2A40:B	TATST	203	207	46.9	5	BSSSC	3EQA:A	TSTAT	172	176	47.68	5	HHHHH
2A6Q:D	TMMKA	25	29	30.18	8	HHHHH	3EAB:A	AKMMT	179	183	29.76	9	HHHHH
2A6Q:D	LEETA	57	61	32.14	7	HHHHH	2DVT:C	ATEEL	109	113	31.22	9	HHHHH
2ABS:A	FAIGN	16	20	4.3	7	EEECC	3MBR:X	NGIAF	228	232	4.28	8	EEEEE
2ABS:A	ALEVA	178	182	25.06	8	HHHHH	1PZX:A	AVELA	133	137	26	8	HHHHH
2ABS:A	NAIFT	190	194	32.96	10	TCEEE	2X6W:A	TFIAN	512	516	32.44	12	EEESC
2AD7:A	VLTDQ	305	309	16.1	10	EEEEE	1JY2:O	QDTLV	90	94	16.24	10	HHHHH
2AEN:A	SDKWK	134	138	51.32	9	SSSEE	3KUP:C	KWKDS	141	145	51.06	7	EETTS
2AGK:A	NDDAA	65	69	37.52	10	CHHHH	1GA8:A	AADDN	6	10	38.44	10	EECGG
2AJ7:B	VNPFS	62	66	35.36	6	ECGGG	2IP1:A	SFPNV	291	295	35.34	8	GCTTT
2AML:B	AIGYG	217	221	0.24	10	EEECT	3OEN:A	GYGIA	244	248	0.6	11	EECCE
2AML:B	ILADF	19	23	44.18	4	HHHHH	1TOJ:A	FDALI	170	174	45.18	6	HHHHH
2AUA:B	LSCLY	104	108	28.18	7	TTCEE	4ADU:B	YLCSL	176	180	28.82	7	HHHTS
2B06:A	LQSSE	100	104	88.7	6	CCCBT	3U9Q:A	ESSQL	427	431	89.66	5	TSTTH
2B5G:A	YKRRG	140	144	78.42	5	HHTTT	3F8B:B	GRRKY	74	78	78.86	6	SCCEE
2B69:A	RKELEY	308	313	93.05	7	HHHHHH	3EGG:D	YELEKR	483	488	93.67	5	HHHHTT
2BBR:A	PSSVSV	151	156	41.88	7	TTBCHH	2W2G:B	VSVSSP	633	638	42.8	8	EECSSH
2BBR:A	SLSQQ	48	52	58.02	8	HHHHT	1JX6:A	QQSLS	115	119	57.68	8	HHHHH
2BF6:A	YPDKS	320	324	42.82	8	CSTTC	2Q3E:A	SKDPY	398	402	42.04	6	CSSHH

2BFF:A	VGSAA	97	101	2.08	8	HHHHH	3HRQ:B	AASGV	1520	1524	1.88	9	EEEEE
2BH4:X	EADLI	73	77	52.7	6	HHHHH	3PYI:A	ILDAE	136	140	52.32	6	EECSS
2BH4:X	EGFKY	51	55	87.44	3	TTCCC	3A0S:A	YKFGE	487	491	87.12	5	EEETT
2BHU:A	TVPRRL	576	581	80.55	5	CCCCCE	3DDC:B	LRRPVT	241	246	80.7	6	EEEEEC
2BHU:A	LAAFD	166	170	11.7	6	CEECS	2D1P:F	DFAAL	13	17	11.38	6	CHHHH
2BHU:A	GLDYA	244	248	21.22	8	EECTT	1UOC:A	AYDLG	164	168	21.1	8	THHHH
2BJD:B	KLLER	67	71	70.1	8	HHHHH	3RPF:B	RELLK	12	16	70.24	6	HHHHH
2BJI:B	NIERL	2178	2182	30.32	9	HHHHH	2P0L:A	LREIN	37	41	30.1	9	HHHHH
2BJI:B	AAGEK	2074	2078	82.92	5	HTTCC	3HNX:A	KEGAA	67	71	82.28	5	EETTT
2BJI:B	FVAVS	2104	2108	2.54	9	CCEEE	1GQ1:B	SVAVF	469	473	3.38	8	CEEEE
2BJN:B	GSMAD	1	5	63.98	3	CHHHH	1JR7:A	DAMSG	128	132	64.4	4	BTTTT
2BKF:A	AVKQG	71	75	68	5	HHHTT	2GN4:A	GQKVA	306	310	68	6	CEECC
2BKM:B	EITPK	81	85	44.12	10	CCCHH	3BUT:A	KPTIE	99	103	44.54	8	EEEEE
2BKR:A	SDVSL	16	20	32.86	8	HHHHT	1GL4:A	LSVDS	603	607	33.62	8	EEEEE
2BNL:F	ENAAE	60	64	69.42	6	SSTTH	2FFG:B	EAANE	19	23	69.24	6	TTTTS
2BT9:C	IRVYA	61	65	7.6	9	EEEEE	3GE3:E	AYVRI	47	51	8.1	10	SEEEE
2BUE:A	SPSNL	154	158	65.64	6	CTTCH	1ZB1:B	LNSPS	162	166	65.74	8	CCCSS
2BW4:A	FHAAT	99	103	42.68	5	ETTSC	1X46:A	TAAHF	105	109	43.22	6	CHHHH
2BW4:A	LTAAV	233	237	26.88	10	EEEET	3GSZ:B	VAATL	485	489	26.66	10	HHHHH
2BW4:A	GMVPW	140	144	55.2	6	TCHHH	3DXY:A	WPVMG	42	46	54.54	6	HHHHB
2BWQ:A	LEPKW	790	794	63.16	6	SSCEE	2I02:A	WKPEL	104	108	63.12	7	CCGGG
2BZV:A	AEPGK	368	372	68.22	4	CCTTS	2ACA:B	KGPEA	78	82	67.94	5	ECSST
2BZV:A	SAEPG	367	371	54.76	5	ECCTT	2OFZ:A	GPEAS	117	121	55.08	3	STTTT
2C29:F	ENLKS	285	289	82.3	5	TTCCC	2WY8:Q	SKLNE	33	37	81.8	6	HHHHH
2C29:F	KPTIEG	101	106	40.5	8	HHHHHH	2OID:A	GEITPK	362	367	40.78	9	TEECHH
2C2U:A	VQDLS	154	158	31.02	10	HHHHH	3PWT:A	SLDQV	549	553	30.5	10	HHHHH
2C2U:A	GGSPL	120	124	29.1	7	TBCCC	3PWK:A	LPSGG	190	194	29.46	5	SSCTT
2C71:A	ELFTL	662	666	54.6	6	HHHHH	3OLO:A	LTFLE	96	100	54.2	7	EEEEE
2C71:A	GTIIL	624	628	2.56	11	TBEEE	2VDJ:A	LIITG	103	107	1.8	9	EEECC
2CAK:A	DPIVA	103	107	61.52	4	CSCCE	3EOI:B	AVIPD	113	117	61.22	3	TTCCS
2CB9:A	YVLLG	78	82	1.78	9	EEEEE	2RBG:A	GLLVY	89	93	2.52	10	EEEEE
2CF5:A	IIEVV	321	325	50.36	7	CEEEE	2AUA:B	VVEII	188	192	49.76	8	EEEEE
2CHH:A	IQVSV	64	68	20.84	8	EEEEE	3LQW:A	VSVQI	49	53	20.7	6	EEEEC

2CI1:A	RKEAD	98	102	65.44	5	TTHHH	2FP8:B	DAEKR	94	98	65.04	6	CGGGH
2CKW:A	GNVFQ	334	338	40.02	6	SCHHH	3SOK:B	QFVNG	125	129	39.22	7	EETTS
2CLB:A	LKVLL	117	121	28.44	7	HHHHH	4A4Y:A	LLVKL	14	18	29.28	9	EEEEC
2CVD:A	AIVDT	90	94	6.4	7	HHHHH	1QFT:B	TDVIA	109	113	6.52	9	EEEEE
2CWR:A	VKVKL	294	298	30.88	8	EEEEE	2Z0T:A	LKVKV	47	51	31.78	9	EEEEE
2CWS:A	AVFQD	206	210	31.4	9	EEEET	3BQP:B	DQFVA	48	52	32.4	8	HHHHH
2CWS:A	ISSANL	106	111	37.98	10	ECHHHH	1YFQ:A	LNASSI	328	333	37.3	9	CCCCEE
2CWZ:D	EAVFE	9	13	48	8	EEEEE	2ZPU:A	EFVAE	38	42	48.36	9	HHTSE
2CXY:A	KITKV	3	7	57.86	6	BTTHH	3S90:D	VKTIK	1540	1544	57.92	5	HHHHH
2CYJ:A	SLLPE	75	79	48.76	8	EECHH	2V76:B	EPLLS	48	52	49.62	7	EEEEE
2D1S:A	VLTVV	239	243	5.24	7	EEECS	1Y42:X	VVTLV	396	400	5.34	8	HHHHH
2D1S:A	IVDRL	436	440	33.24	9	EEEEG	2B18:A	LRDVI	31	35	33.78	9	HHHHH
2D7V:A	ETEVV	153	157	54.24	8	CSEEE	1GUQ:A	VVETE	213	217	53.94	7	EEECS
2DFB:A	IVENFG	89	94	15.43	11	EEEEES	1ZVD:A	GFNEVI	604	609	16.1	10	HHHHHS
2DVT:C	VNDLG	117	121	70.7	5	HHTTC	3P7X:A	GLDNV	97	101	69.98	6	TCSSC
2DVT:C	LPYMM	223	227	3.72	9	HHHHH	3U52:C	MMYPL	225	229	3.46	9	HHHHH
2DWU:C	LATVV	151	155	13.3	6	HHHHH	3DCZ:A	VVTAL	208	212	13	7	HHHHH
2DWU:C	FFTTG	239	243	6.16	11	EEESS	2EA7:A	GTTFF	118	122	5.86	10	TCEEE
2E10:B	GKGDK	119	123	84.46	5	EETTE	1DEU:A	KDGKG	220	224	85.02	7	HHHTG
2E2R:A	ADREL	272	276	27.1	10	HHHHH	2P7I:B	LERDA	181	185	27.1	11	HHHHH
2E4T:A	ASVVT	87	91	4.56	10	THHHH	1EW0:A	TVVSA	150	154	5.5	10	EEEEE
2E6F:A	LVKFV	186	190	25.52	9	TEEEE	1MGT:A	VFKVL	67	71	24.8	9	HHHHH
2E6F:A	FTRLE	282	286	34.7	9	HHHHH	2HNF:A	ELRTF	117	121	34.62	9	TCEEE
2E6F:A	NLGFD	73	77	35.76	9	BSCHH	3S95:B	DFGLN	558	562	35.52	7	TSSBC
2EA7:A	EKLIK	394	398	67.98	6	HHHHT	3Q7Z:A	KILKE	574	578	68.84	7	HHHHH
2EB4:B	HTLIA	7	11	21.54	9	HHHHH	2J3W:B	AILTH	149	153	21.3	8	HHHHH
2ECU:A	LSLEP	38	42	47.66	5	EETTT	3BHD:A	PELSL	43	47	48.24	6	TTCHH
2ECU:A	ALARF	8	12	20.34	9	HHHTS	2DTJ:B	FRALA	34	38	19.86	8	HHHHH
2ECU:A	GEEER	24	28	98.12	6	TTEEE	2DYJ:B	REEEG	53	57	98.16	5	SCHHH
2EIX:A	ETLGY	269	273	35.78	8	HHHTC	1O9W:A	YGLTE	74	78	35.54	8	EESSS
2END:A	NLTLV	5	9	28.94	7	CCSCG	1CKN:B	VLTLN	18	22	28.64	8	EEEET
2EQ7:C	ERLMQ	136	140	52.04	10	HHHHH	1MZ9:A	QMLRE	32	36	52.44	8	HHHHH
2EWR:A	LKLGR	138	142	78.66	6	HHHTC	1EJX:A	RGLKL	3026	3030	77.98	8	TTCCB

2EX4:B	GAGIG	70	74	7.1	6	TCTTT	1G8M:A	GIGAG	444	448	6.42	8	EEECS
2F0C:A	GDETIA	40	45	48.75	8	SCEEEC	1AOH:A	AITEDG	94	99	48.4	9	CBCSSE
2F0C:A	PTASS	147	151	54.32	5	CCBSS	1R8H:F	SSATP	281	285	54.14	4	CCEEE
2F5T:X	RGVTV	157	161	19.42	11	TTCEE	1YIS:A	VTVGR	149	153	18.9	9	EEEEE
2F5V:A	ELVGA	64	68	21.82	9	HHHHT	1Z9T:A	AGVLE	132	136	21.32	9	HTHHH
2F5V:A	MSLAI	599	603	0.36	8	HHHHH	3QT9:A	IALSM	359	363	0.78	6	HHHHH
2F6M:B	TVDKL	70	74	44.36	9	HHHHH	3SWN:A	LKDVT	44	48	45.06	9	EEEEE
2F6U:B	MISGT	2035	2039	23.18	9	EECCC	1BM8:A	TGSIM	22	26	23.26	11	SCEEE
2F6U:B	ADTIIV	2209	2214	5.42	10	CSEEEE	3B1F:A	VIITDA	92	97	6.03	12	CEEECC
2F9H:A	GDTNY	61	65	44.44	8	TTEEE	3GDC:C	YNTDG	183	187	44.3	7	ECCSC
2FAU:A	RYFLK	129	133	15.76	10	EEEEE	3F65:E	KLFYR	108	112	16.42	10	EEEEE
2FB0:A	LKEEG	30	34	95.84	6	TTSTT	2FBI:A	GEEKL	121	125	95.08	6	CHHHH
2FB5:B	ESSSF	75	79	59.22	7	SCHHH	1W98:B	FSSSE	292	296	59.52	7	TSCHH
2FB6:A	NSKNR	29	33	78.94	6	HHHHH	1IFR:A	RNKSN	455	459	78.28	8	EECSS
2FE5:A	SIAGG	239	243	17.06	8	EEEEC	3B9W:A	GGAIS	269	273	16.34	9	HHHHH
2FE5:A	VRHEE	289	293	76.62	7	CBHHH	1S5P:A	EEHRV	68	72	76.76	7	TTEEH
2FG1:A	VQFVN	65	69	45.58	7	EEEEE	2CKW:A	NVFQV	335	339	45.32	5	CHHHH
2FL4:A	TNGER	135	139	63.92	7	TTSCE	2RDG:A	REGNT	31	35	64.74	6	EETTE
2FLH:B	ESSHE	76	80	63.06	6	TTTTE	2RDE:B	EHSSE	37	41	62.22	5	CTTCE
2FLH:B	RRLER	144	148	67.7	11	HHHHH	2BZW:B	RELRR	107	111	68.02	10	HHHHH
2FM8:A	IVLDL	33	37	11	8	EEEEE	2Q0S:A	LDLVI	84	88	11.36	8	CSEEE
2FU2:A	IFDLS	69	73	31.94	7	HHHHH	3OAB:C	SLDFI	167	171	31.78	6	CHHHH
2FUE:A	CLDSL	201	205	27.2	7	HHHHH	2FEA:B	LSDLC	180	184	28.2	8	TCSEE
2FUF:A	TTKEK	163	167	65.84	8	ECHHH	2IFT:A	KEKTT	179	183	65.56	8	EEEEE
2FZV:A	LQFFGAE	61	67	34.386	7	HHHTTCE	3JVD:A	EAGFFQL	159	165	34.19	6	HHHHHHH
2G0C:A	DNASY	443	447	51.14	8	SSCEE	3SE2:A	YSAND	1640	1644	50.4	7	HHHST
2G1U:A	ISMNA	89	93	14.96	10	HHHHH	3EJ9:F	ANMSI	48	52	15.5	8	GGEES
2G1U:A	NLASS	24	28	47.08	10	HHHHH	1WW7:A	SSALN	33	37	47.76	8	ESCCC
2G7S:A	AATFGA	176	181	43.63	5	TTHHHH	1GP0:A	AGFTAA	1536	1541	43.3	6	SCEEEE
2G7S:A	ADDIL	8	12	30.38	8	HHHHH	3U3L:C	LIDDA	121	125	29.5	8	HHHHH
2G7S:A	RANAE	153	157	55.1	9	HHHHH	3C8Z:B	EANAR	335	339	54.3	9	HHHHH
2G7S:A	FPSKS	47	51	45.92	8	CSSHH	1FX4:A	SKSPF	943	947	46.62	7	ESCHH
2G7S:A	RGIAQ	139	143	64.56	7	HHHHT	3RMI:A	QAIGR	36	40	64.36	9	HHHHH

2G9Z:B	LKKGV	238	242	49.38	9	ECSEE	3SOK:B	VGKKL	117	121	49.78	7	TTCEE
2GB4:B	EQGHQ	38	42	72.08	8	TTCCH	3C8L:B	QHGQE	16	20	71.24	9	SSSSC
2GB4:B	LVAVLS	176	181	10.07	7	EEEEEE	2PKF:B	SLVAVL	281	286	11.03	8	HHHHHH
2GB4:B	HKAWG	222	226	53.14	6	HHHTT	2E2R:A	GWAKH	281	285	53.88	6	HHHTT
2GB4:B	LLTEK	236	240	61.1	6	EEEEC	3O0A:B	KETLL	253	257	60.46	5	HHHHH
2GDQ:B	YDAAA	195	199	50.4	5	CCHHH	2RB5:A	AAADY	237	241	51.4	6	HHSSE
2GDQ:B	LIRII	36	40	16.98	10	EEEEE	3PKZ:G	IIRIL	113	117	16.4	10	HHHHH
2GEF:A	LALASS	697	702	14.63	6	HHHHHH	2NTX:A	SSALAL	35	40	14.62	6	CHHHHH
2GEF:A	AVAGQ	687	691	38.74	8	CEESS	1XS1:B	QGAVA	183	187	37.94	6	CSCCC
2GFQ:C	RGETR	253	257	63.54	7	CHHHH	2YG2:A	RTEGR	112	116	64.42	6	EETTE
2GGC:A	GMTFT	198	202	3.88	8	TCEEE	1XU1:D	TFTMG	166	170	3.34	8	SSEEE
2GGC:A	DIVNI	92	96	4.34	10	CEEEE	1ZEE:A	INVID	242	246	3.6	10	HHHHH
2GGC:A	VIKDG	100	104	51.82	5	EEETT	2E10:B	GDKIV	121	125	51.66	7	TTEEE
2GGC:A	VAGRL	16	20	36.56	11	HHHHH	3H87:C	LRGAV	55	59	37.3	9	HHHHS
2GHC:X	VDKYA	221	225	33.26	8	HHHHH	451C:A	AYKDV	26	30	33.24	7	CHHHH
2GHC:X	KAVEK	14	18	65.66	7	HHHHH	1TC1:B	KEVAK	23	27	64.7	9	HHHHH
2GHC:X	GSDHL	137	141	47.94	7	CHHHH	3I1A:B	LHDSG	1069	1073	47.3	5	HHHTT
2GIB:A	QIAQF	304	308	39.08	6	HHHTT	3D7R:B	FQAIQ	127	131	38.08	7	HHHHH
2GIX:D	RDLAE	359	363	66.14	8	HHHHH	3SIQ:D	EALDR	123	127	66.96	7	HHHHT
2GJ4:A	LITAI	622	626	7.58	8	HHHHH	3CG7:A	IATIL	188	192	8.2	9	HHHHH
2GJ4:A	IAERI	503	507	44.54	7	HHHHH	2H9D:A	IREAI	13	17	44.16	6	HHHHH
2GJ4:A	VVAAT	299	303	0.68	10	HHHHH	3FOT:A	TAAVV	359	363	1.44	8	EEEEE
2GKG:A	LEGRG	22	26	58.58	5	HHHHT	3Q62:A	GRGEL	17	21	57.82	6	HTTCS
2GKM:B	YTGAP	72	76	36.88	7	CCSCC	3QO4:A	PAGTY	71	75	37.1	6	CTTEE
2GMW:B	MLLSA	136	140	22.88	7	HHHHH	2XTY:B	ASLLM	201	205	22.16	8	HHHHH
2GMW:B	FTEAQ	83	87	50.02	6	SCHHH	2UY2:A	QAETF	121	125	49.14	8	HHHHH
2GMY:A	SEQWI	65	69	32	8	CHHHH	1MAI:A	IWQES	51	55	31.38	6	EEECC
2GUK:B	TNLFF	62	66	9.22	10	EEEEE	2PPL:A	FFLNT	343	347	9.12	10	EEECC
2GWM:A	KETLL	424	428	35.76	10	HHHHH	2FUR:A	LLTEK	125	129	36.52	11	HHHHH
2GWM:A	RLSDD	568	572	72.42	7	CBCCS	2Y6X:A	DDSLR	78	82	72.1	6	CHHHH
2GZ4:A	AEAQK	157	161	62.34	8	HHHHH	1S35:A	KQAEA	1178	1182	61.4	8	HHHHH
2H29:A	KEQHK	93	97	91.2	6	HHHST	1R8G:A	KHQEK	271	275	90.4	5	CCCGG
2H3L:B	TVELI	1401	1405	36.98	9	EEEEE	1V96:A	ILEVT	25	29	36.32	7	HHHHH

2H7Z:A	RPDEN	37	41	89.34	7	EECTT	2I7H:D	NEDPR	98	102	90.34	5	ECCSS
2H8G:B	EGAAV	202	206	3.08	8	SHHHH	1NQU:D	VAAGE	61	65	3.62	6	HHHHH
2HC1:A	FIALD	1914	1918	0.9	10	HHHHH	3U9Q:A	DLAIF	383	387	0.36	11	HHHHH
2HCR:B	ISRIN	258	262	51.78	7	HHHHH	3NA3:A	NIRSI	215	219	52	8	HHHHH
2HDS:B	TPAVR	305	309	53.84	6	ECCCS	2HY7:A	RVAPT	174	178	53.46	6	HHGGG
2HDS:B	GIVML	335	339	0.14	8	EEEEE	2R9G:P	LMVIG	279	283	0.82	7	HHHHH
2HDS:B	SDNKI	287	291	69.86	8	TSHHH	2WX3:A	IKNDS	555	559	69.4	7	HHHCH
2HDS:B	ELTAK	95	99	72.96	7	TCCCG	1XVX:A	KATLE	159	163	72.6	6	HHHHH
2HDS:B	TFTGV	68	72	0.52	9	HHHHH	2BHU:A	VGTFT	134	138	1.52	7	HHHHS
2HEK:B	RLIDS	21	25	33.7	7	HHHTS	1XRX:D	SDILR	26	30	33.8	9	HHHHH
2HEU:A	AKAGV	88	92	49.96	6	HHTTC	3IRB:A	VGAKA	126	130	50.16	4	TTCBC
2HQL:F	TISIE	63	67	22.9	10	EEEEE	2QEA:A	EISIT	124	128	22.7	9	EEEEE
2HTS:A	KSNEKFI	209	215	71.871	5	GGGTTTC	3MDP:A	IFKENSK	39	45	72.37	6	EECTTSB
2HUJ:A	ELQLRQ	69	74	76.3	7	HHHHHH	2Y3Y:A	QRLQLE	121	126	75.3	7	HHHHHH
2HW4:A	LIPDV	8	12	53.46	8	GSCSE	3BJQ:A	VDPIL	12	16	54.46	6	BCHHH
2HYK:A	VWSDE	10	14	45.7	9	EEEEC	3H7H:A	EDSWV	67	71	44.78	8	GGCHH
2HZY:A	AEDSD	7	11	78.04	8	CTTCS	2QTQ:D	DSDEA	120	124	77.32	8	HCCHH
2HZY:A	KGTKA	368	372	83.58	4	TTTSC	3V8H:A	AKTGK	288	292	84.52	3	HHHCC
2I02:A	DEPDI	117	121	69.38	6	GCTTE	1SVI:A	IDPED	165	169	69.44	7	CCTTS
2I3D:A	PEIEG	122	126	54.78	6	TTEEE	1N67:A	GEIEP	552	556	54.22	8	TCCCS
2I5I:B	SIETK	160	164	72.98	4	SGGGG	3K1S:I	KTEIS	68	72	73.58	6	CCCCC
2I5V:O	GVLEG	74	78	36.66	6	EEEEE	1VQS:D	GELVG	35	39	36.64	5	CCEEE
2I6J:A	GGIGRT	98	103	19.95	11	SSSHHH	1TXN:A	TRGIGG	205	210	20.37	9	CCCCEE
2I7D:A	LADKV	57	61	31.66	8	HHHHH	2WJ6:D	VKDAL	80	84	30.74	9	HHHHH
2IA1:A	VPQDE	65	69	40.02	7	SCHHH	3SIQ:D	EDQPV	93	97	39.58	8	SCCHH
2IA1:A	IRDVI	59	63	51.72	7	GGGTC	1O50:A	IVDRI	26	30	51.46	9	HHHHH
2IA7:A	DFGCG	53	57	30.4	6	TCSCG	1LUC:A	GCGFD	33	37	29.96	6	GGTCS
2IA7:A	ARGER	44	48	81.64	5	CTTSS	1YRE:A	REGRA	64	68	82.22	5	HTTSE
2IBP:B	IDLYL	204	208	8.6	9	HHHHH	1GA8:A	LYLDI	100	104	9.36	9	EEECS
2IBP:B	KELAY	144	148	46.96	9	HHHHH	3GMI:A	YALEK	68	72	47.18	7	HHHHH
2IG6:A	LYFTK	110	114	40.18	9	EEEEE	3KCU:C	KTFYL	33	37	39.64	8	HHHHH
2IIZ:A	EDKPL	217	221	75.54	6	GGSCT	2OCZ:A	LPKDE	36	40	74.64	6	SCGGG
2IJ2:A	SLYAK	332	336	29.22	9	EEEES	3NPF:B	KAYLS	154	158	29.26	8	EEEEE

2IJ2:A	GEYPL	343	347	55.1	5	TTEEE	3CJS:A	LPYEG	45	49	55.3	3	CSSCC
2IMJ:D	AFLTR	63	67	52.68	8	HHHHH	2UY2:A	RTLFA	172	176	51.74	7	HHHHT
2IMQ:X	SLLTS	66	70	58.96	7	HHHHH	1PA2:A	STLLS	198	202	58.74	6	HHHHH
2ISB:A	AIDAHG	169	174	20.65	8	EECTTS	3KZ9:D	GHADIA	38	43	20.5	8	CHHHHH
2IUW:A	IASLS	204	208	1.92	10	EEEEE	1H16:A	SLSAI	535	539	1.8	9	HHHHH
2J43:B	LPAGE	17	21	63.16	4	CCTTC	3O5U:B	EGAPL	81	85	63.2	5	SCCCB
2J9O:D	LSPRS	185	189	60.9	7	SCHHH	1ZJR:A	SRPSL	179	183	61.56	6	SSCSS
2J9O:D	QQFQE	142	146	65.94	7	HHHHH	3CG7:A	EQFQQ	90	94	65	6	HHHHH
2JC9:A	TFLVI	368	372	0.3	11	EEEEC	2XTM:B	IVLFT	142	146	0.88	11	EEEEE
2JE6:A	LEELKK	266	271	69.32	7	HHHHHH	1A92:B	KKLEEL	25	30	69.98	8	HHHHHH
2JE6:A	LTDYR	34	38	35.66	8	TTCCC	2APL:A	RYDTL	74	78	35.58	7	HHHHH
2JER:H	IKGSF	279	283	52.98	5	CCTTC	3AAY:A	FSGKI	169	173	53.38	7	HHTSC
2JER:H	EDGDI	295	299	78.82	5	CTTCE	1ZPS:B	IDGDE	114	118	78	3	EETTE
2JFR:A	ALGIG	158	162	21.7	5	CBSSS	1ZJZ:A	GIGLA	17	21	22.16	5	HHHHH
2JHF:A	LLLSG	307	311	10.88	8	HHHTT	1S9U:A	GSLLL	129	133	11.66	6	HHHHH
2JJS:C	TKSVE	7	11	63.08	7	CSEEE	2Q5R:C	EVSKT	30	34	62.3	7	SCEEE
2LIS:A	AEIGR	90	94	50.1	6	HHHHH	2NX4:A	RGIEA	25	29	50.24	6	HCTTT
2NL9:A	SLEII	178	182	25.86	9	HHHHH	3ZRX:A	IIELS	284	288	25.4	11	HHHHH
2NQW:A	LSGLF	46	50	8.18	7	HHHHH	2Q3P:A	FLGSL	93	97	7.65	9	HHTTE
2NQW:A	LGDGS	9	13	57.56	2	CTTSC	1BJA:A	SGDGL	65	69	57.5	4	ETTEE
2NR7:A	KLLLP	5	9	63.48	6	HHHHH	2AXI:A	PLLLK	32	36	62.68	4	HHHHH
2NSZ:A	HSYSV	421	425	63.14	8	THHHH	3MEA:A	VSYSH	181	185	63.74	6	EEEET
2NT0:D	RSSKD	463	467	85.74	5	CSSSC	3D6M:A	DKSSR	157	161	84.8	3	CTTTC
2NUH:A	IQLLI	56	60	17.76	10	EEEEE	3F0H:A	ILLQI	240	244	17.26	10	HHHHH
2NW2:A	LIVHP	112	116	20.26	9	EEEEC	1KCF:B	PHVIL	111	115	19.74	10	CSEEE
2NWF:A	DEAGE	117	121	63.02	8	STTSC	3EAT:X	EGAED	95	99	63.82	7	TTCCC
2NXV:B	ALEEA	101	105	74.88	7	HHHHH	1MDO:A	AEELA	20	24	75.6	7	HHHHH
2NXV:B	TPDKAE	43	48	56.97	8	CTTTEE	2Z4U:A	EAKDPT	26	31	56.35	8	HHSCSS
2O1M:B	TITVGT	17	22	21.25	9	EEEEEE	3AWG:C	TGVTIT	442	447	21.2	9	EEEEEE
2O90:A	DIVFI	2	6	57.3	6	EEEEE	2IU5:A	IFVID	96	100	57.08	6	HHHHC
2O90:A	VERVAEEVA	72	80	36.589	7	HHHHHHHHH	2OIK:D	AVEEAVREV	70	78	35.96	9	HHHHHHHHH
2O9A:A	VTKLL	80	84	39.66	8	HHHHH	3FYQ:A	LLKTV	2137	2141	40.6	8	HHHHH
2OA5:A	SRALG	57	61	37.92	8	HHHHH	3EAT:X	GLARS	48	52	38.08	9	HHHHH

2OB0:C	DKFYKD	33	38	84.37	5	HHHHHH	2P8J:A	DKYFKD	166	171	84.93	6	HHTTTT
2OB3:B	MAFIP	325	329	6.76	7	GGHHH	2IBP:B	PIFAM	365	369	6.96	7	HHHHH
2OC5:A	LEANRE	180	185	64.4	8	HHHHHH	3HE4:B	ERNAEL	15	20	63.82	10	HHHHHH
2OCT:A	NKSLT	77	81	69.04	8	CCCEE	1U7L:A	TLSKN	212	216	69.36	7	GSSTT
2OFZ:A	TLPKG	167	171	62.96	5	CCCTT	1OZ2:A	GKPLT	407	411	63.74	6	TCCEE
2OIZ:A	WVPGG	323	327	8.66	5	CEECC	3TU8:A	GGPVW	101	105	7.8	5	TSCEE
2OIZ:A	DGLFR	182	186	19.82	9	GGGEE	1KLX:A	RFLGD	61	65	20.34	9	HHHHH
2OIZ:A	SIGIV	204	208	1.2	11	EEEEE	2AML:B	VIGIS	89	93	2.12	9	EEEEC
2OIZ:H	CTTSV	171	175	2.26	14	EECCE	3U7Q:D	VSTTC	149	153	2.6	13	EEECH
2OIZ:H	VNSCD	72	76	53.92	6	HTSTT	2Y7L:A	DCSNV	208	212	53.34	5	EEEEE
2OJ6:A	AFHND	386	390	45.16	6	TTTTC	2GIA:D	DNHFA	147	151	45.92	7	ETHHH
2OKG:B	LKKID	258	262	84.5	6	HHHHH	2CLB:A	DIKKL	22	26	84.76	5	CHHHH
2OKQ:B	WASDV	42	46	34.52	7	EEEEC	2D0O:D	VDSAW	47	51	35.04	7	HHHHH
2OKT:A	QDFTQ	179	183	73.54	6	GGHHH	1LIH:A	QTFDQ	178	182	74.38	5	TTSCS
2OPE:C	SLVGV	99	103	4.72	10	EEEEE	3O5Y:A	VGVLS	113	117	4.04	10	EEEEE
2ORD:B	VIKEL	282	286	27.24	8	HHHHH	1KQ3:A	LEKIV	228	232	27.2	9	HHHHH
2ORD:B	ARKLC	197	201	38.78	11	HHHHH	1Z3E:B	CLKRA	265	269	38.32	12	HHHHT
2ORW:B	VVKDL	95	99	15.78	9	HHHHH	3BZN:A	LDKVV	188	192	15.84	10	CCEEE
2OS0:A	GDVGL	57	61	22.66	8	CCSEE	3CH0:A	LGVDG	255	259	22.28	9	HTCSE
2OS0:A	EEVPV	20	24	77.32	5	BCCCS	3FMB:B	VPVEE	14	18	77.74	6	SCHHH
2OS0:A	AEEVP	19	23	64.46	6	CBCCC	1QVY:A	PVEEA	161	165	64.42	5	CCEEC
2OS5:A	AESMNK	27	32	36.08	11	HHHHTC	3PIK:A	KNMSEA	124	129	36.27	10	HHHHHH
2OSX:A	RAVAD	213	217	43.58	8	HHHHH	1XQO:A	DAVAR	204	208	43.76	8	HHHHH
2OUI:D	LPRDI	136	140	85.4	4	CCTTS	2ARC:A	IDRPL	36	40	84.72	4	EEETT
2OV0:A	ADGAI	17	21	71.14	4	CTTCE	3BCW:A	IAGDA	29	33	71.48	4	EEECC
2OVG:A	SINQA	29	33	58	7	HHHHH	1DYT:A	AQNIS	90	94	58.7	6	CSSST
2OXL:A	EEESA	28	32	58.26	8	HHHHH	1M1E:B	ASEEE	35	39	57.44	9	HHHHH
2P0B:A	INPNN	43	47	72.38	5	BCTTT	3MW4:A	NNPNI	243	247	72.86	5	TCTTE
2P0L:A	REINR	38	42	57.7	9	HHHHH	3EVZ:A	RNIER	122	126	56.96	9	HHHHH
2P0N:B	IAENG	39	43	75.7	6	HHHHC	3BB9:F	GNEAI	45	49	76.62	7	TCHHH
2P0N:B	AEPIE	12	16	79.78	3	CCHHH	3FH3:B	EIPEA	138	142	79.76	4	GSCGG
2P13:B	IGKVR	507	511	37.98	10	BCEEE	1U58:A	RVKGI	226	230	37.74	9	EEEEG
2P14:A	LGKSI	109	113	34.5	10	EESSC	2Y7L:A	ISKGL	216	220	34.34	8	EEESB

2P1O:A	LPNVT	44	48	48.08	7	CTTCC	3FDW:B	TVNPL	419	423	48.04	7	CSSCE
2P26:A	GKLIY	504	508	54.92	7	TCEEE	2V5T:A	YILKG	257	261	54.58	8	EEEET
2P2S:A	EQLIT	58	62	54.38	6	HHHHT	1I5N:B	TILQE	60	64	54.66	6	HHHHH
2P2S:A	RERTE	305	309	95.74	9	HHCCC	3QBM:A	ETRER	6	10	95.64	9	HHHHH
2P39:A	SLGRA	137	141	73.26	3	CSSSC	3PWT:A	ARGLS	188	192	73.28	5	CTTCC
2P4F:A	LITSL	196	200	24.68	9	EEEET	1M1F:B	LSTIL	105	109	24.04	7	HHGGG
2P7O:A	GDSLQ	59	63	62.12	6	CSSCC	3FO3:B	QLSDG	310	314	62.9	7	CTTTC
2P7O:A	NAEEI	27	31	68.64	8	CCEEC	1UWW:B	IEEAN	44	48	67.84	6	EEEET
2P8G:A	YEIYI	19	23	8.16	11	EEEEE	2F6U:B	IYIEY	2164	2168	7.64	11	EEEEC
2P8I:A	LREQI	29	33	40.92	9	HHHHH	3R84:W	IQERL	6	10	41.78	7	HHHHH
2P8J:A	INFLT	125	129	2.26	11	EEEEE	1ZB1:B	TLFNI	120	124	1.36	9	HHHHH
2P9W:A	KSANI	289	293	60.94	9	SEEEE	2Y1B:A	INASK	94	98	61.8	9	HHHHH
2P9W:A	ARVSA	184	188	20.26	10	EEECT	3ETQ:A	ASVRA	593	597	19.4	9	SEEEE
2P9X:A	ILTKT	7	11	31.6	9	HHHHH	1AX8:A	TKTLI	10	14	32.16	10	HHHHH
2P9X:A	GRDIL	4	8	41.66	9	HHHHH	3ME5:A	LIDRG	375	379	40.74	7	EECCC
2PC1:A	NRNDI	41	45	75.06	7	CHHHH	2C6U:A	IDNRN	140	144	75.82	8	CCSHH
2PC1:A	NEIDQ	9	13	67.22	6	GGHHH	3RQT:A	QDIEN	214	218	68.18	5	HHHHH
2PC1:A	AAVIDG	67	72	23.83	8	EEEEEE	3FDR:A	GDIVAA	31	36	24.75	7	TCEEEE
2PFI:B	ESQIL	586	590	39.14	9	TTCBE	3K1S:I	LIQSE	59	63	38.44	10	HHHHH
2PGO:B	AMGSA	253	257	4.7	8	EEEEC	3S95:B	ASGMA	445	449	3.94	9	HHHHH
2PGO:B	EYGTE	16	20	34.22	12	HTTCC	3Q1P:B	ETGYE	116	120	33.94	10	HHCEE
2PGO:B	FRAAW	356	360	41.14	10	HHHHH	1DM9:B	WAARF	16	20	41	8	HHTTS
2PKF:B	KGVDL	227	231	41.24	8	GCEEE	1NC7:C	LDVGK	96	100	41.4	9	EEEET
2PKF:B	KGVDL	227	231	41.24	8	GCEEE	1G5H:C	LDVGK	359	363	41.96	9	EEECS
2PKF:B	QTDPT	249	253	67.82	4	CCCCT	2B5I:B	TPDTQ	173	177	66.9	6	CTTCE
2PKH:H	ERALA	125	129	72.3	5	HHHHH	3E1I:A	ALARE	168	172	71.6	4	CCCCC
2PN0:C	EKISI	87	91	30.24	9	CEEET	3MW4:A	ISIKE	157	161	30.12	9	EEEEC
2PN0:C	ILAPV	91	95	7.44	6	TTSTT	1K0D:B	VPALI	165	169	7.74	5	SCEEE
2PNL:J	NRLES	522	526	76.18	6	HHHHT	4ADU:B	SELRN	53	57	75.24	5	TTTSS
2PQ8:A	KLSYR	358	362	62.46	9	HHHHH	3PRO:D	RYSLK	85	89	61.56	8	SSCHH
2PR5:B	DNIRT	76	80	69.86	8	HHHHH	1AX8:A	TRIND	19	23	69.56	8	HHHHH
2PR5:B	EPVTV	86	90	62.78	6	CCEEE	1G8M:A	VTVPE	116	120	62.98	4	CCHHH
2PRX:B	IASLI	61	65	6.24	8	HHHHH	3KG4:A	ILSAI	39	43	6.58	6	HHHHH

2PU3:A	GAIAR	168	172	3.82	10	HHHHH	1JT2:A	RAIAG	166	170	2.84	12	EEEEE
2PV2:D	RIQEL	235	239	34.24	6	EGGGS	3CEG:A	LEQIR	4713	4717	34.64	6	HHHHH
2PV2:D	DQVNE	190	194	80.6	6	HHHHH	1O6U:C	ENVQD	21	25	81.16	6	HHTTT
2PV2:D	QVNEA	191	195	58.46	7	HHHHH	3V5U:A	AENVQ	256	260	58.58	5	CCHHH
2PVB:A	EEDEL	59	63	44.04	8	CHHHH	1MFT:B	LEDEE	38	42	43.76	9	HHHHH
2PVB:A	AFLADG	84	89	31.78	8	HHHHHH	3SK2:A	GDALFA	50	55	31.8	8	TTCEEE
2Q0S:A	TQVLT	116	120	52.82	6	HHHHT	3QBM:A	TLVQT	136	140	52.02	8	HHHHH
2Q22:C	GICAD	55	59	19.12	9	EEEES	1OF8:B	DACIG	342	346	19.8	8	SCBBC
2Q3E:A	TAIKD	167	171	43.02	7	SHHHH	2V7F:A	DKIAT	130	134	43.46	9	HHHHH
2Q3G:A	GKAAQ	37	41	52.32	9	SHHHH	1SYQ:B	QAAKG	610	614	52.18	7	HHHHH
2Q52:A	HAETL	75	79	8.425	13	CCSBH	3QY9:D	LTEAH	131	135	8.88	11	EEEEE
2QDQ:A	AQIRQ	2523	2527	91.56	6	HHHHH	2BTI:A	QRIQA	49	53	92.36	6	HHHHH
2QE8:B	FGSDG	297	301	35.32	7	ECTTS	1I07:A	GDSGF	48	52	35.7	7	SCEEE
2QE8:B	VLDAE	200	204	26.06	7	EECTT	1MKY:A	EADLV	81	85	26.68	9	TCSEE
2QE9:B	KLSPV	140	144	62.16	5	TCCCC	2IG6:A	VPSLK	93	97	62.52	6	SGGGG
2QF4:B	TLDAG	131	135	16.9	10	EESCC	2F3L:A	GADLT	61	65	16.36	11	TCBCT
2QFA:B	NIEIL	55	59	54.62	8	HHHHH	1YDX:A	LIEIN	166	170	54.72	7	HHHHH
2QHL:A	TIKFT	100	104	32.92	8	EEEEE	2C5S:A	TFKIT	105	109	32.4	7	EEEEE
2QMQ:A	LFSQE	201	205	60.16	8	HSCHH	2XGT:A	EQSFL	291	295	59.2	7	EEEEE
2QOL:A	EISVA	647	651	37.54	7	BCCEE	3O5Y:A	AVSIE	142	146	37.14	7	HHHHH
2QOL:A	AARNI	748	752	31.2	8	CGGGE	4A56:A	INRAA	64	68	32.06	7	HHHHH
2QPX:A	LYHVA	210	214	23.56	9	HHHHH	1QQF:A	AVHYL	1024	1028	23.88	10	HHHHH
2QPX:A	VPDDP	104	108	64.36	4	CCSSB	3L4A:A	PDDPV	54	58	64.86	5	CSSHH
2QPX:A	SEFVD	5	9	65.86	5	HHHHH	3KBY:A	DVFES	66	70	65.52	6	CTTSC
2QPX:A	LAKEF	61	65	63.1	7	HHHHH	2RCI:A	FEKAL	77	81	63.22	5	HHHHH
2QQB:A	TQLLD	180	184	68.16	6	HHHHH	3SWF:C	DLLQT	17	21	67.34	8	HHHHH
2QSW:A	ILAAI	325	329	28.4	8	HHHHH	3APS:A	IAALI	771	775	28.64	8	HHHHH
2QSW:A	TEVIG	339	343	33.34	7	EEESS	3PQK:F	GIVET	76	80	33.06	7	TSEEE
2QSW:A	AKLPII	280	285	72.57	6	CSSCHH	1MML:A	IIPLKA	49	54	73.42	8	CCCBCT
2QSX:B	YVVIP	187	191	12.5	10	EEECC	1SLU:A	PIVVY	114	118	13.42	8	CEEEE
2QTQ:D	DVVDI	32	36	40.02	5	TCSCC	3K5I:B	IDVVD	375	379	39.74	7	HHHHH
2R01:A	IAAQT	115	119	37.34	6	HHHHH	3DHX:A	TQAAI	78	82	38.16	8	HHHHH
2R5O:B	LKSSF	423	427	33.44	6	CCCEE	1PZX:A	FSSKL	87	91	32.9	8	CCTTT

2R6Q:A	TATASV	142	147	48.65	7	BCSSCC	3M3G:A	VSATAT	124	129	47.78	6	EECEEE
2R6V:A	AHVSW	154	158	49.36	8	EEEET	2VGO:A	WSVHA	237	241	48.84	8	TCEEC
2R8O:A	DAASI	220	224	37.86	6	CHHHH	3CTZ:A	ISAAD	360	364	38.58	7	HHHHH
2R8O:A	EEQLA	530	534	79.86	5	HHHHH	3BDI:A	ALQEE	2	6	80.3	4	CCEEE
2R8O:A	QERTE	526	530	82.44	8	CCCCH	2JC9:A	ETREQ	132	136	83.2	7	HHHHH
2R8U:A	QAGFK	79	83	59.14	8	HHHHH	3K6G:C	KFGAQ	384	388	58.76	10	HHCHH
2RAF:C	LEVKD	150	154	58.96	7	CEEEE	3JZ0:A	DKVEL	223	227	58.42	6	CHHHH
2RB5:A	AIINN	48	52	42.82	5	GGCCS	3F6C:B	NNIIA	107	111	43.18	6	HHHHH
2RB5:A	FADVT	178	182	2	12	CCEEE	1AQ0:B	TVDAF	210	214	2.04	12	HHHHH
2RB5:A	VEEHN	107	111	68.16	7	ECSSC	1B8O:A	NHEEV	256	260	67.72	7	CHHHH
2RB5:A	FGDGG	209	213	13.98	8	EECSG	3IVE:A	GGDGF	470	474	13.24	7	TGGGC
2RC3:A	DLVKD	142	146	39.4	7	HHHHH	1G60:B	DKVLD	58	62	38.52	8	HHHHH
2RFR:A	GSAAA	143	147	40.7	5	SCHHH	1QVE:A	AAASG	87	91	40.24	5	EETTE
2RGQ:C	YDEAT	81	85	57.28	7	SSEEE	3C1D:B	TAEDY	52	56	58.18	5	CHHHH
2RIK:A	TSLKV	181	185	31.98	10	EEEEE	2E8E:A	VKLST	102	106	32.8	11	HHHHH
2RIK:A	NAAGS	172	176	43.34	5	CSSCE	3AKH:A	SGAAN	109	113	42.6	6	ECCSC
2RIK:A	ITLKA	276	280	72.46	4	EEECC	2QSQ:B	AKLTI	0	4	73.02	3	CCCEE
2RIK:A	TSLKV	181	185	31.98	10	EEEEE	1N2F:A	VKLST	31	35	31.34	8	EEECC
2RK3:A	VKAPL	181	185	62.86	4	HHGGG	1H6H:A	LPAKV	89	93	61.92	3	CCCCC
2RK3:A	FALAI	164	168	8.78	8	HHHHH	3D9S:D	IALAF	45	49	9	10	HHHHH
2RK3:A	VKEIL	88	92	43.8	8	HHHHH	2F6U:B	LIEKV	2050	2054	43.54	6	HHHHH
2SAK:A	ITKVV	128	132	52.82	8	EEEEE	1EF1:B	VVKTI	33	37	53.46	6	HHHHH
2UV4:A	LGIFV	217	221	45.58	6	HHHHH	2GFF:A	VFIGL	90	94	45.3	4	EEEEE
2UXY:A	SLQGI	61	65	4.46	8	TTTBC	2QE9:B	IGQLS	129	133	3.68	7	HHHHH
2UY2:A	FDSAV	144	148	40.56	7	TTTCC	3NRV:D	VASDF	107	111	40.94	9	HHHHH
2V1Q:A	QDELT	16	20	73.22	5	TTBCC	2B8M:A	TLEDQ	60	64	74.12	4	EETTS
2V33:B	ATAKV	342	346	16.46	11	SEEEE	2CLB:A	VKATA	31	35	16.6	9	HHHHH
2V3G:A	AYQAL	207	211	32.82	7	HHHHH	3QFT:A	LAQYA	392	396	32.08	8	HGGGB
2V3G:A	PIIIA	217	221	10.42	10	CEEEE	2PNL:J	AIIIP	561	565	10.44	8	EEEEE
2V3I:A	YRLGN	266	270	46.76	8	TTTTC	2FPW:B	NGLRY	145	149	45.8	9	EEEEC
2V3I:A	VTQFE	291	295	19.42	12	EEEEC	2C2U:A	EFQTV	57	61	20.36	11	HHHHH
2V3I:A	GSCST	395	399	42.68	6	SSSCT	2OLM:A	TSCSG	50	54	41.76	5	HHHHH
2V5T:A	AEVVC	132	136	22.52	8	EEECC	2E56:A	CVVEA	133	137	23.04	8	EEEEE

2V6K:A	RRAAP	201	205	60.7	8	HHHSG	3D3B:A	PAARR	3	7	61.18	9	CHHHH
2V76:B	SQILE	44	48	41.24	6	TCSEE	2ZXK:A	ELIQS	154	158	42.1	8	EEEES
2V7F:A	GFVEK	109	113	49.1	10	TSEEE	3U62:A	KEVFG	245	249	48.44	8	HHHHG
2VBK:A	DLIEQ	116	120	63.34	10	HHHHH	3IRS:A	QEILD	110	114	62.86	8	HHHHH
2VDJ:A	KTNVT	132	136	49	8	HHHEE	3IG2:B	TVNTK	673	677	48.16	10	EEECT
2VFO:A	GFDLG	393	397	7.14	9	SEEEC	2WBN:A	GLDFG	264	268	6.36	9	EEECC
2VFO:A	VATLK	221	225	48.12	7	HHTCE	1G8M:A	KLTAV	531	535	47.56	6	TCCCE
2VHA:B	RAKAK	190	194	55.64	8	HTTSS	3U9Q:A	KAKAR	230	234	55.56	9	HHHHH
2VHA:B	PQSQE	205	209	56.22	7	CSCEE	3CNH:A	EQSQP	82	86	56.32	6	HTCCB
2VIF:A	GKLAN	396	400	60.68	6	HHTTT	1YG9:A	NALKG	129	133	59.7	5	CTTSC
2VIF:A	VDLIE	455	459	51.58	7	HHHHH	2VWS:A	EILDV	163	167	50.62	5	HHHTS
2VKJ:A	KALNL	101	105	50.52	7	HHHHH	2Y1K:A	LNLAK	244	248	49.78	9	HHHHH
2VLQ:B	IRVTT	119	123	11.88	11	EEEEC	2B7U:A	TTVRI	69	73	11.24	11	EEEEE
2VLQ:B	RIADK	37	41	57.3	7	HHHHH	1EUV:A	KDAIR	602	606	58.04	7	HHHHH
2VN5:A	LDNTIT	89	94	33.67	6	ECTTSS	3IJW:B	TITNDL	16	21	33.78	8	HHHHHH
2VN5:B	DNEVN	42	46	70.1	7	SSCCS	2WAS:B	NVEND	1780	1784	69.26	6	CSSCH
2VPT:A	LIDQI	140	144	21.52	11	HHHHH	2Q9K:A	IQDIL	144	148	21.5	9	HHHHH
2VSM:A	VKPKS	321	325	82.98	5	SSCCT	1LSH:B	SKPKV	1356	1360	83.98	3	CCCCE
2VT3:A	SFFRK	74	78	76.46	8	HHHHH	1QR0:A	KRFFS	120	124	77.32	9	SSSSC
2VU6:A	KGDAA	19	23	53.4	5	CSSSE	3AMR:A	AADGK	239	243	53.7	5	CGGGC
2VU6:A	PTTVT	188	192	56.64	5	CCCEE	1MN8:D	TVTTP	4	8	56.28	5	CCCCH
2VU9:A	IAKLV	1258	1262	20	10	EEEEE	3MZ0:A	VLKAI	83	87	19	11	HHHHH
2VU9:A	GYMYL	1132	1136	16.96	9	CEEEE	1Z4V:A	LYMYG	393	397	16.74	9	EEEET
2VU9:A	RDTHR	1061	1065	82.76	7	CCTTC	1W1O:A	RHTDR	494	498	82.58	6	CCSSH
2VWS:A	TLLIP	93	97	2.08	7	EEEEC	1W9M:A	PILLT	305	309	1.14	9	CEEES
2VWS:A	GIDGV	169	173	9.68	7	TCCEE	3KV1:A	VGDIG	183	187	9.44	9	EEEET
2VWS:A	DLYHQL	56	61	25.6	9	HHHHHH	3CX5:Q	LQHYLD	130	135	26.27	11	HHHHHH
2VXN:A	IAAAN	7	11	2.12	10	EEEEE	2IU5:A	NAAAI	142	146	2.76	10	HHHHH
2VYN:D	GPMAGIL	265	271	40.2	6	TTTTTTE	2EFJ:A	LIGAMPG	132	138	39.59	8	EEEECCS
2VZC:A	PDKLN	258	262	85.92	5	HHHHH	3R0R:A	NLKDP	220	224	86.4	5	CCCCC
2VZC:A	DSFEQ	318	322	56.66	7	CSHHH	3G3Z:A	QEFSD	105	109	55.82	9	HHHHH
2VZC:A	LLEGY	302	306	63.56	6	HHTTC	1HNJ:A	YGELL	185	189	64.1	4	GGGGE
2VZP:B	VTVPS	45	49	52.78	6	EEESS	3BUT:A	SPVTV	55	59	52	5	CCEEE

2VZP:B	AVATT	111	115	30.88	9	EEECS	1F00:I	TTAVA	668	672	30.9	7	SEEES
2W15:A	PRYIE	5	9	49.46	7	CEECC	2HJ1:B	EIYRP	81	85	50.38	9	EEECS
2W1V:B	RADLY	303	307	42.8	7	CTTTE	2X3H:C	YLDAR	22	26	41.88	8	HHHHT
2W39:A	GHATN	148	152	57.36	8	SEEEE	1LC5:A	NTAHG	5	9	57.88	6	CCSSS
2W4J:A	ESLTE	109	113	64.52	7	CCCCH	3BI1:A	ETLSE	744	748	65.3	7	HTTSC
2W4J:A	KKRMT	261	265	86.16	10	GGSCC	2A1L:A	TMRKK	256	260	85.98	9	HHTTS
2W6P:B	EKKLG	441	445	93.14	4	HHHHC	3BJ5:A	GLKKE	289	293	93.5	5	TCCGG
2W72:A	ALTNA	65	69	31.26	9	HHHHH	3GTZ:C	ANTLA	40	44	30.92	8	HHHHH
2W7Z:A	SNVRF	70	74	32.9	11	EEEEE	2ZW2:A	FRVNS	49	53	32.2	11	EEEEC
2W8T:A	GAIVFS	127	132	6.13	11	EEEEES	1YPF:A	SFVIAG	141	146	5.2	12	SEEEEE
2W8T:A	GKEAL	88	92	67.1	8	HHHHH	3SAO:A	LAEKG	25	29	67.6	8	HHHGG
2W8T:A	GSRML	101	105	55.34	8	SCTTT	2QDX:A	LMRSG	194	198	54.86	7	HHHHS
2W8X:B	RSLCE	23	27	55.3	6	HHHHH	2BLA:A	ECLSR	181	185	54.3	7	HHHHT
2W91:A	DLVKP	341	345	75.9	3	TTTGG	2QKP:A	PKVLD	383	387	76	4	HHHHH
2W91:A	VIDAG	273	277	2.14	10	HHHHH	3GMI:A	GADIV	111	115	1.92	10	TCSEE
2W91:A	GNKDA	734	738	62.64	5	CCTTE	3P94:A	ADKNG	174	178	62.24	5	HHHTT
2W91:A	AVGKN	784	788	28.62	11	EEETT	3QFT:A	NKGVA	489	493	28.06	10	EECHH
2W91:A	KQSLK	717	721	82.28	7	EEEEE	4A1U:A	KLSQK	16	20	82.5	6	HHHTT
2W91:A	DGYFI	330	334	4.64	10	CEEEE	1D2S:A	IFYGD	55	59	4.52	8	EEEEE
2WAG:A	YHFFS	73	77	13.86	10	EEECC	2BKR:A	SFFHY	114	118	14.76	11	EEEEE
2WAG:A	VDKYF	52	56	54.54	6	ECTTH	2OB0:C	FYKDV	35	39	54.9	6	HHHHH
2WAO:A	TGYGED	303	308	26.97	11	TCCCGG	3G14:A	DEGYGT	116	121	26.05	10	HTTCBB
2WAS:B	ALKDI	1833	1837	33.96	6	CSTTE	4AFL:D	IDKLA	42	46	34.08	7	HHHHH
2WBN:A	IDELV	291	295	21.68	8	EEEEE	2I53:A	VLEDI	246	250	21.18	10	HHHHH
2WBN:A	INRIE	337	341	60.02	9	CTTEE	1EF1:B	EIRNI	244	248	60.86	7	GEEEE
2WF7:A	SQAGI	171	175	19.8	7	SHHHH	3PPQ:A	IGAQS	36	40	19.44	8	EEEES
2WF7:A	APSES	161	165	43.4	6	CGGGE	3F0H:A	SESPA	284	288	43.22	7	CSSBB
2WF7:A	KNDNY	76	80	32.34	10	HHHHH	3SEB:A	YNDNK	217	221	32.58	10	GGGCC
2WFI:A	PKTCE	34	38	48.08	7	HHHHH	1YKW:A	ECTKP	341	345	47.08	7	HHHSC
2WHL:A	LALVI	216	220	2.96	8	CCEEE	3FOT:A	IVLAL	314	318	2.08	8	HHHHH
2WKJ:C	VFRGL	251	255	24.14	8	HHHHH	2QV3:A	LGRFV	458	462	24.4	8	CCTTE
2WL1:A	YRVGS	724	728	63.08	5	TTTTE	1YQH:A	SGVRY	34	38	63.56	7	SCSEE
2WM3:A	LENIKK	114	119	47.27	9	CCCHHH	2A6S:D	KKINEL	25	30	46.28	10	HHHHHH

2WM3:A	LANMF	257	261	13.42	9	HHHHH	3F6G:A	FMNAL	433	437	13.96	9	HHHHH
2WM3:A	LGLHY	104	108	45.28	8	HTCSE	1U02:A	YHLGL	117	121	46.16	6	EECTT
2WNF:A	GSKTT	185	189	39.84	8	CSCCS	1UAD:D	TTKSG	77	81	40.78	8	EETTT
2WNV:D	QGSEA	206	210	70.58	4	CSSSC	1VGW:E	AESGQ	147	151	70.42	5	ESSSB
2WNV:D	GLFQV	174	178	23.32	6	SSCEE	2WAO:A	VQFLG	102	106	22.54	8	EEEEE
2WOJ:C	STDPA	55	59	15.34	4	ECCSS	3FCX:A	APDTS	80	84	15.28	6	EECSC
2WOY:A	AGTDL	1338	1342	44.76	8	TTCBC	4FIV:A	LDTGA	29	33	45.44	7	ECTTC
2WOY:A	SENLD	1269	1273	55.48	6	SCCCT	1SQ9:A	DLNES	374	378	56	8	TTSCE
2WQ4:B	SLSAI	122	126	1.74	8	EEEEE	2IUW:A	IASLS	204	208	1.92	10	EEEEE
2WQR:A	LTLSQ	297	301	36.28	8	EEEEH	2NXY:B	QSLTL	1112	1116	35.5	9	CCEEE
2WQR:A	FSRLE	506	510	13.34	11	EEEEE	1VCL:A	ELRSF	12	16	12.66	11	CEEET
2WUH:A	ESTAA	54	58	40.84	7	GGGSG	1MML:A	AATSE	229	233	40.22	8	EESSH
2WUJ:B	NKTFT	10	14	77.36	6	HCCCC	3ZZY:A	TFTKN	215	219	76.76	5	EEEET
2WY3:A	AEGHL	24	28	25.82	8	EEEEE	3OKF:B	LHGEA	267	271	24.96	7	CHHHH
2WY3:A	KRRAK	40	44	79.66	6	TTEEE	1YKW:A	KARRK	220	224	79.06	8	HHHHH
2WZ1:A	TGVIG	530	534	17.04	6	EEEEC	1LO7:A	GIVGT	55	59	17.32	8	CEEEC
2WZ8:A	TDIID	113	117	54.06	7	EEEEE	3E3V:A	DIIDT	196	200	53.16	6	HHHHH
2X3G:A	GDTEK	28	32	83.32	6	SCHHH	3LVK:B	KETDG	66	70	84.2	4	ECCSS
2X3H:C	GQSLY	16	20	53.28	8	TEEHH	2ZKM:X	YLSQG	16	20	52.28	7	HHHHC
2X3H:C	NDLLS	359	363	51.42	5	SCTTS	3D7R:B	SLLDN	164	168	51	7	HHHHT
2X46:A	DTSTV	97	101	39.32	9	EEEEE	1SO7:A	VTSTD	127	131	39.86	8	EEESS
2X49:A	TVYFD	424	428	43.92	5	ECCTT	3DY0:A	DFYVT	203	207	43.52	7	EEESS
2X5Y:A	VMFVA	839	843	0.52	8	EEEEE	3FTD:A	AVFMV	124	128	0.7	9	EEEEE
2X5Y:A	ELLDK	766	770	73.42	6	HHHHH	2B5G:A	KDLLE	39	43	73.54	6	HHHHH
2X6W:A	VSGHL	435	439	26.76	9	CBCEE	2BS2:F	LHGSV	181	185	27.5	11	HHHHH
2X6W:A	VYNAP	524	528	31.28	8	EECCT	1OOT:A	PANYV	51	55	31.68	6	EGGGE
2XBL:C	LTYIT	6	10	48.66	7	HHHHH	3NRE:A	TIYTL	2	6	48.5	6	CEEEE
2XEU:A	RLIVS	21	25	27.96	9	CCEEE	4ADN:B	SVILR	24	28	27.8	10	HHHHH
2XFA:B	EEELK	133	137	68.76	8	HHHHH	1Q8B:A	KLEEE	37	41	68.88	9	HHHHH
2XFD:A	QVGGT	20	24	44.64	4	EETTE	3LLP:A	TGGVQ	320	324	44.34	6	TSBEE
2XFR:A	EMILQ	465	469	80.42	5	HHHHG	3RYD:A	QLIME	26	30	79.88	5	HHHHT
2XFR:A	YDKYL	207	211	41.64	9	CSHHH	1HXA:A	LYKDY	345	349	42.5	11	HHHHH
2XGT:A	FGEQS	289	293	73.78	4	TTEEE	2ZAY:A	SQEGF	29	33	74.26	5	HHHTE

2XGT:A	NTECT	173	177	15.16	11	CTTCE	1D3B:K	TCETN	19	23	15.3	13	EEEET
2XHG:A	EVTFN	364	368	20.5	11	SEEEE	1O6U:C	NFTVE	378	382	19.78	10	EEEEE
2XOM:A	TGEFS	32	36	73.4	5	TSSCT	1K7C:A	SFEGT	227	231	72.86	3	CCSSC
2XT1:A	GPKEP	156	160	74.02	8	CTTCC	1QSA:A	PEKPG	96	100	74.8	6	CSCCS
2XTM:B	SKLVA	169	173	53.44	8	HHHHH	3JWI:A	AVLKS	23	27	54.04	8	HHHHH
2XTS:C	LVISG	324	328	3.54	10	EEEEE	3SX2:H	GSIVL	149	153	3.8	9	EEEEE
2XTS:D	ITAFL	137	141	2.46	8	HHHHH	1W9M:A	LFATI	66	70	1.66	9	HHHTS
2XTS:D	VTPED	207	211	50.96	9	CSCBC	3RPC:D	DEPTV	156	160	50.34	8	TSCCE
2XVC:A	FLDIE	225	229	39.78	10	EEEHH	1EJF:B	EIDLF	52	56	40.64	9	EEEBS
2XWV:A	INSIAD	135	140	49.9	5	CCSGGG	2WKB:A	DAISNI	25	30	49.42	7	HHHHHH
2XZZ:A	TLRQS	751	755	72.86	6	EEEEE	3BUU:A	SQRLT	122	126	72.06	8	TSEEE
2Y0O:A	LQLFV	42	46	18.4	8	EEEEE	3CT9:B	VFLQL	115	119	17.5	7	HHHHH
2Y27:A	GELVF	284	288	4.38	10	EEEEE	2JER:H	FVLEG	154	158	5.26	11	EECCG
2Y27:A	TAPDT	380	384	69.1	6	TTTCH	3C3M:A	TDPAT	70	74	69.6	7	HSTTT
2Y27:A	DDLKT	52	56	68.34	5	GGCSS	2BK8:A	TKLDD	65	69	68.38	7	CGGGC
2Y27:A	FTGGL	141	145	12.82	7	SHHHH	3DO8:B	LGGTF	5	9	13.62	5	EEECC
2Y43:A	NNRIL	74	78	39.04	8	ECHHH	3K0L:B	LIRNN	136	140	39.78	9	HHHHH
2Y4Z:A	RVLLE	70	74	44.12	8	HHHHH	3T6O:A	ELLVR	64	68	43.42	8	HHHHH
2Y6U:A	PNLFH	159	163	52.7	5	TTSCS	3L2H:A	HFLNP	23	27	53.44	5	ETTEE
2Y7L:A	CFTAG	133	137	39.98	7	SCCSE	3AG3:K	GATFC	21	25	40.4	8	HHHHH
2Y7L:A	DLADS	127	131	15.98	11	HHHHT	3K4I:A	SDALD	29	33	15.56	10	HHHHH
2Y7L:A	GTVKL	109	113	29.18	8	EEEEE	3AWG:C	LKVTG	439	443	29.42	7	BEEEE
2Y8D:A	KDPKL	2387	2391	97.6	7	HCHHH	3LRU:A	LKPDK	1954	1958	98.26	5	TCCST
2Y9U:A	RQLHE	605	609	96.22	8	HHHHH	2F42:A	EHLQR	240	244	95.46	8	HHHHH
2YC3:A	SVILL	80	84	3.84	11	EEEEE	4AJJ:A	LLIVS	132	136	3.52	10	EEECS
2YFO:A	VITRS	158	162	0.14	12	EEEEE	3VBL:E	SRTIV	85	89	1.12	13	TTCEE
2YFO:A	KDQFN	263	267	56.62	7	ECTTS	3GNZ:P	NFQDK	202	206	57.1	8	THHHH
2YHG:A	NGESI	796	800	45.44	6	SSCCS	2OR7:B	ISEGN	69	73	44.74	6	GGGTB
2YHG:A	PGLND	750	754	10.28	9	TTTCC	2QPX:A	DNLGP	289	293	9.96	11	HHHSG
2YVE:B	DVLSK	155	159	81.76	6	SCCCH	3FFY:A	KSLVD	146	150	82.32	5	HHTTT
2YXT:A	DTVVI	181	185	14.54	10	SEEEE	1MDO:A	IVVTD	197	201	14.2	9	EEEES
2Z4U:A	VLVKL	50	54	17.94	9	EEEEE	3NBM:A	LKVLV	456	460	17.78	10	EEEEE
2Z4U:A	NPNPK	195	199	34.96	9	CCCHH	2BFF:A	KPNPN	357	361	35.46	11	BCCGG

2Z72:A	GELLL	313	317	5.64	9	CCEEE	2YFO:A	LLLEG	513	517	6.34	10	CEEEE
2Z72:A	YDIAT	166	170	34.74	10	HHHHH	2YVE:B	TAIDY	12	16	35.28	9	HHHHH
2Z72:A	DIFDR	114	118	33.64	10	CCSSS	1MG7:B	RDFID	105	109	34.28	8	HHHHH
2Z80:A	SNLSS	113	117	54.52	5	SSCCH	1FYH:E	SSLNS	190	194	54.4	6	CTTCC
2ZAY:A	AASIS	22	26	45.3	6	HHHHH	3T5S:A	SISAA	75	79	45.48	6	HHHHH
2ZCM:A	LHDEL	120	124	42.36	10	HHHHH	3MJG:A	LEDHL	91	95	41.58	8	EEEEE
2ZHJ:A	LHVFDE	206	211	43.42	8	HHHHHH	2OCT:A	EDFVHL	62	67	42.98	9	TCEEEE
2ZHJ:A	LTAGG	155	159	43.6	6	HHHCC	2FTB:A	GGATL	114	118	42.72	5	TTEEE
2ZK9:X	VTEKR	96	100	68.92	7	CEEEE	3B0B:C	RKETV	9	13	68.06	6	CHHHH
2ZK9:X	ATLNS	9	13	45.04	6	HHHHH	1M1E:B	SNLTA	31	35	45.84	8	CCCCH
2ZKM:X	QMVAL	622	626	0.44	14	SEECB	2FZV:A	LAVMQ	140	144	0.16	13	EEEEE
2ZNR:A	LPEAI	371	375	18.58	9	CTTCE	3U02:C	IAEPL	32	36	18.78	7	TSEES
2ZOU:B	IDEEE	117	121	91.34	5	SCTTT	3HHT:B	EEEDI	21	25	90.42	5	GGGCC
2ZPT:X	VVSYY	136	140	8.14	8	HHHHH	3ON9:B	YYSVV	212	216	9.02	10	EEEEE
2ZUX:B	SLIMT	318	322	1.66	9	EEEEE	2DVT:C	TMILS	54	58	1.7	10	EEEEE
2ZUX:B	RGLVA	47	51	15.26	7	CCCEE	3V7Q:B	AVLGR	170	174	14.38	7	HHHHH
2ZUX:B	TGKVI	243	247	59.14	6	TSCBC	1FR3:H	IVKGT	16	20	59.66	5	EEECS
2ZUX:B	FRDAA	432	436	10.3	13	EEETT	2QTQ:D	AADRF	169	173	10.88	11	HHHHH
2ZW2:A	KIIET	37	41	49.24	6	TEEEE	2Y1K:A	TEIIK	258	262	49	5	HHHHH
2ZXY:A	MKPQL	61	65	70.88	6	HGGGG	1W98:B	LQPKM	125	129	71.06	6	CCHHH
3A57:A	NVEVS	32	36	54.94	7	CEEEE	3AJM:A	SVEVN	71	75	54	6	HHHHH
3A72:A	PNNGL	165	169	63.8	5	CCTTC	2PTH:A	LGNNP	123	127	64.1	7	TTSCC
3A72:A	GTETY	252	256	94.82	4	TSSSC	3U7I:D	YTETG	126	130	95.8	2	EETTE
3A9J:B	FVKTL	4	8	44.96	8	EEECT	1VKK:A	LTKVF	117	121	45.06	6	CCCEE
3A9S:C	VGVSI	85	89	7.54	10	EEEEE	1YPF:A	ISVGV	90	94	8.2	9	EEECC
3AA0:A	IKIIE	229	233	41.78	9	HHHHH	2UX0:F	EIIKI	398	402	41.16	8	HHHHH
3ACX:A	QSIEK	68	72	67.96	7	HHHHH	3IJD:B	KEISQ	31	35	67.86	8	HHHHH
3ACX:A	LSDHE	145	149	69.02	6	HCSSC	2UVP:D	EHDSL	150	154	68.48	5	TTCTT
3AG3:B	QEVET	59	63	75.86	6	TTHHH	3T6O:A	TEVEQ	27	31	76.82	4	HHHHH
3AG3:L	FFGSG	28	32	29.78	8	HHHHH	2WQR:A	GSGFF	500	504	29.48	7	SSSEE
3AGN:A	NGDRP	32	36	68.62	8	TTCCG	2E6F:A	PRDGN	49	53	68.78	8	CBCCS
3AGN:A	AIQGA	22	26	21.66	8	HHHHH	1JB0:C	AGQIA	35	39	21.44	6	TSEEE
3AJ6:B	DSLIQ	225	229	54.96	7	SSGGG	3KH0:B	QILSD	850	854	55.14	7	EECSS

3AJD:A	IKGTL	253	257	34.38	7	STTCE	2D1S:A	LTGKI	528	532	34.16	5	TTSCB
3AK9:J	SKTPL	100	104	65.68	6	HHCCS	2F0C:A	LPTKS	62	66	66.44	5	CCEEE
3AK9:J	KTPLK	101	105	80.94	6	HCCSC	3T7K:A	KLPTK	944	948	81.94	8	TSSSC
3AKH:A	EKGQIA	121	126	54.2	6	EEEECC	2QE9:B	AIQGKE	55	60	54.47	6	HHHTCC
3AKH:A	KVFLT	206	210	19.1	8	EEEEE	4DOV:A	TLFVK	152	156	19.84	10	EEEEE
3AMR:A	DPKTP	34	38	71.3	4	CSSCG	3I38:J	PTKPD	275	279	70.38	5	CSCCC
3AMR:A	DPKTP	34	38	71.3	4	CSSCG	3HPC:X	PTKPD	94	98	71.02	3	CCCCC
3AMR:A	GENID	309	313	44.78	11	SSCEE	1N7O:A	DINEG	765	769	45.34	9	GTBTT
3AON:A	AEEIE	157	161	31.22	8	HHHHH	3ELF:A	EIEEA	261	265	30.82	7	HHHHH
3AQI:B	LPMSV	765	769	31.92	6	CBHHH	3AJM:A	VSMPL	18	22	31.18	8	GGHHH
3ARC:H	LILDG	53	57	53.16	5	SCCTT	2HC1:A	GDLIL	1826	1830	53.48	7	TTEEE
3ARC:u	ILREN	74	78	57.5	7	HHHHH	2WM9:A	NERLI	393	397	56.82	7	HHHHC
3AWU:A	EFVRT	33	37	50.56	7	HHHHH	3H9M:A	TRVFE	64	68	50.76	7	TSHHH
3AWU:A	SLGGS	142	146	51.54	5	CTTSS	3DQG:A	SGGLS	529	533	51.9	5	SSSSC
3AWU:B	ELQGA	114	118	39.74	7	HHTTC	1B0B:A	AGQLE	103	107	39.22	7	HHHHH
3B21:A	TLQKM	48	52	51.08	9	HHHHH	1Q0Q:A	MKQLT	1	5	50.76	10	CEEEE
3B5N:I	NRVRK	79	83	77.9	8	HHHHH	2EX4:B	KRVRN	110	114	77.72	9	GGEEE
3B5N:K	NKVAD	486	490	46.22	9	HHHHH	1V7P:C	DAVKN	160	164	45.94	7	HHHHH
3B5O:A	QELQE	75	79	67.96	8	HHHHH	3D3M:A	EQLEQ	800	804	68.7	8	HHHHH
3B64:A	ACVRVE	58	63	27.5	11	EEEEEE	3D06:A	EVRVCA	271	276	27.23	12	EEEECS
3B79:A	PAILVL	68	73	5.25	9	SEEEEE	3SWN:F	LVLIAP	93	98	6.2	9	EEEEEE
3B7E:A	LTGNS	85	89	35.98	7	CCCCC	1U83:A	SNGTL	108	112	36.92	5	CCSSS
3B9W:A	VSIAL	156	160	29.2	7	HHHHH	3K7I:B	LAISV	114	118	28.96	7	HHHHH
3B9W:A	PIESDA	169	174	65.58	7	CCCCCH	3O2T:A	ADSEIP	199	204	65.65	7	TTCCCC
3B9W:A	ALAGG	243	247	1.24	5	TTHHH	3V5U:A	GGALA	242	246	1.46	7	HTHHH
3B9W:A	ATAIV	195	199	18.88	6	HHTTS	2QSA:A	VIATA	84	88	18	8	HHHHH
3B9W:A	LLALF	109	113	36.78	6	HHHHH	3TFW:B	FLALL	52	56	37.68	8	HHHHH
3BB0:A	LFALV	334	338	0.82	7	HHHHH	2PQ5:A	VLAFL	149	153	1.4	8	HHHHH
3BB0:A	KEEDL	317	321	88.94	6	SSSCS	1R0U:A	LDEEK	107	111	88.48	5	ECSSE
3BCW:A	EGYTG	100	104	27.66	8	TTCCC	3QY3:A	GTYGE	103	107	28.64	9	SCCEE
3BF7:A	VLRAI	244	248	35.32	8	HHHHH	1Z4R:A	IARLV	539	543	34.7	6	HHHHH
3BFQ:G	DSGNT	91	95	71.96	5	TTSCB	3I26:B	TNGSD	321	325	72.74	6	EEEEE
3BHD:A	PELSL	43	47	48.24	6	TTCHH	3IKK:A	LSLEP	8	12	49.2	5	SEEES

3BHD:A	GGTLE	26	30	59.3	7	TCEEE	2WBM:A	ELTGG	220	224	59.26	6	HHTTT
3BHD:A	EERLQ	19	23	80.36	7	HHHHH	3CNH:A	QLREE	188	192	81.3	9	HHHHH
3BHW:A	VENVL	42	46	57.42	6	TSCTT	3C8C:A	LVNEV	210	214	57.42	6	HHTCS
3BHW:A	GLLLE	25	29	46.76	5	HHHHH	1FS1:A	ELLLG	115	119	45.8	7	HHHHH
3BJQ:A	IADSI	168	172	27.56	5	HHHHH	2GJV:F	ISDAI	77	81	27.58	5	HHHHH
3BJQ:A	PGIDL	102	106	45.06	6	CCHHH	1V7P:C	LDIGP	173	177	45.48	8	SCBST
3BL4:A	SSPVD	86	90	54.9	6	SSGGG	3QO4:A	DVPSS	178	182	55.82	6	CSCBS
3BMX:A	ALIAV	576	580	0.92	8	EEEEC	3P1V:B	VAILA	178	182	0.96	9	EEEEE
3BMX:A	VQFPA	271	275	18.42	10	CBCTT	1DZK:B	APFQV	33	37	18.44	8	STTCC
3BNJ:A	QEWKK	475	479	71.16	9	HHHHH	3BPT:A	KKWEQ	73	77	70.34	7	HHHHH
3BNJ:A	IAKGR	482	486	74.8	9	HHHTS	1NH9:A	RGKAI	34	38	74.26	7	EGGGH
3BON:A	VTLAH	219	223	7.16	9	HHHHH	1RYL:B	HALTV	160	164	6.5	10	EEEEE
3BON:A	VKAFK	33	37	15.84	9	EEEEE	1R26:A	KFAKV	51	55	15.32	10	EEEEE
3BON:A	LTSIV	108	112	17.42	9	HHHHH	2FN9:A	VISTL	7	11	16.76	9	EESCS
3BPT:A	DKINS	266	270	66.08	5	HHHHH	3TD3:E	SNIKD	239	243	65.66	7	CCCCG
3BPT:A	KPADL	360	364	58.64	6	SSCSG	1XVX:A	LDAPK	287	291	58.26	4	SCCCC
3BPU:A	GLKEG	679	683	53.9	6	TCCTT	3IPF:B	GEKLG	55	59	53.5	6	EEEEE
3BQP:B	FVAEY	50	54	40.06	7	HHHHH	2CWZ:D	YEAVF	8	12	40.76	8	CEEEE
3BT5:A	LARQI	157	161	43.86	8	HHHHH	2VQC:A	IQRAL	43	47	44.4	7	HHHHH
3BT5:A	ASEAE	107	111	71.18	6	CCHHH	2NVN:A	EAESA	70	74	71.92	5	EEECS
3BUU:A	RDLWV	104	108	46.36	11	TEEEE	1S9U:A	VWLDR	90	94	46.16	9	HHHST
3BUX:B	ADAAE	184	188	55.18	5	HHHHH	2HIP:B	EAADA	15	19	55.56	5	SGGGG
3BVP:B	VYKLDR	77	82	34.87	9	ESSHHH	3OG4:A	RDLKYV	160	165	35.05	10	THHHHH
3BVU:A	DANTR	839	843	42.16	7	CSSEE	1HNJ:A	RTNAD	17	21	43.14	7	EEHHH
3BVU:A	VFVVP	83	87	1.86	10	EEEEE	1ZVT:A	PVVFV	542	546	1.9	8	CEEEE
3BVU:A	MKSIV	149	153	28.9	10	HHHHH	1I7N:B	VISKM	411	415	27.92	8	HHHHH
3BWL:A	TQLSG	459	463	54.14	8	HHHHT	2UUR:A	GSLQT	139	143	54.8	7	SCEEE
3BWV:A	VVKQL	76	80	37.7	8	HHHHH	2XWV:A	LQKVV	230	234	37.4	8	HHHHH
3BWY:A	KKYDV	128	132	90.44	5	HHHCC	2QML:A	VDYKK	54	58	90.18	6	HHHHH
3BWY:A	IVDAV	49	53	19.34	9	HHHHH	3DB2:A	VADVI	309	313	20.22	8	HHHHH
3BWY:A	VVGAS	115	119	24.18	9	EESCH	2G3R:A	SAGVV	1554	1558	24.8	8	EEEEE
3BWY:A	KIVDA	48	52	44.4	9	HHHHH	1R4V:A	ADVIK	143	147	44.08	7	HHHHH
3BWZ:A	LDKTT	11	15	68.94	7	ESCSC	1W9M:A	TTKDL	186	190	69.6	7	TTSCC

3BWZ:A	GTVAV	111	115	12.02	8	EEEEE	2OKG:B	VAVTG	145	149	11.86	6	EEECC
3BWZ:A	YRTAA	101	105	67.42	8	HHHHT	1OD6:A	AATRY	120	124	66.5	6	CCGGG
3BY4:A	NKNQP	236	240	88.76	4	ETTST	1TL2:A	PQNKN	21	25	87.92	2	CCSTT
3BY9:A	LSPKI	281	285	65.08	6	EEECC	2QHK:A	IKPSL	81	85	64.54	4	SCGGG
3BZN:A	LRRCI	319	323	23.5	11	EEEEE	2R9G:P	ICRRL	275	279	23.74	9	HHHHH
3C26:A	VLRVS	63	67	37.9	9	EEEET	3MW4:A	SVRLV	252	256	37.34	7	EEEEC
3C2U:B	YLTTA	104	108	17.1	8	EEEEE	3BEE:B	ATTLY	155	159	17.28	9	HHHHH
3C2U:B	EFSPN	399	403	42.7	7	ECCCS	1W94:A	NPSFE	76	80	42.16	9	EEEEC
3C6A:A	YQFEK	382	386	61	7	EESSC	2GB4:B	KEFQY	171	175	61	7	EEEEE
3C8C:A	EFNGK	238	242	59.46	5	GGTTS	3HCW:A	KGNFE	192	196	58.72	5	SSCCH
3C8C:A	EIVDG	72	76	44.46	9	HHHHH	3SGR:F	GDVIE	6	10	44.2	7	EEEEE
3C9F:A	GGHSH	247	251	7.38	10	ECSSC	2WJ6:D	HSHGG	100	104	7	9	EGGGH
3CB0:A	DAFSG	93	97	22.84	8	HHHTT	3HPW:C	GSFAD	63	67	22.28	8	CCHHH
3CBN:A	RADKA	131	135	53.08	9	SCSCC	3LE0:A	AKDAR	156	160	52.12	9	EEEEE
3CBW:A	GLTSY	202	206	48.84	8	SCSST	2P13:B	YSTLG	469	473	49.76	9	TCBHH
3CBW:A	LSKIA	169	173	21.76	9	HHHHH	3KAE:A	AIKSL	81	85	21.6	11	HHHHH
3CBW:A	YSLFI	304	308	24.68	7	HHHHH	1GKP:E	IFLSY	151	155	25.2	9	EEECS
3CFU:A	KGRIL	151	155	58.92	4	TTTBC	1U7G:A	LIRGK	35	39	59.92	6	TSCHH
3CH0:A	TKGTL	198	202	76.2	5	SSCCH	3U9Q:A	LTGKT	237	241	75.96	5	HTTCS
3CIJ:B	GELDY	228	232	17.16	11	TSCSE	2YHG:A	YDLEG	553	557	18.02	10	SEEEE
3CIJ:B	ELGQE	317	321	57.86	6	HTTCC	3GZY:B	EQGLE	66	70	58.26	8	GGGGS
3CIJ:B	RFGFS	144	148	8.28	13	CEEEE	2GIA:D	SFGFR	165	169	8.3	11	TTCHH
3CJW:A	LFVLN	252	256	0.48	11	HHHHH	2Y6U:A	NLVFL	54	58	0.56	9	EEEEE
3CKM:A	AIVPV	569	573	48.78	6	EEEEC	3LPW:B	VPVIA	191	195	49.56	5	SCEEC
3CLM:A	RLHAA	125	129	55.96	7	HHHHH	3E8O:A	AAHLR	32	36	56.26	7	HHHHH
3CM3:A	PMLYF	1136	1140	61.5	6	HHHHH	2X5P:A	FYLMP	66	70	61.94	7	EEECS
3CNY:A	NNYKG	263	267	76.74	9	TTCCE	3Q7Z:A	GKYNN	534	538	77.04	8	TEEEE
3CP5:A	LRDVT	60	64	33.52	7	CTTHH	2XGT:A	TVDRL	360	364	32.58	7	HHHHH
3CQ0:B	TVKKA	120	124	35.24	10	HHHHH	2IFT:A	AKKVT	76	80	35.86	9	CSEEE
3CQL:B	TGGWP	69	73	24.76	6	CCCCT	2PPL:A	PWGGT	33	37	24.98	5	TTSSS
3CQL:B	NNLDC	232	236	54.88	8	SCCCC	3OIP:A	CDLNN	177	181	55.5	6	THHHH
3CT9:B	LYSNE	253	257	48.96	9	TCCHH	3ZWF:A	ENSYL	162	166	48.44	7	TTBEE
3CTP:A	VKMLE	216	220	67.7	3	GGGGG	1X2I:A	ELMKV	44	48	67.66	5	HHTTS

3CTZ:A	ELQKQ	599	603	73.42	6	HHHHT	1I4D:A	QKQLE	217	221	72.5	7	HHHHC
3CTZ:A	LLTLGL	166	171	44.43	7	CEECCH	2OKG:B	LGLTLL	92	97	44.93	8	HTHHHH
3CU9:A	DEGWP	307	311	65.18	5	TTSCE	1M2X:A	PWGED	86	90	65.46	4	CSSGG
3CYP:B	IIQKL	147	151	46.98	7	HHTTS	2VDU:D	LKQII	347	351	46.08	8	EEEEE
3CZZ:A	SNFIE	52	56	68.4	5	CCGGG	3KBY:A	EIFNS	60	64	68.9	3	HHHTT
3D02:A	VVSFG	217	221	7.96	8	EEESS	3ZWF:A	GFSVV	188	192	7.2	8	EEEEE
3D06:A	IRVEG	195	199	48.82	7	EEEES	2IFT:A	GEVRI	11	15	48.8	8	CEEEC
3D0J:A	SITQK	93	97	45.04	6	EEECT	3BUX:B	KQTIS	127	131	46.02	8	HHHHH
3D1P:A	EPSEY	48	52	54.28	8	CHHHH	3FOT:A	YESPE	108	112	53.62	7	EECCS
3D3S:D	PPLDL	23	27	55.2	3	TTSCC	2PTH:A	LDLPP	95	99	55.56	4	TTSCT
3D59:A	AVIGH	268	272	1.34	13	EEEEE	3S9C:A	HGIVA	210	214	1.86	14	EEEEE
3D59:A	FIVAA	172	176	1.42	9	CEEEE	1FJ2:A	AAVIF	17	21	0.54	8	EEEEE
3D89:A	VDNGN	124	128	73.78	3	EETTE	3VC8:A	NGNDV	70	74	73.84	4	CSCCE
3D9X:A	GVAEG	472	476	29.52	7	EECCC	3HLX:A	GEAVG	101	105	30.02	6	HHHTT
3DAO:A	KEILK	75	79	82.54	4	SCEEE	1JR8:B	KLIEK	63	67	83.04	5	HHHHH
3DAO:A	VKDGS	14	18	70.96	6	BSTTC	3M9Z:A	SGDKV	193	197	70.46	4	ETTEE
3DAS:A	DEATI	51	55	35.38	8	TTCEE	2P0W:A	ITAED	273	277	35.18	8	BEESS
3DAU:A	AEVEG	117	121	59.06	5	CCCCC	3H9W:A	GEVEA	114	118	58.48	6	SCEEE
3DB2:A	VARVF	196	200	16.42	9	EEEEE	3NPH:B	FVRAV	55	59	17.16	10	HHHHH
3DB2:A	DFESV	295	299	80.3	5	CCCCC	2Y9W:D	VSEFD	84	88	81.26	5	ESSCC
3DCZ:A	LKEYK	78	82	85.76	5	EEEET	2BLA:A	KYEKL	68	72	85.64	7	HHHHH
3DDC:B	EDGTY	227	231	69.88	4	GGGCE	1ORU:A	YTGDE	171	175	69.48	4	ETTCE
3DF8:A	DIRSS	44	48	53.3	6	HHHHT	3CT9:B	SSRID	280	284	54.28	6	CEECC
3DHA:A	VVKKE	227	231	86.26	5	HHHHH	2DJF:A	EKKVV	37	41	86.68	4	EEEEE
3DHA:A	EGQIL	74	78	63.5	5	TTTEE	4DOV:A	LIQGE	53	57	62.82	7	EECCS
3DHA:A	LLTID	191	195	0.78	11	EEEET	1YPH:C	DITLL	102	106	1.04	9	CCEEE
3DK9:A	ALGSK	208	212	50.82	10	HTTCE	1XDN:A	KSGLA	74	78	50.16	9	HTTGG
3DK9:A	ALLTP	336	340	53.56	6	SCCHH	3M3P:A	PTLLA	69	73	53.96	5	HHHHH
3DK9:A	MAGIL	202	206	3.1	9	HHHHH	1LYV:A	LIGAM	413	417	2.68	7	HHHHH
3DK9:A	RDAYV	103	107	41	9	HHHHH	3JTW:A	VYADR	70	74	40.98	7	TTTTS
3DK9:A	GLTED	381	385	48.22	9	ECCHH	1RLW:A	DETLG	99	103	47.5	7	CEEEE
3DK9:A	KEVKK	252	256	80.6	8	EEEEE	2EA7:A	KKVEK	391	395	80.1	6	HHHHH
3DLC:A	KKFDK	6	10	95.54	6	CTTSH	3GMI:A	KDFKK	34	38	95.38	6	HHHHH

3DLC:A	GALSI	54	58	10.16	9	SHHHH	3AOW:C	ISLAG	59	63	9.6	7	EECCC
3DLQ:R	SKVRS	130	134	59.6	8	ECSSE	3EYP:B	SRVKS	292	296	60.26	10	TCCCC
3DMO:D	PTLEV	101	105	45.28	9	TTCEE	1X0G:D	VELTP	2	6	44.56	9	CEECH
3DNT:A	GAVTL	99	103	5.64	7	SSCEE	3S46:B	LTVAG	103	107	5.02	6	EEECC
3DNT:A	RYHAK	78	82	69.92	10	HHTCS	3GGY:A	KAHYR	52	56	70.9	9	HHHHH
3DQY:A	GDEVH	93	97	46.7	7	TTEEE	1PZX:A	HVEDG	193	197	46.5	7	EEETT
3DRA:A	TQLEE	238	242	74.92	5	GGGHH	3KP1:F	EELQT	20	24	74.5	6	HHHHH
3DRF:A	QDLDK	379	383	73.76	8	CCHHH	2CVI:A	KDLDQ	50	54	74.74	6	HHHHH
3DTZ:B	AYSDA	18	22	68.3	6	CCCHH	2GQ0:A	ADSYA	435	439	68.76	6	CSSHH
3DXY:A	VGACL	100	104	7.54	9	HHHHH	2CF5:A	LCAGV	162	166	8.24	8	GTHHH
3E10:B	EKIEK	21	25	61.84	8	HHHHH	3NO6:A	KEIKE	195	199	62.06	10	HHHHH
3E3M:D	RLKSI	266	270	71.06	7	HHHHH	1G5H:C	ISKLR	442	446	70.06	7	GGGHH
3E3U:A	ARVTG	122	126	26.9	8	EEEEE	1YU0:A	GTVRA	117	121	26.8	7	SCEEE
3E8T:A	ALPTF	23	27	44.5	6	HHHHH	1F3U:B	FTPLA	146	150	45.16	6	EEEGG
3E9V:A	EQRLK	33	37	72.36	7	HHHHH	3H11:A	KLRQE	430	434	72.86	8	HHHHC
3EA6:A	ISESK	45	49	34.78	11	EEECS	1LTZ:A	KSESI	205	209	34.6	9	HHHHH
3EA6:A	ISESK	45	49	34.78	11	EEECS	1LTZ:A	KSESI	205	209	34.6	9	HHHHH
3EBT:A	KFVHV	101	105	46.38	9	EEEEE	3DSO:A	VHVFK	18	22	45.54	11	EEEET
3EBT:A	VRESY	8	12	55.8	10	HHHHH	3LL8:A	YSERV	170	174	55.5	8	SCHHH
3ED1:C	LPEDA	259	263	75.72	4	SCTTC	3RH3:B	ADEPL	181	185	76.46	3	TTCTT
3EDV:B	DAATI	1772	1776	41.88	7	THHHH	3BL4:A	ITAAD	31	35	42.84	5	CCHHH
3EE4:A	VRASV	156	160	17.7	9	HHHHH	1F00:I	VSARV	732	736	17.84	10	EEEEE
3EEI:A	KTAAA	216	220	45.1	6	HHHHH	3PDY:B	AAATK	650	654	44.74	8	HHHHH
3EF4:C	GFVKV	22	26	33.86	6	SEEEE	3LFJ:B	VKVFG	58	62	34.76	6	EEEEC
3EG3:A	VLGYN	90	94	60.78	8	EEEEC	2D1S:A	NYGLV	71	75	60.38	8	HHTCC
3ELK:B	ITDAG	81	85	44.52	7	ECHHH	3ANU:A	GADTI	185	189	44.78	7	SHHHH
3EN0:C	QPAIL	6	10	34.6	9	CCCEE	1UI0:A	LIAPQ	111	115	34.06	8	HHCCS
3ENU:A	FLSGPA	65	70	35.18	6	EEESSE	1V8D:C	APGSLF	61	66	35.98	7	CTTCEE
3EO6:A	SRISL	16	20	56.38	10	CEEET	3OII:A	LSIRS	148	152	56.66	8	SEEEE
3EP6:B	DLRTI	31	35	34.06	6	CGGGS	3QC2:B	ITRLD	321	325	34.08	8	EEECS
3EPW:A	ATVRT	293	297	44.28	8	CEEEC	1T0H:B	TRVTA	264	268	45.04	7	EEECS
3EPW:A	LTAAY	263	267	1.4	7	HHHHH	3MEZ:A	YAATL	87	91	1.62	9	CEEEE
3EQA:A	DGEQLS	430	435	56.3	8	TCCEES	2RE9:C	SLQEGD	148	153	56	7	EECTTC

3ERJ:B	GIKHK	63	67	63	7	HHHHH	2O16:A	KHKIG	122	126	63.64	7	HTTCS
3ERJ:B	ITAVV	91	95	7.24	8	CCEEE	1TOJ:A	VVATI	305	309	6.54	7	HHHHH
3ESL:A	TKIAY	39	43	53.6	9	HHHHH	2ZXE:B	YAIKT	83	87	53.78	7	CCSSS
3ESL:A	GQEVL	80	84	59.78	8	HHHHH	2OEG:A	LVEQG	207	211	60.74	6	HHHTT
3ESS:A	HAEVA	497	501	40.8	4	SHHHH	1U9D:B	AVEAH	8	12	40.74	6	SSCHH
3EUL:D	PDVAL	55	59	69.54	5	CCCCE	2V6K:A	LAVDP	145	149	69.78	5	HHHCT
3EY6:A	AMVTA	112	116	15.46	9	EEEEE	3V5U:A	ATVMA	43	47	16.3	9	HTHHH
3EY6:A	VIQAL	95	99	33.42	6	SCHHH	2XHG:A	LAQIV	277	281	34.38	8	CSEEE
3EYE:A	TVRKL	91	95	38.58	7	HHHHH	2OSA:A	LKRVT	401	405	38.4	8	HHHHH
3F0D:D	DSTII	97	101	0.34	10	EEEEE	2VDU:D	IITSD	213	217	1.04	11	EEEEE
3F0H:A	VERVE	205	209	70.78	7	HHHHH	1BJA:A	EVREV	36	40	70.36	6	HHHHT
3F5H:B	ALRAS	11	15	57.12	9	HHHHH	1H0H:L	SARLA	174	178	56.42	9	HHHHH
3F65:E	ALLEGR	115	120	50.43	6	GGTTTC	3EAT:X	RGELLA	222	227	50.6	6	HHHHHH
3F8X:A	VEVGK	136	140	62.14	6	HHHHH	1H72:C	KGVEV	295	299	62.8	4	CCCEE
3F95:A	LADTS	650	654	39.34	8	EEESS	2RDE:B	STDAL	29	33	39.84	8	HHHHH
3F9T:B	LGSLL	77	81	9.5	7	HHHHT	3B1F:A	LLSGL	165	169	9.76	7	HTGGG
3FA2:A	NLIKLVT	690	696	42.357	6	HHHHHHH	3RYD:A	TVLKILN	250	256	42.3	5	HHHHHHH
3FCI:A	GLVFL	239	243	1.34	11	SCEEE	1V8D:C	LFVLG	65	69	0.42	11	EEEEE
3FCN:A	VARLG	156	160	72.38	5	HHHTC	1DQT:A	GLRAV	81	85	72.96	6	SCCGG
3FCN:A	ATSLV	57	61	12.36	8	HHHHH	3FSA:A	VLSTA	49	53	11.64	10	EEEEH
3FDJ:A	AKVRV	105	109	34.24	10	CEEEE	1H72:C	VRVKA	7	11	33.9	9	EEEEE
3FET:D	FLTVS	3	7	3	12	EEEEE	2Y1K:A	SVTLF	191	195	2.38	13	EEEEE
3FGV:A	FLEIN	86	90	52.24	8	HHHGG	3EPZ:B	NIELF	430	434	53	7	TCCCE
3FGV:A	KLVSE	36	40	54.42	6	HHHHH	2HQT:H	ESVLK	34	38	54.52	5	HHHHH
3FLE:B	SHSDG	232	236	31.54	8	SSBSS	2VYN:D	GDSHS	285	289	30.96	7	TCCCS
3FO3:B	LSQTA	173	177	64.4	8	STTCC	1SVF:C	ATQSL	157	161	65.28	8	HHHHH
3FOT:A	AAIVLA	312	317	0.62	9	HHHHHH	2ANU:A	ALVIAA	134	139	1.22	10	CEEEEC
3FOT:A	RALES	384	388	47.04	8	HHHHH	3TGN:A	SELAR	55	59	47.2	6	HHHHH
3FRH:A	IACGL	110	114	8.96	7	ETCTT	2WCJ:A	LGCAI	48	52	8.94	8	HHHHH
3FRH:A	RRILT	25	29	41.16	11	HHHHH	2FTB:A	TLIRR	117	121	40.64	9	EEEEE
3FTD:A	FVPPP	166	170	60.52	5	EESCC	2V1T:A	PPPVF	115	119	61.18	4	CHHHH
3FVV:A	VELAA	75	79	27.9	5	HHHHH	2J9O:D	AALEV	110	114	27.1	7	EHHHH
3FWK:A	LVDDK	290	294	64.66	6	CCCGG	1I07:A	KDDVL	27	31	65.2	5	TTCEE

3FWK:A	LENFI	117	121	17.6	8	HHHHH	3KAL:A	IFNEL	99	103	16.86	10	HHHHH
3FWK:A	IEETS	121	125	31.58	10	HHHHH	3BGY:A	STEEI	146	150	31.76	8	EEEEE
3FXH:A	SNALLL	93	98	34.9	7	SCHHHH	3HRG:A	LLLANS	181	186	34.42	5	EEEEEE
3FYN:A	KALLGK	41	46	68.45	7	HHHHHC	3E1R:B	KGLLAK	196	201	68.77	8	HHHHHH
3FYN:A	VLTLG	66	70	18.54	9	EEEEE	1QVY:A	GLTLV	74	78	18.78	9	EEEEE
3FYN:A	ALPHD	30	34	78.42	5	CCCHH	3F6C:B	DHPLA	8	12	79.16	5	CCHHH
3FZ4:A	DEAAN	79	83	55.94	6	HHHHH	3DQG:A	NAAED	547	551	56.48	6	HHHHH
3FZW:A	ALDVI	98	102	9.4	10	EEEEE	3KOP:E	IVDLA	107	111	8.76	8	EEEEE
3G3L:A	EKKAL	180	184	63.98	7	HHHHH	2P0L:A	LAKKE	72	76	63.5	6	HHHHH
3G3L:A	ITPVK	212	216	47.78	8	EEEEE	3KF8:D	KVPTI	46	50	47.06	6	ECSSS
3G7R:B	LRDVS	191	195	58.14	7	HHHHH	3L77:A	SVDRL	34	38	58.54	8	CHHHH
3G7R:B	LLAQV	144	148	39.1	6	HHHTT	1S9U:A	VQALL	34	38	39.9	6	HHHHH
3G91:A	LARPK	161	165	72.06	5	BSCTG	1S5U:H	KPRAL	120	124	72.28	7	CBCCC
3GA7:A	DDALQ	305	309	30.02	9	HHHHH	3OIS:D	QLADD	278	282	29.56	11	TTEEE
3GBW:A	LGGLG	1264	1268	5.4	7	EEEEE	1JW9:B	GLGGL	38	42	5.04	9	CCSHH
3GBW:A	GPDGG	1286	1290	37.28	5	ETTTT	3NQA:B	GGDPG	186	190	38.26	7	SBCHH
3GDC:C	FIVEP	161	165	6.84	11	EEEEC	3NO8:B	PEVIF	519	523	7.2	10	CEEEE
3GE3:B	CILQT	152	156	2.44	10	HHHHH	3P8K:A	TQLIC	142	146	2.88	11	EEEEG
3GGY:A	KEIAK	174	178	74.32	8	HHHHH	2IY2:A	KAIEK	8	12	73.92	7	HHHHT
3GI7:A	LRDAD	90	94	22.42	9	HHHHH	2QGS:A	DADRL	121	125	22.02	9	HHHHH
3GI7:A	YLRSL	127	131	41.98	9	HHHHH	1JKG:A	LSRLY	35	39	41.58	7	GGGGE
3GJU:A	GVIGR	416	420	31.04	7	TEECE	1LO7:A	RGIVG	54	58	30.52	7	HCEEE
3GJU:A	ANLEL	339	343	27.48	11	HHHHH	2AGK:A	LELNA	158	162	27.14	9	EEESH
3GM5:A	KNGEG	91	95	75.34	6	HHCSE	1CFR:A	GEGNK	8	12	75.3	7	SSTTB
3GMI:A	EKVID	156	160	63.32	7	HHHHH	2B4A:A	DIVKE	68	72	63.92	5	HHHTT
3GMO:A	VAFQG	118	122	26.96	7	EEETT	3BB6:A	GQFAV	72	76	27.52	8	TBEEE
3GNE:B	PKNQV	228	232	60.8	7	SSCEE	3GJ3:B	VQNKP	732	736	61.52	8	CEECT
3GNZ:P	KSYLD	139	143	79.92	6	GGGEE	3C26:A	DLYSK	33	37	79.28	7	HHHHH
3GOD:A	LSRAQ	300	304	59.9	8	HHHTT	3KK4:A	QARSL	16	20	59.68	9	EEEEE
3GOD:D	LSRAQ	300	304	42.14	9	HHHTT	3KK4:C	QARSL	16	20	42.84	10	EEEEE
3GSZ:B	EDLLE	124	128	76.94	6	HHHHH	1PZX:A	ELLDE	104	108	76.18	7	HHHHH
3GSZ:B	SVLQD	66	70	50.32	8	HHHHH	3D7R:B	DQLVS	135	139	50.52	7	HHHHH
3GSZ:B	LTVEE	83	87	50.02	6	CCHHH	3PZ7:A	EEVTL	412	416	50.02	8	TTCBH

3GV3:A	ANVKH	21	25	61.46	8	GGEEE	3NUQ:A	HKVNA	118	122	61.58	9	TSSCH
3GVO:A	QQKLE	745	749	61.78	9	HHHHH	2YXT:A	ELKQQ	100	104	60.78	11	HHHHH
3GX8:A	GELAD	127	131	56.76	6	THHHH	2QKV:B	DALEG	635	639	56.54	5	EECTT
3GZ7:A	SILVQ	7	11	31.34	10	EEEBC	1AXD:B	QVLIS	116	120	30.86	12	HHTHH
3GZR:B	SALAV	110	114	0.5	8	EEEEE	3L4R:A	VALAS	22	26	0.76	8	EEEEE
3H09:B	IYKKGD	211	216	61.45	7	EEEEEE	3S5B:A	DGKKYI	216	221	61.63	8	TSCEEE
3H09:B	TKNEL	942	946	72.64	7	CSCCE	1SLU:A	LENKT	59	63	72.58	6	EEEEE
3H09:B	KLRNV	976	980	74.62	7	EEEEE	3BDW:D	VNRLK	213	217	74.8	6	SSSEE
3H09:B	YKPEF	325	329	51.18	8	CCHHH	2ZXE:B	FEPKY	65	69	50.2	6	SSCSC
3H2D:B	GAKNL	99	103	50.6	7	HHHHH	3PIS:A	LNKAG	23	27	49.98	6	HHHTT
3H3L:A	DTGFG	93	97	67.4	5	CCSCC	1NOW:A	GFGTD	375	379	67.38	5	TCTTC
3H4T:A	AIEGC	89	93	43.6	5	HHTTC	3H87:A	CGEIA	50	54	44.44	5	SHHHH
3H5J:B	FAGWR	43	47	42.6	7	TTTGG	1YB0:A	RWGAF	146	150	43.1	7	CHHHH
3H5Z:A	IPAQY	235	239	71.32	4	CCGGG	3CZ8:A	YQAPI	345	349	71.9	5	TTCCC
3H7H:A	SFDGI	56	60	19.34	10	CEEEE	2GNP:A	IGDFS	210	214	18.44	10	CEECS
3H7H:A	QFEYD	27	31	56.04	9	HHHHH	1CNU:A	DYEFQ	66	70	56.54	7	EEEEE
3H7I:A	EESTW	95	99	78.38	7	HHCSS	1W9H:A	WTSEE	355	359	79.28	6	CCTTT
3H9M:A	AVAGS	290	294	56.22	3	BCTTC	3B1F:A	SGAVA	131	135	56.5	4	CCTTS
3H9M:A	LIHKGS	127	132	47.12	8	EEEESS	1Q33:A	SGKHIL	192	197	47.5	8	TCSBCE
3H9M:A	ETEYE	408	412	54.68	9	HHHHH	2X61:A	EYETE	70	74	55.18	11	SCEEE
3HA2:A	LPILA	137	141	48.4	6	CCCEE	1PZX:A	ALIPL	198	202	49	6	EEEEE
3HA4:F	NVKYG	148	152	58.7	7	HHHTT	3AKH:A	GYKVN	191	195	58	7	SSCBE
3HFO:C	TDGLGL	43	48	35.72	7	SSEEEE	4DNU:A	LGLGDT	152	157	35.68	6	TCSSSC
3HHT:A	IGVAK	203	207	25.5	6	TTSCC	1JMV:A	KAVGI	21	25	25.46	8	HHHHH
3HHT:B	GLSPL	129	133	41.96	6	CCCCE	2QKL:B	LPSLG	56	60	41.66	7	SCCCC
3HHT:B	IELMR	59	63	42.78	10	HHTSC	2Q0I:A	RMLEI	288	292	42.04	9	HHHHH
3HIM:A	IEAAA	158	162	28.14	8	HHHHH	2PKF:B	AAAEI	315	319	28.4	7	HHHHH
3HIN:A	GGLEL	120	124	4.8	9	HHHHH	1O04:G	LELGG	267	271	4.08	8	EECCC
3HLX:A	DSAEK	199	203	65.96	7	CSHHH	3AA0:A	KEASD	103	107	65	6	TEEEE
3HM2:H	LTKQH	5	9	58.68	7	HHHHH	2Z0U:A	HQKTL	103	107	58.88	8	HHCEE
3HNA:B	RLQLY	1096	1100	26.68	9	CEEEE	1RY9:D	YLQLR	97	101	27.54	10	EEEEE
3HP4:A	VKLLQ	25	29	34.98	9	HHHHH	1MAI:A	QLLKV	27	31	35.46	8	EEEEE
3HPC:X	DKVKF	43	47	55.6	7	TEEEE	1C44:A	FKVKD	37	41	56	8	EEEES

3HQC:A	KPGSP	1372	1376	66.22	3	ETTEE	3LLR:D	PSGPK	142	146	66.2	4	TTGGG
3HRG:A	IDKST	122	126	42.68	8	EEHHH	1XG8:A	TSKDI	18	22	43.04	7	CHHHH
3HTN:A	EIVKAL	63	68	37.6	7	BHHHHH	2P1O:A	LAKVIE	52	57	38.35	7	HHHHHH
3HTN:A	SALAG	142	146	11.6	8	BEEEE	3S6L:D	GALAS	94	98	12.42	7	TCEEC
3HXW:A	GVMPS	297	301	43.36	6	TCCCC	1BDO:A	SPMVG	85	89	43.84	6	CSSSE
3HXW:A	LLFTN	36	40	16	9	CSSCC	3P1V:B	NTFLL	113	117	15.52	7	EEEEE
3HZB:H	ILYQD	50	54	35.82	11	EEEEE	3OIP:A	DQYLI	68	72	36.62	9	CSEEE
3HZS:A	SAILA	216	220	2.34	7	HHHHH	1KCF:B	ALIAS	232	236	2.86	8	HHHHH
3HZS:A	VDELR	70	74	44.54	9	GGGGG	2QEA:A	RLEDV	11	15	43.56	8	HHTTC
3I10:A	ILEKT	158	162	59.22	7	HHHHH	2D1S:A	TKELI	410	414	59.5	7	HHHHB
3IAR:A	GLQEG	136	140	27.44	9	HHHHH	3HNA:B	GEQLG	1204	1208	27.74	11	TCBCE
3IB7:A	IIVLD	129	133	1.8	11	EEECC	1M3S:A	DLVII	80	84	1.66	9	CEEEE
3IEZ:B	EMEKV	1529	1533	76.16	6	EEEEE	2FL7:A	VKEME	196	200	77.02	7	HHHSC
3IFW:A	EQGEV	208	212	65.42	5	STTCC	3EJJ:X	VEGQE	118	122	64.84	6	ETTSC
3IGS:B	GGLIV	15	19	1.78	10	CCEEE	3BI1:A	VILGG	372	376	1.44	10	EEEEE
3IGS:B	EQLDK	5	9	69.12	10	HHHHH	1D8D:A	KDLQE	146	150	69.38	8	CCHHH
3IIS:M	SKITD	112	116	70.38	8	HHHSC	3BNW:B	DTIKS	130	134	69.38	9	HHHHH
3IJD:B	LAKDI	295	299	32.36	10	HHHHH	2O1M:B	IDKAL	220	224	31.84	11	HHHHH
3IJW:B	APIIE	199	203	37.7	6	EEEEE	1O7Z:A	EIIPA	28	32	37.64	5	EEECC
3IMH:A	KLHAR	141	145	45.56	9	EEEEE	3AAI:B	RAHLK	64	68	45.1	8	HHHHH
3IMM:A	DKVVK	134	138	61.8	5	SCCSC	1GZS:B	KVVKD	81	85	61.4	6	HHHHH
3IMM:A	DEYEN	65	69	73	7	SCBCS	2WNO:A	NEYED	153	157	73.7	8	SCCCT
3IOF:A	LPIKT	254	258	62.74	5	CCCSE	2F15:A	TKIPL	101	105	62.44	6	CCEEC
3IOF:A	FPDEQ	205	215	38.14	5	ETTTT	1TXJ:A	QEDPF	20	24	39.1	7	CBCGG
3IQ2:A	WLKGK	79	83	49.38	8	HHHHH	3CX5:I	KGKLW	45	49	50.34	10	TTTSH
3IQU:A	AISLA	190	194	24.16	9	HHHHH	1MB3:A	ALSIA	38	42	23.42	8	HHHHH
3IUW:A	FKAVK	18	22	51.86	8	HHHHH	3ACZ:A	KVAKF	268	272	51.9	7	HHHHH
3IVE:A	DTVSA	174	178	17.16	10	HHSCG	2IC2:B	ASVTD	535	539	17.6	8	EEECS
3IVV:A	KFSIL	103	107	9.78	9	EEEEE	3P02:A	LISFK	33	37	10.76	9	SCCCC
3IVV:A	LDEESK	76	81	73.25	6	SSGGGT	2QG6:A	KSEEDL	172	177	73.02	8	SCHHHH
3JQL:A	NIDLK	112	116	84.44	6	TCCHH	3FOT:A	KLDIN	238	242	84.78	4	SSCGG
3JST:B	AEKLD	53	57	89.1	6	HHHHT	3C2E:A	DLKEA	39	43	88.4	6	CEEEE
3JTW:A	LVQAN	124	128	36.1	6	HHHTT	2NTX:A	NAQVL	197	201	36.38	8	HHHHH

3JU4:A	LGLIP	517	521	45.16	4	CCSCT	1TOJ:A	PILGL	16	20	45	5	TTHHH
3JU4:A	DNSAK	731	735	55.92	8	TTTTC	2RIK:A	KASND	262	266	55.62	10	EEEET
3JUD:A	PLVIA	41	45	5.02	6	CEEEE	1LUC:A	AIVLP	75	79	5.28	8	EEEGG
3JVM:A	KKHAA	368	372	77.6	5	GGGHH	1JIX:A	AAHKK	146	150	76.94	5	HHTTT
3K12:A	ILENI	45	49	21.7	9	HHHHH	1D9C:A	INELI	110	114	22	11	HHTHH
3K1S:I	QLILN	11	15	28.16	9	HHHHH	3AJM:A	NLILQ	202	206	28.42	9	HHHHH
3K7I:B	VYYES	178	182	31.26	9	EEEEE	2YXM:A	SEYYV	76	80	31.44	10	EEEEE
3KB2:B	KLADE	51	55	64.02	7	HHHTC	2AUA:B	EDALK	114	118	63.68	6	HHHHH
3KEV:A	EIEDP	90	94	67.3	8	HTTSH	2YWW:A	PDEIE	99	103	68.18	8	CSEEE
3KH1:B	ETEGG	150	154	53.04	7	HTTST	3DB2:A	GGETE	316	320	52.76	7	CCCCS
3KHF:B	GESVL	1008	1012	56.84	7	TEECT	3FTD:A	LVSEG	13	17	56.3	6	EECHH
3KNB:A	LTTLI	62	66	19.56	9	EEEEE	3U7Q:D	ILTTL	493	497	18.58	8	HHHHH
3KOS:A	YTIRY	143	147	23.18	10	EEEEE	1FNF:A	YRITY	1447	1451	22.28	12	EEEEE
3KT7:A	FQTEA	425	429	58	7	CCSHH	3NW4:A	AETQF	339	343	59	7	ESSSE
3KT7:A	KKETD	82	86	62.96	9	EEECS	2FUR:A	DTEKK	115	119	63.1	8	CHHHH
3KT7:A	HDDVI	159	163	43.44	8	ECCCC	2QSJ:A	IVDDH	6	10	43.46	10	EECSC
3KTA:B	PEDPF	1044	1048	63	3	SSSGG	1JR8:B	FPDEP	29	33	63.38	3	SCSSC
3KTA:B	AKLSP	1029	1033	53.04	7	HHHST	1LST:A	PSLKA	57	61	53	6	HHHHT
3KWS:B	KGFIL	113	117	43.2	6	CSCTT	1TVD:A	LIFGK	104	108	42.88	6	EEECC
3KWS:B	RVNEI	90	94	49.08	8	HHHHH	1YB0:A	IENVR	123	127	48.38	6	GGGEE
3KX6:D	QSESM	273	277	52.84	6	CCHHH	3MDY:A	MSESQ	494	498	52.76	7	HHHTT
3KYZ:A	TWVAL	79	83	13.34	8	SCEEE	2J43:B	LAVWT	133	137	12.56	9	EEEEE
3L0A:A	AIVDL	139	143	11.74	8	EEEEE	2HY7:A	LDVIA	179	183	11.54	10	CSEEE
3L0F:A	AVDGR	26	30	43.3	8	HHHHH	1I4D:A	RGDVA	180	184	43.06	8	HHHHH
3L1N:A	EPLVQ	101	105	91.34	5	HHHHH	2NPT:A	QVLPE	50	54	91.46	5	HHSTT
3L32:A	NVGVQ	99	103	43.92	9	HHHHH	3KT9:A	QVGVN	60	64	44.82	7	ECSSS
3L41:A	EDYVV	773	777	56	7	TTSEE	3C6K:C	VVYDE	141	145	56.96	7	EEEEE
3L46:A	THLVV	278	282	6.4	11	SEEEE	1Y63:A	VVLHT	105	109	6.54	9	EEEEC
3L4Q:D	KSREY	450	454	64	9	HHHHH	4A34:T	YERSK	112	116	64.04	7	HHHHH
3L4R:A	GGLEEL	10	15	55.57	4	TTGGGG	3OAB:C	LEELGG	240	245	55.08	4	HHHTTT
3L5W:J	LRELI	10	14	26.9	9	HHHHH	3U7Q:D	ILERL	501	505	27.84	11	HHHHH
3L77:A	ELMLR	222	226	55.68	8	EEEEC	3OLJ:A	RLMLE	330	334	55.98	9	HHHHH
3LBE:A	VSIST	58	62	10.38	7	HHHHT	3KLQ:B	TSISV	7	11	10.3	8	ECCEE

3LF5:B	RAAGS	98	102	22.44	8	TTTTE	2WZV:B	SGAAR	66	70	22.64	10	CHHHH
3LFP:A	LTWNE	76	80	76.76	6	HHHTT	2IFT:A	ENWTL	173	177	76.06	5	TTEEE
3LHQ:A	EIANA	36	40	52.48	8	HHHHH	3G14:A	ANAIE	60	64	51.58	8	HHHBC
3LJK:A	DDSSD	517	521	70.64	6	CTTSH	2VIF:A	DSSDD	410	414	70.5	6	ECSST
3LJK:A	VKELV	117	121	37.42	8	HHHHH	1QGQ:A	VLEKV	168	172	37.18	6	HHHHH
3LKK:A	NPDAV	176	180	73.54	5	CTTCC	2GVG:A	VADPN	418	422	73.28	4	TTCGG
3LLP:A	RHDGR	197	201	63.38	6	CTTSC	2GEF:A	RGDHR	608	612	63.5	8	ESCCG
3LQ9:A	GKELL	128	132	46.24	8	HHHHH	3R24:A	LLEKG	274	278	46.88	7	HHHTT
3LQW:A	TRGSK	18	22	83.62	5	EECST	3O2Q:E	KSGRT	182	186	83.3	6	HHCCC
3LRU:A	ILRAL	1939	1943	25.28	7	HHHHH	1VGW:E	LARLI	120	124	25.88	6	HHHHH
3LVY:D	EINRR	53	57	62.14	7	HHHTT	1KNQ:B	RRNIE	45	49	62.06	8	HHHHH
3LW6:A	LLVRR	187	191	13.64	13	EEEEH	1D8D:A	RRVLL	137	141	13.5	11	HHHHH
3LX3:A	SESPS	159	163	55.6	3	EEETT	3KGR:A	SPSES	78	82	55.76	5	ETTEE
3LYE:A	MIANLD	141	146	36	8	HHHTSS	1TUL:A	DLNAIM	82	87	35.85	8	CCEEEE
3LYE:A	KVVSR	199	203	63.62	10	CBCCH	3S83:A	RSVVK	487	491	63.74	8	HHHHH
3LYH:B	IILLA	7	11	3.76	8	EEEEE	3QBM:A	ALLII	157	161	3.2	7	HHHHH
3LYH:B	DTIVN	50	54	39.86	10	HHHHH	2SAS:A	NVITD	146	150	39.24	8	HHHHT
3M1I:B	DIAEG	163	167	38.54	6	ECTTS	1KZL:A	GEAID	116	120	38.4	5	TTEEE
3M1X:A	VLEEA	63	67	40.16	6	HHHHT	2NLV:A	AEELV	80	84	40.44	8	HHHHH
3M5Q:A	KVDQT	180	184	80.08	5	SSSTT	3Q6A:H	TQDVK	99	103	79.66	6	SSCHH
3M7A:B	GLDRR	115	119	59.54	6	CTTSB	3R5G:B	RRDLG	412	416	59.12	6	TTCBC
3M7A:B	TPLPAA	133	138	52.43	7	SCEECS	3FDR:A	AAPLPT	35	40	52.6	6	EEEETT
3MB2:F	SGDRP	8	12	90.7	4	CSSSC	3M0F:A	PRDGS	151	155	90.26	3	CCSSC
3MBK:B	PPSEL	505	509	48.78	5	CHHHH	2NXY:B	LESPP	1118	1122	48.12	6	EECCT
3MBK:B	QARLV	437	441	42.94	11	HHHHH	1YMT:A	VLRAQ	349	353	43.62	10	HHHHH
3MBR:X	VTAGG	163	167	34.6	6	CEETT	3MUJ:B	GGATV	268	272	34.32	7	CCCEE
3MBR:X	RLIQL	97	101	11.3	11	EEEEE	2Z80:A	LQILR	151	155	11.04	9	CCEEE
3MD9:A	KVLFV	160	164	20.24	8	EEEEE	2HKX:B	VFLVK	49	53	19.72	9	EEEEE
3MDY:A	EQSQS	181	185	68.22	8	HHHHH	1VF6:D	SQSQE	141	145	68.12	8	HHHHH
3ME5:A	QHRER	274	278	56.54	11	BCCEE	2H09:A	RERHQ	106	110	57.36	11	HHHHH
3MGD:A	IDEGI	24	28	51.98	8	HHTTC	2PNL:J	IGEDI	592	596	51.56	8	BCSCB
3MGD:A	NKLAN	44	48	73.32	6	HHHHT	3FYF:A	NALKN	59	63	72.48	6	HHHHT
3MH9:C	LTVNG	62	66	45.9	7	EEEES	1N67:A	GNVTL	348	352	46.28	6	EEEEE

3MHP:C	DLKPP	14	18	36.78	4	TSSCS	1FYH:E	PPKLD	112	116	37.56	4	CCEEE
3MJO:A	ILSSL	292	296	36.54	5	HHHHH	1KZQ:A	LSSLI	69	73	37.06	4	GGGTS
3MK1:A	ACLEP	466	470	48.66	6	TTCTT	3KBR:A	PELCA	207	211	48.6	5	TTEEE
3MK1:A	DKLGP	61	65	74.24	5	TSCST	1QSA:A	PGLKD	30	34	74.9	3	GGGTT
3MK6:C	LKSIR	45	49	49.28	8	HHHHH	3BI1:A	RISKL	511	515	49.36	6	CCBCC
3MM1:A	TLANA	234	238	44.2	6	HHHTC	2OXC:A	ANALT	251	255	45.18	7	HHHHT
3MM1:A	VVPPG	191	195	23.46	8	EECGG	1IMJ:A	GPPVV	103	107	24.46	7	CSCEE
3MMH:A	VLDADS	130	135	4.55	12	EEEEEE	1GKP:E	SDADLV	388	393	4.85	13	SBCCEE
3MNL:A	ARVIS	157	161	14.78	8	HHHHH	1BD3:B	SIVRA	109	113	14.36	8	EEETG
3MOE:A	NVAET	359	363	4.42	13	SCEEE	1Q33:A	TEAVN	282	286	4.84	14	EEEEE
3MOL:B	KLDSI	88	92	31.5	8	EEEEE	1U02:A	ISDLK	32	36	31.14	7	HHHHH
3MQ2:A	WKLAD	173	177	52.08	8	EEEEE	2AUA:B	DALKW	115	119	51.66	8	HHHHH
3MQ2:A	LVALN	134	138	8.82	10	EEEEE	1RZ4:A	NLAVL	47	51	9.42	9	HHHHH
3MQ2:A	DVGTG	30	34	12.82	8	EESCT	3THR:D	GTGVD	66	70	13.62	10	TTSHH
3MQD:A	CIGNA	167	171	5.2	9	HHHHH	3QJG:L	ANGIC	91	95	6.04	9	HHTCC
3MQH:B	AVVNK	131	135	39.5	8	CEECS	3QJG:L	KNVVA	158	162	39.44	7	EEECC
3MST:A	SALVG	114	118	1.7	9	EEEEE	1KQ3:A	GVLAS	276	280	2.06	8	HHHHH
3MSX:B	NDNLP	373	377	60.74	6	GGCCC	3LW6:A	PLNDN	150	154	60.2	7	ECCTT
3MZ0:A	DSLLA	59	63	42.28	5	HHHHH	3I26:B	ALLSD	169	173	41.6	5	EECSS
3MZ0:A	KEKVE	324	328	88.66	7	CEECC	1N81:A	EVKEK	145	149	89.42	7	SHHHH
3MZ0:A	QVIYP	193	197	55.4	5	EEECC	3ALN:C	PYIVQ	159	163	56.34	7	TTBCC
3N0R:A	LIGDA	127	131	27.1	8	HHHHH	2GNP:A	ADGIL	184	188	26.74	7	HHHHH
3N3M:A	LCIGL	18	22	0.1	9	EEEEC	3H11:A	LGICL	251	255	0.34	10	SEEEE
3N3M:A	AFVLV	188	192	0.82	8	EEEEE	3HUL:B	VLVFA	249	253	0.28	8	EEEEE
3N3M:A	KYALT	96	100	26.5	9	GGCSE	2GEF:A	TLAYK	567	571	25.58	8	EEEEE
3N3M:A	FEYLK	154	158	40.22	7	HTTSC	2R4G:A	KLYEF	404	408	41.16	9	HHHHH
3N6Y:A	LTLLY	89	93	12.26	8	EEEEE	1W9H:A	YLLTL	323	327	12.4	9	EEECC
3N6Y:A	DKFEV	107	111	36.76	10	EEEEE	3FLE:B	VEFKD	105	109	36.74	9	EEESS
3N72:B	EDLTI	42	46	47.06	7	SSEEE	2GN4:A	ITLDE	208	212	46.98	8	ECHHH
3N8B:A	SNLLKA	69	74	55.67	8	HHHHHH	3PDY:B	AKLLNS	629	634	55.47	9	HHHHHH
3NBC:B	DANST	120	124	55.42	6	CSTTC	1QVE:A	TSNAD	130	134	55.46	6	EESSC
3NBC:B	YVRVR	70	74	38.62	9	EEEEG	3CJJ:A	RVRVY	114	118	39.28	11	EEEEE
3NDI:A	GDIVA	210	214	28.48	7	HHHHH	2PC1:A	AVIDG	68	72	28.6	7	EEEEE

3NE8:A	KAQEFD	244	249	70.35	6	HHHHTT	3EHC:C	DFEQAK	104	109	69.53	7	EEETTE
3NE8:A	FDADL	248	252	40.76	8	TTCSE	2WJ6:D	LDADF	50	54	41.3	7	HTTTS
3NED:A	TKLDI	197	201	44.94	8	EEEEE	3NCE:B	IDLKT	133	137	44.2	8	CCCTT
3NED:A	GVVTVT	103	108	19.97	10	EEEEEE	3ON9:B	TVTVVG	269	274	20.02	10	EEEEES
3NFW:A	QRTFR	8	12	65.44	9	HHHHH	3IRS:A	RFTRQ	29	33	64.56	9	HHHHH
3NKE:B	VHTGK	207	211	47.92	8	SSCSS	1GR3:A	KGTHV	606	610	47.48	8	EEEEE
3NKE:B	ADIIK	220	224	20.26	7	HHHHT	3PQS:A	KIIDA	294	298	19.92	9	ECEEE
3NPF:B	YEKPD	116	120	75.24	7	ESSSS	3D6I:B	DPKEY	132	136	75.48	5	CHHHH
3NPK:A	IAAAL	177	181	2.56	6	HHHHH	3OIS:D	LAAAI	83	87	2.16	7	HHHHH
3NRH:A	VLRIN	78	82	52.78	8	HHHHH	1ZCB:A	NIRLV	354	358	52.38	8	HHHHH
3NSW:G	TVAYVT	20	25	18	10	EEEEEE	1FNF:A	TVYAVT	1486	1491	18.22	12	EEEEEC
3NTV:A	AKAQS	151	155	47.34	7	TSSSH	1MG7:B	SQAKA	234	238	46.96	7	CHHHH
3NUL:A	IESEL	127	131	92.24	8	HHTTC	2Z0B:A	LESEI	107	111	91.54	6	CSSEE
3NVS:A	GEVNL	14	18	48.98	6	EEEEC	2PNT:B	LNVEG	161	165	48.44	7	EECTT
3NVS:A	PLAQG	182	186	48.36	4	GGSSS	2XTS:C	GQALP	357	361	49	5	SCCCT
3NVS:A	LQAGT	158	162	52.84	7	CCSEE	1LBU:A	TGAQL	32	36	52.62	6	TTCCC
3NVS:A	GSISS	167	171	20.84	8	CSSCT	1K4Z:A	SSISG	1434	1438	19.9	10	CBSSC
3NVS:A	RLTNL	40	44	48.74	8	EEESC	3KC2:B	LNTLR	185	189	48.42	10	TTCCC
3NVW:A	LLAYL	26	30	7.12	10	HHHHH	1XEB:F	LYALL	18	22	7.56	11	HHHHH
3NYC:A	AAVLV	1192	1196	14.28	8	ESEEE	1NYK:A	VLVAA	186	190	13.42	9	EEEEC
3NYC:A	LLDAE	1115	1119	60.98	7	EECHH	2XHG:A	EADLL	196	200	61.58	5	SHHHH
3O0A:B	VVKPE	387	391	65.44	7	CEECS	2CLB:A	EPKVV	7	11	66.28	7	CCCCH
3O0L:A	LKLHG	97	101	46.76	7	EEECT	2O5H:B	GHLKL	63	67	46.58	9	TSCEE
3O0P:A	LSKGA	131	135	39.44	7	HTTSE	3ELF:A	AGKSL	338	342	39.28	8	TTCCT
3O0P:A	ERAAK	52	56	74.58	5	HHHHH	1JT2:A	KAARE	213	217	74.42	6	HHHHH
3O12:A	VTLET	28	32	23.64	11	EEEEC	2V5T:A	TELTV	264	268	23.32	11	TEEEE
3O1C:A	DLGLK	87	91	87.12	6	HTTCT	1I24:A	KLGLD	313	317	87.16	4	TTTCC
3O1N:B	SAATF	221	225	9.78	7	CCEEE	3AK9:J	FTAAS	148	152	10.16	9	HHHHH
3O1N:B	TDKPL	73	77	50.34	7	CSSCE	1W9M:A	LPKDT	423	427	50.08	5	SCTTE
3O22:A	YSQGS	132	136	36.74	9	EEEEE	1NOX:A	SGQSY	133	137	35.76	11	HHHHH
3O2R:B	VNEKS	75	79	68	8	SSHHH	3EWM:B	SKENV	70	74	68.08	8	HHTTC
3O2T:A	NDSKI	48	52	49.02	8	STHHH	2W1V:B	IKSDN	54	58	49.04	9	CHHHH
3O2T:A	QVQEL	57	61	51.32	9	HHHHH	2JER:H	LEQVQ	328	332	50.76	9	HHHHH

3O2T:A	RDIEL	102	106	61.84	10	HCGGG	3LLP:A	LEIDR	296	300	61.84	10	EEECT
3O46:A	DRSGL	174	178	71.94	7	HHHTC	3PC7:A	LGSRD	892	896	71.3	5	ESCCT
3O5N:G	SVDVE	686	690	54.42	7	EECTT	2IEC:A	EVDVS	65	69	54.24	6	EECCC
3O8Q:A	LRELRK	267	272	70.47	8	HHHHHH	2OX0:B	KRLERL	217	222	69.83	9	HHHHHH
3O8Q:A	GGRGC	58	62	23.22	10	TCCEE	3EVF:A	CGRGG	82	86	23.26	10	CTTCH
3O8Q:A	VTNRT	152	156	60.02	7	EEESS	1D4V:A	TRNTV	132	136	59.36	6	SSCCE
3OAB:C	AAIEA	6	10	36.68	8	HHHHH	2VWS:A	AEIAA	32	36	35.96	6	HHHHH
3OAJ:B	PDKTA	164	168	53.92	8	HHHHH	3LUM:D	ATKDP	126	130	54.5	8	CSBCT
3OAJ:B	SAGDI	191	195	34.26	4	CSSSS	1IAZ:A	IDGAS	9	13	33.72	6	EEGGG
3OBL:A	WNEGG	17	21	34.66	9	EEEEE	2WAO:A	GGENW	50	54	35.58	11	EEEEE
3OBQ:A	LHEWK	114	118	91.68	7	HHTCC	3VMK:B	KWEHL	78	82	91.8	5	GGTTS
3OD3:A	LPIPD	34	38	48.9	6	CCCCC	2G3R:A	DPIPL	1536	1540	48.24	6	SSCCT
3ODT:B	LLLFG	68	72	2.12	10	EEEEE	1UFO:D	GFLLL	51	55	1.86	10	TEEEE
3OIG:A	AHCIA	92	96	20.12	7	EECCC	3LMB:A	AICHA	105	109	20.1	9	EEEEC
3OIO:A	LRLNR	271	275	42.7	9	HHHHH	2VDU:D	RNLRL	106	110	42.84	8	EEEEE
3OIQ:B	LLDDV	233	237	24.9	9	HHHHH	1MML:A	VDDLL	223	227	25.04	7	TTEEE
3OIS:D	SWVGN	213	217	48.76	7	HHHGG	3ERB:A	NGVWS	174	178	48.64	6	TSCBS
3OIZ:A	VEGQL	408	412	39.06	10	EEEEE	1EJD:A	LQGEV	12	16	38.34	9	CEEEE
3OJ0:A	ILLVG	21	25	2.22	8	EEEEC	1GA8:A	GVLLI	155	159	1.48	10	EEEEE
3OJN:D	ILDPD	258	262	23.22	7	EECTT	3DA5:A	DPDLI	56	60	22.52	6	SBCEE
3OKX:A	RDGAN	121	125	47.42	7	EETTE	3PDY:B	NAGDR	704	708	48.3	9	HHHHH
3OLJ:A	AVRIE	296	300	26.5	11	HHHHH	2XFD:A	EIRVA	47	51	26	11	EEEEE
3OLQ:A	NLLVV	6	10	4.54	10	EEEEE	1W0H:A	VVLLN	156	160	4.44	11	EEEEE
3OMD:A	DLEEK	133	137	62.02	7	HHHTT	3PQS:A	KEELD	38	42	62.24	8	HHHHH
3ONH:A	PDVEV	543	547	76.08	7	CCCCC	2O71:A	VEVDP	187	191	75.2	7	TTCCT
3ONH:A	YSYPQ	469	473	47.42	7	HTCCS	3ERB:A	QPYSY	186	190	47.2	5	CGGGG
3OOP:A	KKDTP	59	63	99.96	6	TCCHH	2VU4:A	PTDKK	65	69	100.6	4	ECSCS
3ORK:A	SATNA	147	151	76.78	3	ETTSC	1O9W:A	ANTAS	113	117	75.8	4	TTCEE
3OV5:A	ATELS	105	109	31.34	9	HHHHH	1OF8:B	SLETA	47	51	30.9	11	HHHHH
3OZY:A	SYDLK	343	347	80.98	5	SCCCC	2X3G:A	KLDYS	74	78	80.08	6	CCCHH
3OZY:A	HTRGV	135	139	61.24	7	STTCE	1YIS:A	VGRTH	151	155	61.78	7	EEEET
3OZY:A	EPAIG	378	382	39.18	8	CCSBC	2WCJ:A	GIAPE	126	130	39.34	7	TCCCC
3OZZ:B	IGELH	76	80	33.18	9	EEEEC	3AAI:B	HLEGI	26	30	32.72	9	HHHHH

3OZZ:B	GGEEV	41	45	73.38	5	TCSHH	1VJQ:B	VEEGG	68	72	73.86	4	EETTC
3P02:A	TDDRL	73	77	98.72	5	CCCEE	1NNX:A	LRDDT	34	38	98.1	5	SCSSE
3P04:A	FAVVP	131	135	29.94	6	EEEEC	2GEF:A	PVVAF	590	594	30.32	6	EEEEE
3P1V:B	TCESL	200	204	28.74	9	HHHHH	3NSU:A	LSECT	517	521	29.46	7	GGGEE
3P2U:B	SGVVK	41	45	18.6	9	EEEEE	2Q7B:A	KVVGS	62	66	17.74	10	EEEEE
3P6B:B	DEGLC	43	47	63.8	7	TTSCC	3I6X:D	CLGED	57	61	63.52	6	HHSSC
3P8A:B	GGVAFA	90	95	12.92	8	GGTCTT	3HD5:C	AFAVGG	175	180	13.3	8	EEEETT
3P8B:D	YAILA	34	38	6.8	9	CEEEE	1Y7T:B	ALIAY	136	140	6.4	8	HHHHH
3P8K:A	IPVFK	249	253	51.62	4	SCGGG	2FL7:A	KFVPI	183	187	51.54	5	SCEEC
3PB6:X	ELFML	263	267	13.06	9	EEEEE	3BI1:A	LMFLE	668	672	13.24	10	HHHHH
3PB6:X	DPRAA	156	160	51.7	5	CTTSS	3ORU:A	AARPD	10	14	51.94	5	TTCCS
3PBT:A	GEPLA	70	74	37.22	5	SCEEE	3GFF:B	ALPEG	30	34	37.88	5	ECCTT
3PBT:A	AGSLV	246	250	1.34	9	EEEEE	1QHW:A	VLSGA	258	262	0.6	11	EEECC
3PGZ:B	VKVVE	65	69	45.54	8	HHHHH	2FGC:A	EVVKV	96	100	44.8	7	TEEEE
3PI6:A	YFSFF	177	181	3.1	9	HHHHH	3H5Z:A	FFSFY	322	326	2.14	9	EEEEE
3PI6:A	RQNAE	247	251	71.76	10	HHHHH	1N7S:D	EANQR	194	198	71.22	9	HHHHH
3PIK:A	LISEA	49	53	27.52	9	HHHHH	3UAW:A	AESIL	13	17	26.66	8	CSEEE
3PIS:A	KPVCG	7	11	29.92	9	CCEEB	2JKS:A	GCVPK	274	278	30	10	EEEEC
3PJP:B	FKAGT	1355	1359	49.7	8	BCCSC	2PQ5:A	TGAKF	87	91	50	7	CHHHH
3PMC:B	NPDLQ	103	107	85.5	8	CCCHH	3QQ8:B	QLDPN	626	630	86.36	6	GSCTT
3PPL:B	AELAKEA	365	371	53.557	7	HHHHHHT	3OQI:A	AEKALEA	100	106	53.31	7	HHHHHHH
3PT8:A	DLRTA	106	110	49.5	8	HHHHH	3SNK:A	ATRLD	30	34	49.78	7	HHHHH
3PT8:A	GGLTL	49	53	59.64	5	TTSCH	3BL4:A	LTLGG	13	17	59.16	7	EEECT
3PXL:A	APSSG	316	320	69.38	7	CSSTT	3G7R:B	GSSPA	82	86	68.46	7	HTCSS
3Q1P:B	SATHV	140	144	46.36	8	SCCCE	2CKX:A	VHTAS	627	631	45.92	6	HHHHH
3Q39:A	ECLLF	437	441	10.14	8	EEEEE	2P7I:B	FLLCE	240	244	9.56	8	EEEEE
3QAP:A	RLRNL	162	166	48.72	9	HHHHH	1T82:D	LNRLR	5	9	48.1	8	HHHHH
3QB8:B	TGKGL	120	124	69.82	5	CSSSH	1SR4:C	LGKGT	100	104	69.34	5	STTSE
3QB8:B	GLATK	123	127	41.04	5	SHHHH	1TP6:A	KTALG	52	56	40.72	7	HHHHH
3QFH:B	SANGK	196	200	28.8	9	HCBSS	2FHZ:A	KGNAS	22	26	29.7	7	TCTTT
3QHP:B	SVGIV	296	300	3.36	9	HTTCC	7ODC:A	VIGVS	191	195	3.46	8	EEEEE
3QM9:A	AGGQT	119	123	43.32	6	HHHHH	1LUC:A	TQGGA	164	168	44.1	4	STTSS
3QN1:B	VSPHF	208	212	50.88	5	EEEEE	1FYH:E	FHPSV	129	133	50.74	6	ECCGG

3QO4:A	CAALTD	123	128	37.3	6	CBTTBC	1HW7:A	DTLAAC	136	141	38.05	5	SSHHHH
3QQ8:B	RRFLA	588	592	74.28	8	EEEET	2CWZ:D	ALFRR	125	129	73.8	9	HHHHH
3QSD:A	GETLG	264	268	73.56	5	SCEEE	3B9W:A	GLTEG	131	135	72.72	5	SSCTT
3QSD:A	PSSFD	73	77	54.52	6	CSCEE	1OH4:A	DFSSP	12	16	54.76	8	CCCSH
3QSQ:A	VFLVR	538	542	15.96	9	EEEEE	3BNY:D	RVLFV	261	265	15.7	9	HHHHH
3QT9:A	IDQLI	374	378	43.24	8	HHHHH	1BD3:B	ILQDI	25	29	43.24	8	HHHHH
3QU5:B	IESIY	72	76	44.2	8	HHHHH	3E3V:A	YISEI	93	97	44.34	6	HHHHH
3QWW:A	EKKDL	144	148	94.28	7	HHHHH	1ZMA:A	LDKKE	23	27	94.2	7	HHTTC
3QXH:A	FISAT	3	7	4.24	7	EEEES	3LHI:A	TASIF	140	144	4.92	8	BTTBC
3QXH:A	LTQRLH	99	104	51.92	8	HHHHHH	1RTT:A	HLRQTL	129	134	51.67	9	HHHHHH
3QXH:A	NLKGN	175	179	64.22	6	CCCTT	3GMI:A	NGKLN	260	264	64.52	6	TTCSG
3QY3:A	TPVPD	123	127	53.8	6	CCCCH	1ESC:A	DPVPT	4	8	54.28	6	CEEEE
3QY9:D	PLLDE	66	70	61.72	5	GGGCS	2YXT:A	EDLLP	130	134	61.82	7	TTHHH
3QYJ:B	SKLGY	91	95	44.36	8	HHTTC	1IAZ:A	YGLKS	156	160	43.84	10	TTEEE
3QZB:A	EPTVT	94	98	57.2	5	CSEEE	2FYG:A	TVTPE	56	60	56.4	6	ESSCC
3QZB:A	KDEKV	29	33	83.6	5	TTCEE	2GFF:A	VKEDK	10	14	83.92	7	BCGGG
3QZR:A	LSLLR	8	12	64.56	18	HHHHH	3S7O:A	RLLSL	310	314	64.74	18	HHHHH
3R0V:A	GGALS	27	31	26.06	5	CCTTC	1KVE:B	SLAGG	206	210	25.16	5	EECSC
3R0V:A	RSGSG	16	20	64.58	8	EEECS	3YGS:P	GSGSR	48	52	65.24	7	TTCCH
3R0V:A	SGLPI	104	108	43.34	7	TTCCE	3KGR:A	IPLGS	39	43	42.5	5	EETTC
3R2Q:A	LSILL	15	19	2.74	8	HHHHH	1XIY:A	LLISL	47	51	1.88	9	EEEEC
3R2R:A	AALAK	98	102	34.68	8	HHHHH	3C8Z:B	KALAA	374	378	34.98	7	HHHHH
3R3R:A	TSSVV	23	27	40.8	8	TTCEE	1QB5:D	VVSST	39	43	41	7	EEETT
3R5G:B	ITKPL	322	326	54.5	6	CCSCC	3EYP:B	LPKTI	394	398	55.06	5	EEEEE
3R62:A	TLRTT	1213	1217	68	7	CSEEE	3OF4:C	TTRLT	32	36	67.94	7	HHHTC
3R62:A	TGESV	1193	1197	34.92	7	TTCEE	3P7X:A	VSEGT	145	149	35.22	6	CSBTT
3R62:A	QKLYS	1187	1191	77.84	6	CCSEE	1YLF:A	SYLKQ	49	53	77.22	5	HHHHH
3R8J:A	GPFSE	98	102	79.28	5	STTSC	3ACZ:A	ESFPG	288	292	78.94	5	TTSTT
3R9F:B	NKTAY	98	102	53.22	9	TTEEE	2XRH:A	YATKN	72	76	52.98	10	HHHHH
3R9F:B	ILFIK	76	80	16.44	9	EEEEE	3EYE:A	KIFLI	81	85	16.88	11	CEEEE
3RD5:A	DGVSG	80	84	57.28	6	HTCCC	2NX4:A	GSVGD	145	149	57.02	5	TSSCS
3RD7:A	GLATG	256	260	4.84	11	EEEEE	3RY2:B	GTALG	70	74	4.7	10	CEEEE
3RF7:A	TAVLC	151	155	15.68	11	CCEEE	3BT2:U	CLVAT	222	226	16.26	11	EEEEE

3RL5:A	FVCIS	60	64	0.04	10	EEEEB	3OII:A	SICVF	201	205	0.2	9	EEEEE
3RLS:B	EKVLN	106	110	81.9	6	CCCEE	2FAU:A	NLVKE	90	94	81.16	6	EEEEE
3RLS:B	THLWTI	40	45	22.52	12	CEEEEE	2PTV:A	ITWLHT	32	37	22.3	10	EEEEEC
3RNQ:A	TVSEG	43	47	59.6	6	EECTT	2PKD:F	GESVT	14	18	58.8	6	TSCEE
3RNQ:B	YLTVK	114	118	27.72	10	EEEEE	2P26:A	KVTLY	80	84	27.64	9	EEEEE
3RO3:A	DKAAE	230	234	45.38	7	CHHHH	3IJD:B	EAAKD	189	193	46.22	6	HHHHH
3RO3:A	LLIAK	222	226	40.48	8	HHHHH	1OQJ:B	KAILL	103	107	40.04	10	EEEEE
3RO3:A	RLLIA	221	225	24.9	9	HHHHH	3QR5:B	AILLR	118	122	24.7	7	EEEEE
3RPC:D	NSIPK	81	85	64.98	6	HHSCT	2VU9:A	KPISN	1039	1043	65.38	7	EECTT
3RPC:D	FIKGN	240	244	38.46	7	HHHHT	1Y7T:B	NGKIF	104	108	39.26	9	HHHHH
3RPD:B	AGAEI	341	345	31.34	7	HHHHH	3HIM:A	IEAGA	144	148	32.2	9	HHHHH
3RPF:B	YAKER	130	134	68.16	7	ECCTT	1ITV:A	REKAY	156	160	68.58	6	TTEEE
3RQT:A	SKVKN	447	451	62.74	7	TTEEC	2QE9:B	NKVKS	73	77	63.12	7	HHHHH
3RTL:A	GKTLY	423	427	55.38	7	TCCEE	1Z9F:A	YLTKG	68	72	55.02	8	HCCTT
3RZN:A	LEKIR	177	181	62.5	8	HHHHH	2NR5:F	RIKEL	49	53	62.02	10	HHHHH
3S0A:A	IDENS	76	80	73.88	6	SCHHH	3CZ6:A	SNEDI	678	682	73.88	6	CGGGG
3S40:D	IVPPL	29	33	2.06	8	HHHHH	2WAG:A	LPPVI	100	104	2.92	8	CCCEE
3S46:B	QAQDL	147	151	49	8	HHHHH	1YRE:A	LDQAQ	102	106	49.28	6	ECGGG
3S5B:A	PLDAV	274	278	39.36	6	CHHHH	1NKI:A	VADLP	12	16	40.2	8	ESCHH
3S5B:A	MINSY	198	202	26.3	8	SSCEE	1OMZ:A	YSNIM	311	315	25.56	6	CBCEE
3S6M:A	DNEFL	104	108	72.2	7	CCGGG	2OPC:A	LFEND	130	134	73.12	8	CBCCS
3S81:A	QIDSL	177	181	32.26	8	HHHHH	1FXK:C	LSDIQ	45	49	33.24	8	HHHHT
3S81:A	DAGAE	73	77	50.54	10	HTTCS	3C2E:A	EAGAD	218	222	50.62	9	HHTCS
3S90:D	NLVKT	1538	1542	49.32	7	HHHHH	2J3W:B	TKVLN	80	84	48.5	6	CSHHH
3S95:B	GQAIK	351	355	27.88	9	EEEEE	2OID:A	KIAQG	290	294	27.68	10	HHHHH
3S9J:A	RLVCG	236	240	13.74	8	EEEEE	1O6U:C	GCVLR	300	304	13.4	9	TCEEE
3S9J:A	TQKSL	72	76	71.82	5	CSTTC	2FUE:A	LSKQT	91	95	72.3	6	CCCCC
3S9J:A	PKLGN	271	275	40.34	7	CCCEE	3BB0:A	NGLKP	566	570	39.72	6	TTSCC
3S9J:A	ELVAE	34	38	59.88	6	EEEEE	3ZVL:A	EAVLE	236	240	60.18	8	HHHHH
3SAO:A	QAACS	148	152	54.24	4	CSSCC	2YHO:A	SCAAQ	410	414	55.1	3	HHHTT
3SD2:A	AKDYA	60	64	44.56	5	CCSCE	3HIN:A	AYDKA	197	201	44.14	6	HHHHH
3SF6:A	TRATR	160	164	60.04	6	CEEEE	3DCM:X	RTART	36	40	60.24	7	HHHHH
3SF6:A	TGEYP	359	363	67.02	3	STTSS	1W9H:A	PYEGT	310	314	67.64	4	CSSSC

3SF6:A	RGADD	14	18	63.64	5	CCHHH	1Q4U:A	DDAGR	131	135	63.46	5	CTTCC
3SF6:A	VADVA	183	187	16	7	GCSEE	3U5R:H	AVDAV	181	185	15.34	9	HHHHH
3SF6:A	ALHLG	314	318	23.94	8	HHHHH	3TKT:A	GLHLA	381	385	23.62	6	THHHH
3SG0:A	ANGGA	295	299	52.06	6	HTTTC	3EE4:A	AGGNA	25	29	52.66	7	HHHHH
3SG0:A	ERSAF	366	370	77.44	3	GGGCE	1YKW:A	FASRE	10	14	76.72	3	BCCGG
3SGG:A	NVRVV	537	541	39.5	8	TEEEC	3FVV:A	VVRVN	161	165	39.9	9	HHHHH
3SHG:A	GIKDQ	24	28	60.02	6	CCCCH	3FLP:E	QDKIG	146	150	60.3	7	CSBTT
3SHG:B	VVGEL	40	44	48.72	4	HHTSS	1KNQ:B	LEGVV	160	164	48.86	5	HHHHH
3SNK:A	DVATR	28	32	47.3	7	HHHHH	2YVQ:A	RTAVD	1454	1458	48.22	8	HHHHH
3SOV:A	GGLEDA	48	53	32.7	10	EEEEEE	3HTM:D	ADELGG	187	192	32.18	9	HHHHHH
3SOY:A	AVAEF	124	128	28.94	8	EEEEE	2XE5:A	FEAVA	256	260	29.92	10	EEEEE
3SX2:H	IVLIS	151	155	3.16	10	EEEEC	3O5Y:A	SILVI	100	104	2.58	9	EEEEE
3SZY:A	DAARV	279	283	43.14	7	TTSEE	3ED1:C	VRAAD	209	213	42.54	9	HHHHH
3SZY:A	TDPYV	288	292	60.14	4	SCTTC	3IB7:A	VYPDT	254	258	59.56	6	ECSSC
3T2C:A	KEVTD	78	82	41.92	10	HHHHH	3EVF:A	DTVEK	166	170	41.48	10	HHHHH
3TC7:A	LIEGS	246	250	54.66	4	HHHCC	3C8C:A	SGEIL	175	179	54.42	5	TCCEE
3TC7:A	NGSYE	90	94	42.58	7	CCCHH	2Y9W:D	EYSGN	97	101	41.76	6	EECSS
3TC7:A	PLIII	156	160	1.74	8	CEEEE	3QJG:L	IIILP	77	81	1.12	10	EEEEE
3TC7:A	VGLFI	78	82	1.3	10	SEEEE	3BB0:A	IFLGV	491	495	1.6	8	HHTTS
3TC7:A	LEFNK	38	42	69.3	8	HHHHH	3IVE:A	KNFEL	294	298	68.64	6	EEEEE
3TQE:A	AGLIK	283	287	26.16	8	HHHHH	3K1R:B	KILGA	438	442	26.42	9	HHHHH
3TU8:A	KTGST	14	18	73.3	6	HHTCC	3IMM:A	TSGTK	173	177	73.78	4	CCSSB
3TXS:A	SQMLL	61	65	38.18	11	HHHHH	3F95:A	LLMQS	673	677	39.12	10	EEEEE
3U23:A	QNEDE	122	126	78.6	5	SSTTB	3OJN:D	EDENQ	46	50	78.1	7	ECSSE
3U2U:B	ALVLG	19	23	0.74	8	HHHHH	3N0R:A	GLVLA	187	191	0.84	10	SEEEE
3U52:C	AELSA	315	319	44.46	7	HHHHH	1DVK:B	ASLEA	126	130	43.52	7	HTTTT
3U5R:H	SVAKA	134	138	47.88	7	HHHHH	2QTV:D	AKAVS	936	940	48.34	8	CCCCC
3U62:A	VEVKE	105	109	65.26	7	CCCCS	2GIA:G	EKVEV	143	147	65.18	5	SCEEE
3U7I:D	EAASR	205	209	42.62	9	HHHHH	3V8H:A	RSAAE	67	71	41.68	9	CBHHH
3U7I:D	LPLHN	99	103	29.04	7	EECBT	2QKP:A	NHLPL	337	341	28.52	9	HHSSS
3U7Q:A	VKGAEL	169	174	60.52	7	HHHHHH	2QIF:B	LEAGKV	37	42	61.47	5	TTTTEE
3U7Q:D	GFPIF	470	474	11.74	8	SSCCC	3LQK:A	FIPFG	152	156	11.12	9	ECCEE
3U7Q:D	PSFVG	187	191	24.64	4	CTTSS	1AQ0:B	GVFSP	133	137	24.42	5	EECSS

3U7Q:D	VDAIL	406	410	30.76	7	HHHHH	1TQH:A	LIADV	170	174	29.94	6	HHHHH
3U7Q:D	HKGKE	457	461	78.34	8	HHCGG	3LFP:A	EKGKH	39	43	78.58	7	HHTSS
3U7Q:D	VYIGK	421	425	37.18	5	EEESC	3SEE:A	KGIYV	102	106	36.76	7	SSEEE
3U7Q:D	LGYEG	485	489	11.74	10	SHHHH	1O04:G	GEYGL	478	482	12.3	8	TGGGG
3U80:A	LDTLR	28	32	45.92	8	HHHHH	2OS5:A	RLTDL	21	25	46.18	10	HHHHH
3U9Q:A	TREFL	349	353	45.34	7	EHHHH	3ZYL:A	LFERT	63	67	44.34	8	HHHHT
3UAW:A	TGEET	208	212	63	6	TCCBC	2FXA:B	TEEGT	107	111	63.14	6	CHHHH
3UJC:A	NKNKL	137	141	43.64	10	HHHHH	3A7O:A	LKNKN	91	95	44.16	11	HHHHH
3UJC:A	LALSL	131	135	35.54	7	GGSCH	3CNR:A	LSLAL	11	15	35.92	6	EEEEC
3UO3:B	LLEKQ	126	130	65.3	8	HHHHH	1W23:A	QKELL	23	27	65	7	HHTSS
3V2U:D	SFVIA	246	250	7.76	7	EEEEE	2W8T:A	AIVFS	128	132	6.8	9	EEEES
3V4K:A	ELCFL	259	263	18.4	6	HHHHH	2E56:A	LFCLE	146	150	18.46	7	EEEEE
3V5A:A	NLDGG	393	397	5.88	11	EECHH	1ZWX:A	GGDLN	227	231	4.9	9	EEECC
3V7P:A	IAVGK	358	362	44.02	5	CCTTS	3GFP:A	KGVAI	433	437	43.4	6	CCEEE
3V7P:A	AKRAL	189	193	35.86	8	HHHHH	3K5I:B	LARKA	237	241	35.64	8	HHHHH
3V7P:A	RALDIA	191	196	33.98	9	HHHHHH	2IMF:A	AIDLAR	36	41	33.43	9	ECCHHH
3V7P:A	EDLQA	124	128	49.04	9	TTHHH	1MHM:B	AQLDE	43	47	49.24	8	HHHHH
3V8H:A	QLERE	267	271	71.84	6	HHHSC	3GI7:A	ERELQ	52	56	71.98	8	HHHHH
3V8H:A	ATRSA	65	69	18.26	12	TCCBH	3RF7:A	ASRTA	148	152	17.86	10	TCSCC
3V9O:A	VDLFV	49	53	1.9	9	EEEEE	2BHU:A	VFLDV	209	213	2.24	9	EEEEE
3VHV:A	LKSQA	886	890	67.08	6	CTTHH	3ZRI:A	AQSKL	15	19	66.26	5	HHHHH
3VMK:B	SISMN	267	271	12.46	12	EEEEC	3EJ9:F	NMSIS	49	53	12.4	12	GEEST
3ZQI:A	EVGIE	19	23	55.36	9	HHHHH	3IJW:B	EIGVE	224	228	55.5	7	HHHHH
3ZQU:A	CSTGT	103	107	24.2	7	ECHHH	2EX4:B	TGTSC	61	65	24.74	8	CCCSE
3ZQU:A	YGLRL	19	23	23.5	8	HHHHH	1LC5:A	LRLGY	223	227	22.6	10	TCCEE
3ZQU:A	SAVAT	109	113	33.2	7	HHHHH	1H16:A	TAVAS	75	79	32.54	5	EEEEC
3ZRX:A	ERLRR	320	324	59.54	9	HHHHH	1O9G:A	RRLRE	132	136	60.08	11	HHHHH
3ZS9:C	GASSR	525	529	36.58	9	HHHHH	2V7Q:J	RSSAG	9	13	37.38	7	CCCTT
3ZUD:A	GPVIN	88	92	0.2	9	CCEEE	1O04:G	NIVPG	219	223	0.08	9	EECCB
3ZVL:A	APVSD	471	475	64.32	7	CCCCH	2QKP:A	DSVPA	357	361	63.32	6	CCSCG
3ZW5:B	IEEGP	113	117	63.22	5	CCEEE	3E5U:A	PGEEI	46	50	62.94	3	TTCCC
3ZZP:A	IQRAL	68	72	43.38	8	HHHHH	3BT5:A	LARQI	157	161	43.86	8	HHHHH
4A4Y:A	FRLLV	12	16	30.32	8	EEEEE	1HXA:A	VLLRF	224	228	29.56	7	HHHHH

4A56:A	ASVAAGA	40	46	13.843	9	HHHHHHH	1ZR3:A	AGAAVSA	255	261	14.33	11	TCEEEEE
4ACJ:A	IEKLA	647	651	59.16	7	HHHHH	1FG7:A	ALKEI	282	286	59.28	5	HHHHS
4ACJ:A	KEDFF	787	791	86.88	9	SSEEE	2WM9:A	FFDEK	233	237	86.34	7	CCCHH
4AJJ:A	ELALV	46	50	5.62	10	EEEEE	2FOM:B	VLALE	97	101	5.68	10	EEECC
4DFA:A	VYKGN	114	118	89.36	4	CCTTS	1KLX:A	NGKYV	69	73	88.36	4	HCSSS
4FIV:A	DNSLI	94	98	75.66	7	TCCCS	1JY5:A	ILSND	70	74	76.14	6	GGHHH
8A3H:A	AAIFV	222	226	2	8	CCEEE	1KGD:A	VFIAA	850	854	2.8	8	EEEEC
8A3H:A	FYAGT	201	205	24.4	7	EETTS	3GJU:A	TGAYF	356	360	23.84	9	HHHHH
8A3H:A	SSGGY	68	72	29.1	7	STTST	3KV1:A	YGGSS	108	112	29.24	9	HTCEE
8A3H:A	AELSP	287	291	44.1	8	GGBCH	1V8D:C	PSLEA	84	88	43.44	6	CCHHH
8A3H:A	EAAID	87	91	47.74	8	HHHHH	3SG0:A	DIAAE	104	108	47.54	6	HHHHH
